# A Comparison of Cellular Uptake Mechanisms, Delivery Efficacy, and Intracellular Fate between Liposomes and Extracellular Vesicles

**DOI:** 10.1002/adhm.202300319

**Published:** 2023-07-09

**Authors:** Timea B. Gandek, Luke van der Koog, Anika Nagelkerke

**Affiliations:** ^1^ Pharmaceutical Analysis Groningen Research Institute of Pharmacy University of Groningen P.O. Box 196, XB20 Groningen 9700 AD The Netherlands; ^2^ Molecular Pharmacology Groningen Research Institute of Pharmacy University of Groningen P.O. Box 196, XB10 Groningen 9700 AD The Netherlands

**Keywords:** drug delivery systems, endocytosis, functionalization, intracellular trafficking, targeting

## Abstract

A key aspect for successful drug delivery via lipid‐based nanoparticles is their internalization in target cells. Two prominent examples of such drug delivery systems are artificial phospholipid‐based carriers, such as liposomes, and their biological counterparts, the extracellular vesicles (EVs). Despite a wealth of literature, it remains unclear which mechanisms precisely orchestrate nanoparticle‐mediated cargo delivery to recipient cells and the subsequent intracellular fate of therapeutic cargo. In this review, internalization mechanisms involved in the uptake of liposomes and EVs by recipient cells are evaluated, also exploring their intracellular fate after intracellular trafficking. Opportunities are highlighted to tweak these internalization mechanisms and intracellular fates to enhance the therapeutic efficacy of these drug delivery systems. Overall, literature to date shows that both liposomes and EVs are predominantly internalized through classical endocytosis mechanisms, sharing a common fate: accumulation inside lysosomes. Studies tackling the differences between liposomes and EVs, with respect to cellular uptake, intracellular delivery and therapy efficacy, remain scarce, despite its importance for the selection of an appropriate drug delivery system. In addition, further exploration of functionalization strategies of both liposomes and EVs represents an important avenue to pursue in order to control internalization and fate, thereby improving therapeutic efficacy.

## Graphical abstract

Lipid‐based vesicles represent the holy grail of nanosized drug delivery systems. In a head‐to‐head comparison between artificial and natural, cell‐derived vesicles, a key question emerges: Would better efficacy be achieved by liposomes, or would the intrinsic composition of extracellular vesicles (EVs) be advantageous? Here, how cellular uptake mechanisms and intracellular delivery differ between liposomes and EVs are explored.

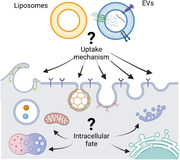

## 1 Introduction

Traditionally, the vast majority of human diseases have been treated with drugs that typically do not require active transport to cross the plasma membrane of cells. However, for the action of new promising therapeutic macromolecules (e.g., nucleic acids and proteins) active intracellular delivery across the plasma membrane is often required.^[^ ^1^ ^]^ This semipermeable membrane is composed of a heterogeneous lipid bilayer, which acts primarily as a structure to separate intracellular structures from the extracellular space.^[^ ^2^ ^]^ As such, the cell membrane may act as a barrier preventing therapeutic macromolecules from accumulating intracellularly, thereby negatively influencing the effectiveness of therapy.

To overcome this barrier, lipid‐based nanoparticle systems have emerged as a promising approach for effective drug delivery of numerous therapeutics, such as small molecules, nucleic acids, and proteins. The application of liposomes, a class of synthetic lipid‐based nanoparticles, as a drug delivery system has been studied in depth. Liposomes are spherical‐shaped vesicles that can incorporate hydrophilic drugs within their aqueous core and hydrophobic drugs within their lipid bilayer.^[^ ^3^ ^]^ A plethora of different lipids can be used to formulate these vesicles. A list of all the lipids mentioned in this review, as well as their abbreviations, can be found in Table S1 (Supporting Information). As a drug delivery system, liposomes have several advantages, such as biocompatibility, the ability to carry large drug loads, and the tunability of a wide range of physicochemical properties.^[^ ^4^, ^5^, ^6^, ^7^ ^]^ In addition, liposomes can alter the pharmacokinetics of drugs by protection from early inactivation, degradation, and dilution in the blood circulation.^[^ ^8^ ^]^ More recently, extracellular vesicles (EVs) have emerged as the more complex, naturally occurring counterparts of liposomes. EVs are cell‐secreted nanoparticles that can have a wide range of origins. Potentially, they can be secreted by all organisms, albeit in various forms. EVs from mammalian cells are characterized by the presence of a complex lipid bilayer and can be divided into two main subgroups: microvesicles and exosomes. Microvesicles are formed by direct budding from the plasma membrane and typically vary in size from 100 to 1000 nm. Conversely, exosomes have reported sizes between 30 and 150 nm and are generated by the reverse budding of endosomal multivesicular bodies. Upon fusion of these bodies with the plasma membrane, exosomes are secreted into the extracellular space. Importantly, EVs derived from bacteria, known as outer membrane vesicles, are thought to be more equivalent to mammalian microvesicles than exosomes. For mammalian microvesicles and exosomes, the overlap in size and other properties, such as marker expression and density, makes it challenging to separate the two. This, in addition to the variation that is likely present in these EV subtypes, makes for a highly heterogeneous vesicle population. Furthermore, in biofluids, such as blood, the presence of large quantities of additional particulates and proteins complicates the isolation of EVs. Therefore, we will here refer to both subgroups with the more generic term EVs, as recommended by the International Society of Extracellular Vesicles.^[^ ^9^ ^]^ EVs have captured the attention of many scientists and numerous studies have investigated the suitability of EVs as advanced drug delivery entities.^[^ ^4^, ^7^, ^10^ ^]^

The efficiency of drug delivery systems depends, among other factors, on the internalization mechanism by recipient cells and the subsequent intracellular trafficking. Ideally, drug delivery entities transport their drug load safely towards specific target cells where, upon internalization by target cells, they release the therapeutic cargo at the site of action. However, degradation in the endo‐lysosomal system poses a potential risk. This suggests that the cellular fate and efficacy of drug delivery systems are highly dependent on their escape from this recycling system. In depth analysis of the similarities and differences by which liposomes and EVs interact with target cells and subsequently release their cargo is a highly relevant area in this respect, but remains limited to date. In this review, we first assess the different mechanisms by which liposomes and EVs are internalized by cells. Subsequently, we address how certain plasma membrane components, such as heparan sulfate proteoglycans (HSPGs) and lipid raft domains, are associated with lipid nanoparticle uptake. In addition, we review the intracellular fate of the drug load upon internalization and establish critical mediators on the side of both lipid nanoparticles and recipient cells that contribute to efficient drug delivery. We will thereby compare liposomes and EVs and assess what is needed for further development of lipid nanoparticle‐based therapies.

## 2 Cellular Internalization Mechanisms of Liposomes and EVs

### 2.1 Endocytosis Mechanisms and Techniques Currently Used to Assess Uptake

**Figure 1:**
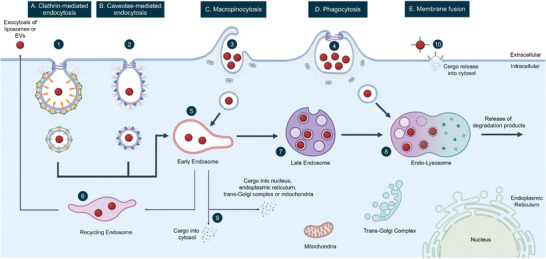
Overview of internalization mechanisms of liposomes and EVs into cells. A) Clathrin‐mediated endocytosis is directed by activating protein 2 (AP2) adaptor complex that recruits clathrin from the cytosol, thereby initiating the formation of clathrin‐coated pits. Subsequently, clathrin proteins are assembled around the forming vesicle, which oligomerize to yield highly curved invaginations, namely clathrin‐coated pits (1). B) Caveolae‐mediated endocytosis requires caveolin and cavin proteins for the formation of vesicle coatings and for the subsequent intracellular trafficking (2). C) Macropinocytosis is driven by actin polymerization through phosphoinositide 3‐kinase (PI3K) activity, leading to membrane ruffling that takes up a large volume of extracellular fluid together with liposomes/EVs. The formed endocytic vesicles, also known as macropinosomes, are trafficked further inside the cell (3). D) Similar to macropinocytosis, phagocytosis also relies on the PI3K activity through which the membrane ruffles bound tightly around the material being taken up, resulting in endocytic vesicles, called phagosomes (4). After the formation of vesicular membranes, clathrin‐, caveolae‐mediated endocytosis, and phagocytosis require dynamin to facilitate vesicle budding by scission. Subsequently, the coated endocytic vesicles and macropinosomes fuse with early endosomes (5), where the lipid nanoparticle can either be exocytosed from the cell via recycling endosomes (6) or undergo further trafficking along the endo‐lysosomal pathway for degradation (7). Lipid nanoparticles taken up through phagocytosis are directly transported to lysosomes (8). In a minority of cases, the vesicles’ cargo may escape during transport from early to late endosomes and be released into the cytosol or gain access to cellular components, such as the nucleus, endoplasmic reticulum, mitochondria or the trans‐Golgi complex (9). E) Membrane fusion represents the only mechanism that allows direct cytosolic delivery of the vesicles’ cargo (10).

The vast majority of drug delivery systems are taken up by cells through endocytosis, an overview of which is depicted in **Figure** **1**.^[^ ^11^, ^12^ ^]^ Endocytosis is a fundamental process in cells for uptake of nutrients and macromolecules, as well as for bacteria and viruses to overcome the plasma membrane and reach the intracellular milieu.^[^ ^13^ ^]^ This internalization mechanism can be recognized by the inward budding of the plasma membrane engulfing a cargo that undergoes further intracellular trafficking.^[^ ^14^ ^]^ Cargo internalization is triggered by interactions with cellular surface receptors, consequently inducing endocytosis through signaling cascades to stimulate the formation of plasma membrane invagination. The generated inward budded membrane is then pinched off, herein referred to as endocytic vesicle, which undergoes further fusion with other organelles or fission events.^[^ ^15^ ^]^ Fusion activity consist of the docking of an endocytic vesicle with a cellular compartment and subsequent merging, whereas fission involves the separation of one endocytic compartment into two. Such processes are governed by factors, such as SNARE proteins, tethers and Rab GTPases.^[^ ^16^, ^17^ ^]^ Overall, several uptake mechanisms have been identified, with underlying molecular processes remaining to be fully elucidated.^[^ ^13^ ^]^ In this section, we briefly describe the various endocytosis mechanisms and the techniques by which these can be studied in the context of liposomes and EVs.

Clathrin‐mediated endocytosis is among the most well‐documented internalization mechanism, entailing the formation of clathrin‐coated pits through the assembly of specific proteins, called clathrins (see Figure 1A). Through surface receptor interactions, this pathway is known to internalize important factors for cell metabolism, such as the iron‐binding protein transferrin and low‐density lipoprotein.^[^ ^18^, ^19^, ^20^ ^]^ The second well‐studied mechanism is caveolae‐mediated endocytosis, which is characterized by a flask‐shaped invagination of the cell membrane and a vesicle coat composed of caveolins (see Figure 1B). This pathway is enriched in membrane components, namely lipid rafts, including sphingolipids and cholesterol.^[^ ^21^, ^22^, ^23^ ^]^ In addition to these mechanisms, there are several other endocytic processes classified under the umbrella term of clathrin‐ and caveolae‐independent endocytosis. These pathways are less well understood, and the characteristic proteins involved in one endocytic route seem to contribute to others as well. All these mechanisms require the formation of a pit in the plasma membrane. However, macropinocytosis and phagocytosis manifest through the formation of intricate membrane protrusions.^[^ ^24^ ^]^ Macropinocytosis and phagocytosis are endocytosis mechanisms driven by actin polymerization, which subsequently enables membrane ruffling to engulf cargoes that are larger than for the other mechanisms (see Figure 1C,D).^[^ ^13^, ^25^ ^]^ Furthermore, in addition to endocytosis, direct fusion of lipid nanoparticles with the plasma membrane has been suggested as a potential entry route (see Figure 1E). Mounting evidence suggests that lipid nanoparticles exploit all these routes to gain entry inside the cell for drug delivery purposes, hence the growing interest in the complete elucidation of the underlying molecular processes.

In this respect, to visualize lipid‐based nanoparticles upon cellular entry and during intracellular trafficking, staining is a key method being employed. Most frequently, labeling is carried out using either lipophilic dyes that locate in the vesicle membrane (e.g., DiO, dialkylcarbocyanine dyes such as DiR, PKH26, rhodamine R18) or membrane permeable chemical compounds that are sequestered in the cytosolic compartment (e.g., carboxy‐fluorescein succinimidyl ester and 5(6)‐carboxyfluorescein diacetate).^[^ ^26^, ^27^ ^]^ After labeling of liposomes or EVs, recipient cells are treated either with pharmacological inhibitors to interfere with particular pathways or with antibodies to hinder receptor–ligand interactions, resulting in inhibition of that particular mechanism. It is important to note that pharmacological inhibitors lack full specificity for hindering a particular internalization pathway.^[^ ^11^, ^28^, ^29^, ^30^, ^31^ ^]^ As such, data interpretation of uptake should be performed with great care as pharmacological inhibitors may interfere with multiple uptake pathways.^[^ ^11^ ^]^ Alternatively to treatment with pharmacological inhibitors, cells can be subjected to knockdown of genes that play a role in endocytosis, which may lead to a more specific inhibition of a given uptake mechanism.^[^ ^20^ ^]^ A different approach to establish the internalization route of drug delivery systems is by studying colocalization of the nanocarriers with confirmed endocytosis model substrates, compounds for which the uptake mechanism is well established. Examples of such endocytic markers include low‐density lipoprotein and transferrin for clathrin‐mediated endocytosis, cholera toxin subunit B for caveolae‐mediated endocytosis, dextran for macropinocytosis, and latex beads for phagocytosis.^[^ ^20^, ^32^ ^]^ This approach could be used in addition to pharmacological inhibitors to ensure a more accurate interpretation. After uptake, direct visualization of particle trafficking is assessed using optical and electron microscopy techniques.

In the upcoming sections, we will describe the different internalization mechanisms through which liposomes and EVs are taken up by cells. Subsequently, we will discuss the constituents of these internalization mechanisms, as well as how specific entry routes have been pinpointed over the years using pharmacological inhibitors. We will also address the intracellular trafficking of lipid‐based nanocarriers and the final fate of the encapsulated cargo, while evaluating the experimental conditions used. Specifically, we compare the uptake of liposomes and EVs and explore strategies to potentiate their cellular uptake.

#### 2.1.1 Clathrin‐Dependent Endocytosis

**Figure 2:**
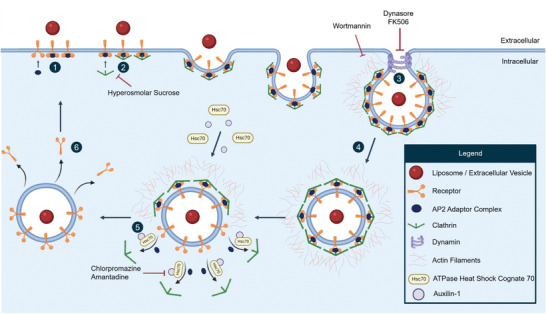
Overview of clathrin‐dependent endocytosis. Clathrin‐mediated internalization is mostly driven by receptors, such as low density lipoprotein and transferrin, which upon ligand‐receptor interaction, initiate the formation of clathrin‐coated pits by the recruitment of AP2 adaptor complexes (1). Subsequently, these complexes recruit clathrin proteins to initiate vesicle coating (2) that is pinched off the plasma membrane through scission by dynamin (3). These coating and scission processes are supported by actin filaments, mediated by PI3K activity (4). Next, the vesicle loses its protein coat by recruiting ATPase heat shock cognate 70 and auxilin, after which the vesicle is free to undergo intracellular trafficking (5) and the receptors are recycled back to the plasma membrane (6). Blocking of clathrin‐associated endocytosis can be achieved through membrane depletion of AP2 proteins via chlorpromazine or amantadine treatment, disruption of clathrin assembly via hyperosmolar sucrose treatment, interference with dynamin via dynasore or FK506 treatment, or obtrusion of actin rearrangement via wortmannin treatment.

**Figure 3:**
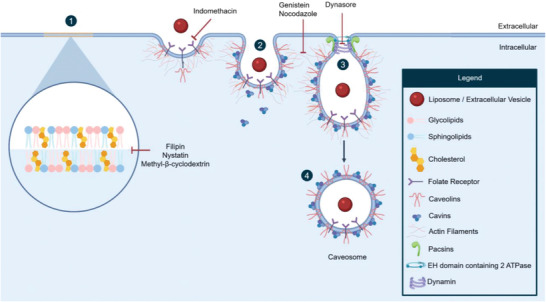
Overview of caveolae‐dependent endocytosis. Caveolae‐mediated internalization is initiated in membrane domains rich in glycolipids, sphingolipids, and cholesterol (1). During uptake, cavins anchor caveolins to the cytoskeleton, while stabilizing caveolae formation (2). Once the caveolae assembly is oligomerized, the structure is stabilized by EH Domain Containing 2 ATPase and BAR protein domain‐containing syndapin/Pacsin2. Consequently, the membrane is pinched off through the GTPase activity of dynamin (3). The vesicle containing the internalized cargo undergoes intracellular trafficking as a so‐called caveosome (4). Several inhibitors have been employed to inhibit this mechanism by interfering with the cholesterol in the plasma membrane such as filipin, nystatin, and methyl‐β‐cyclodextrin or depleting dynamin through actin filament disruption through genistein and nocodazole treatment or interfering with membrane scission using dynasore treatment or blocking folate receptors with indomethacin treatment.

**Figure 4:**
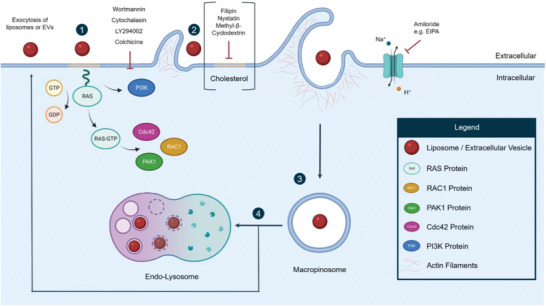
Overview of macropinocytosis. During macropinocytotic uptake, activation of intracellular signals induces membrane ruffling around the cargo to be engulfed. The main signaling factors involved in macropinocytosis are members of Ras superfamily GTPases, including Rac1, PAK1 and Cdc42 (1). Membrane ruffles are formed through actin polymerization via PI3K activity (2) and can fold back, fusing with the base of the plasma membrane, therefore entrapping the cargo. The formed vesicle, called the macropinosome (3), can be transported to lysosomes for degradation or to the plasma membrane for recycling (4). Standard inhibitors of macropinocytosis are wortmannin, cytochalasin D, LY294002 and colchicine, all of which interfere with actin re‐organization. Other inhibitors, such as amilorides, alter cellular pH through interference with Na^+^/H^+^ exchanger, thus blocking macropinocytosis.

**Figure 5:**
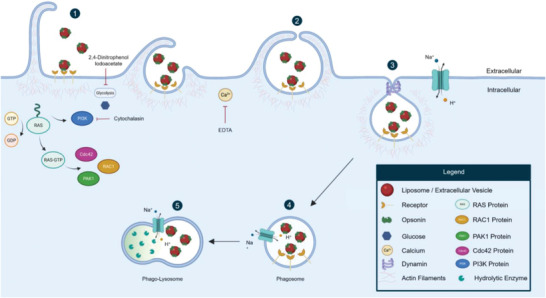
Overview of phagocytosis. In close proximity, phagocytic receptors from the surface of cells recognize opsonins attached to the surface of liposomes/EVs (1). This protein‐receptor binding initiates phagocytosis, which in turn triggers membrane remodeling through the activity of actin filaments mediated by Ras superfamily GTPases, including Rac1, PAK1, and Cdc42. The membrane extends around the cargo through membrane ruffling or lamellipodia formations (2) and it is ingested through the activity of dynamin (3). Then, the resulting membranous vesicle containing the internalized lipid‐based nanocarrier, called the phagosome (4), is trafficked to lysosomes for degradation (5). Several approaches have been established to block phagocytosis, such as 2,4‐dimitropherol and iodoacetate, which interfere with glycolysis, EDTA that chelates calcium, and cytochalasin B that disrupts actin polymerization.

One of the most extensively studied endocytosis mechanisms is clathrin‐mediated endocytosis.^[^ ^29^, ^33^, ^34^ ^]^ **Figure** **2** provides an overview of clathrin‐dependent endocytosis and the factors involved in this mechanism, as well as the inhibitors used to establish this pathway. This entry mechanism is involved in various cellular activities, including maintenance of homeostasis, nutrient transport, and sampling the environment for growth and guidance cues.^[^ ^33^, ^35^ ^]^ As such, it is one of the main routes to provide nutrients for cells, but it is also thought to be the main internalization pathway exploited by various viruses, such as the influenza virus, reoviruses, and Semliki Forest virus.^[^ ^13^ ^]^ In the early stages of cargo engulfment, the formation of clathrin‐coated pits proceeds through the assembly of various protein constituents around the curved membrane. Major constituents of this assembly are clathrin‐adaptor proteins, such as the activating protein 2 (AP2) complex, the accessory protein AP180, and epsins. These proteins bind to lipids on the cytosolic side of the plasma membrane and recruit other constituents at the site of the invagination for the formation of a protein coat (see Figure 2‐1).^[^ ^18^ ^]^ The coat consists of various scaffold proteins. Clathrin and epidermal growth factor receptor pathway substrate 15 are key examples, which polymerize and stabilize the vesicle formation, respectively (see Figure 2‐2).^[^ ^18^ ^]^ This assembly further facilitates plasma membrane bending, stimulating the formation of a basket, which is also known as the clathrin‐coated pit.^[^ ^36^ ^]^ After formation of the clathrin‐coated pit, the resulted vesicle is separated from the plasma membrane through a process called scission, mediated by the GTPase activity of dynamin.^[^ ^37^ ^]^ This enzyme assembles in a helical collar‐like structure around the neck of the membranous vesicle, which, upon binding, undergoes conformational changes, leading to constriction and catalyzation of vesicle fission (see Figure 2‐3).^[^ ^19^, ^36^ ^]^ In some cases, it has been reported that during this process actin is recruited to facilitate membrane budding.^[^ ^33^ ^]^ Next, the vesicle undergoes intracellular trafficking across actin filaments mediated by phosphoinositide 3‐kinase (PI3K) (see Figure 2‐4).^[^ ^18^, ^33^, ^38^ ^]^ Subsequently, the process of uncoating begins by recruiting ATPase heat shock cognate 70 and its co‐factor, auxilin (see Figure 2‐5). This vesicle is subsequently transferred to an early endosome, also known as a sorting endosome, where the cargo can either be transported back to the plasma membrane or follow the endo‐lysosomal pathway (see Figure 2‐6).^[^ ^39^ ^]^ Herein, early endosomes can undergo fusion or fission events in order to transfer the encapsulated cargo to other organelles. Clathrin‐mediated internalization is believed to transfer the enclosed cargo to lysosomes, followed by degradation through the activity of resident hydrolytic enzymes. Nonetheless, the therapeutic action of cargo can be maintained, as will be addressed later in this section.

Several pharmacological inhibitors have been employed to assess the involvement of clathrin‐mediated endocytosis in the internalization of liposomes and EVs. Clathrin‐dependent uptake can be established through hampering with the active/inactive state of AP2 complex, which leads to its depletion from the plasma membrane, consequently halting the formation of clathrin‐coated pits. Chlorpromazine is one such compound, which upon incubation with cells, assembles the AP2 complex at the site of endosomal membranes, where, under normal conditions, the assembly does not occur.^[^ ^40^ ^]^ Another such example is amantadine, which stabilizes clathrin‐coated vesicles and halts the recycling of clathrin proteins to the plasma membrane.^[^ ^41^ ^]^ Other inhibitors, such as hyperosmolar sucrose, disrupt the assembly of clathrin proteins, thereby inhibiting clathrin‐mediated endocytosis.^[^ ^42^ ^]^ Additionally to these inhibitors, dynasore and FK506 treatment can also interfere with clathrin‐mediated entry by hindering the GTP activity of dynamin and blocking vesicle scission.^[^ ^43^ ^]^ Some studies also used wortmannin treatment to inhibit clathrin‐associated pathways by interfering with actin polymerization.^[^ ^44^, ^45^ ^]^ Alternatively to inhibitors, co‐localization analysis with well‐established clathrin‐mediated markers, such as low‐density lipoprotein and transferrin, has also been used to identify uptake through this pathway.^[^ ^20^, ^46^ ^]^ However, transferrin receptors have been reported to be present in caveolae‐associated pathways as well.^[^ ^47^ ^]^

Over the years, several studies investigated clathrin‐mediated endocytosis of both liposomes and EVs, which we will compare in detail below. The main results of these studies are also summarized in Table S2 (Supporting Information). Rajaganapathy et al. studied the uptake of colloidal gold‐containing liposomes of unspecified composition by UROtsa bladder cells. The authors observed that chlorpromazine treatment dose‐dependently inhibited liposome uptake, in which at the highest chlorpromazine concentration (28 × 10^‐6^ m) liposome detection was almost completely absent.^[^ ^48^ ^]^ Similarly, in a study by Lakkaraju et al. the internalization of DOPC:DOPG (87.5:12.5 mol%) liposomes containing Cy3‐labeled oligonucleotides was abolished after interfering with clathrin‐associated pathways using hyperosmolar sucrose treatment in hippocampal neurons.^[^ ^49^ ^]^ This study also demonstrated that clathrin‐mediated endocytosis of DOPC:DOPG liposomes involved interactions with low‐density lipoprotein receptors and that the uptake was mediated by dynamin and PI3K.^[^ ^49^ ^]^ Still, in the majority of cases, clathrin‐dependent endocytosis contributes only partly to the internalization of lipid nanoparticles. Zhaorigetu et al. showed that the uptake of DMPC:DMPG (7:3 molar ratio) liposomes by human coronary artery endothelial cells (HCAECs) is partly mediated by clathrin. Amantadine treatment inhibited liposome uptake by 46%, compared to untreated cells.^[^ ^50^ ^]^ As such, differences in the lipid composition tested in combination with the type of recipient cells used, appear to dictate the extent to which clathrin‐mediated endocytosis is responsible for the internalization of liposomes.

In addition, environmental conditions can also influence clathrin‐dependent uptake.^[^ ^39^ ^]^ For example, stimulation of recipient cells with tumor necrosis factor α to recreate inflammatory conditions, enhanced uptake of DMPC:DMPG liposomes by approximately threefold.^[^ ^50^ ^]^ This could be exploited in therapeutic applications. Another condition is the presence or absence of serum during cellular uptake studies. Digiacomo et al. observed that during circulation of nanoparticles protein molecules adhered to their surface, forming the so‐called protein corona, which consequently affected the cellular uptake mechanism.^[^ ^51^ ^]^ Preincubation of DOTAP:DOPC:DOPE:DC‐cholesterol (1:1:1:1 molar ratio) liposomes with human plasma allowed the formation of such a protein corona. This in turn changed the initial macropinocytotic uptake into clathrin‐mediated endocytosis, most probably due to the abundant presence of apolipoproteins in the protein corona composition, which target low‐density lipoprotein receptors.^[^ ^51^ ^]^ Although there are ways to improve liposome uptake by changing lipid composition or environmental conditions, increased uptake does not always improve therapeutic outcomes. This is exemplified in the work of Alshehri et al. who used cationic lipid–DNA‐based vectors known as lipoplexes, to study cellular uptake and siRNA‐mediated silencing efficiency in human lung cancer epithelial A549 cells. Following chlorpromazine treatment, the uptake of Cy3‐labeled siRNA‐loaded DOPE:DC‐cholesterol (1:1 molar ratio) lipoplexes decreased by 34%. Meanwhile, silencing of luciferase was hardly achieved in luciferase‐expressing recipient cells.^[^ ^52^ ^]^ Overall, clathrin‐mediated endocytosis not only contributes to the uptake of lipid‐based artificial vesicles but may in some cases also influence subsequent transfection efficiency.

Similar to liposomes, clathrin‐mediated uptake is also being exploited by EVs to gain entry into cells.^[^ ^12^ ^]^ Eguchi et al. showed that this uptake mechanism was responsible for the enhanced uptake of EVs isolated from adipose‐derived stromal cells in cardiomyocytes in ischemic conditions, to promote cardiac protection.^[^ ^53^ ^]^ Preincubation of cardiomyocytes with chlorpromazine in both hypoxic and normal oxygen conditions lowered EV uptake and subsequent EV‐mediated miR‐214 delivery, a miRNA known to be involved in cardiac protection. Furthermore, in mice treated with EVs in combination with chlorpromazine, following coronary artery ligation, EVs failed to promote protection against hypoxia damage, whereas EVs alone could.^[^ ^53^ ^]^ As such, clathrin‐mediated internalization is deemed crucial for the uptake of these EVs in cardiomyocytes to offer protection against hypoxic damage. Chlorpromazine treatment also almost completely inhibited the uptake of normal and preeclamptic syncytiotrophoblasts‐derived EVs in HCAECs.^[^ ^54^ ^]^ In the absence of pharmacological inhibitors, the internalization of normal syncytiotrophoblasts‐derived EVs resulted in the downregulation of intercellular adhesion molecule 1 (ICAM‐1) expression, as opposed to preeclamptic EVs, which had no effect. High levels of ICAM‐1 expression are indicative for HCAEC activation, an important signal for inflammation. Therefore, the downregulation suggests a protective role of normal EVs, which appears to be absent in those isolated from preeclamptic placenta.^[^ ^54^ ^]^ This, however, is in contrast with another study that reported an upregulation of ICAM‐1 after uptake of preeclampsia‐derived EVs by human dermal microvascular endothelial cell‐1 (HMEC‐1).^[^ ^55^ ^]^ The difference in ICAM‐1 levels may be due to the variation in recipient cell types, but may also be a result of a different internalization route. Another example of clathrin‐mediated internalization of EVs was reported by Tian and co‐workers. The authors used EVs derived from rat pheochromocytoma PC‐12 cells, which after chlorpromazine treatment were shown to be partly internalized through clathrin‐mediated pathways in bone marrow stromal cells (BMSCs).^[^ ^56^ ^]^ After uptake, transforming growth factor β receptor II and tropomyosin‐1 expression was downregulated, which may be mediated by the transfer of miR‐21. Expression of these factors plays a vital role in tumor mutation and progression.^[^ ^56^ ^]^ Overall, these results indicate that clathrin‐mediated uptake of EVs is involved in mediating gene transcription in a variety of cell lines, but also with a variety of EV types.

Taken together, these results suggest that clathrin‐mediated internalization is an important endocytosis mechanism for the uptake of drug delivery vesicles. This pathway does not seem to be preferentially exploited by either natural or synthetic vesicles to gain entry in cells. Although this pathway has been associated with a high likelihood of lysosomal degradation, both liposomes and EVs exhibited successful nucleic acid delivery. Furthermore, clathrin‐dependent endocytosis of EVs is deemed pivotal for their inherent therapeutic utility, such as cardiac protection against hypoxia damage, as well as regulation of gene expression that is essential for epithelial cell activation and tumor suppression.

#### 2.1.2 Caveolae‐Dependent Endocytosis

Caveolae‐mediated internalization, depicted in **Figure** **3**, proceeds through “flask” shaped invaginations of the plasma membrane, known as caveolae. At the site of this particular invagination, membrane domains are rich in glycolipids, sphingolipids, and cholesterol (see Figure 3‐1).^[^ ^47^, ^57^ ^]^ The main proteins that orchestrate and stabilize caveolae‐mediated endocytosis are the caveolin and cavin family, as well as the dynamin‐related EH Domain Containing 2 ATPase and the BAR protein domain‐containing syndapin/Pacsin2.^[^ ^58^ ^]^ Caveolins are hairpin‐like integral membrane proteins, anchored to the plasma membrane at the cytosolic side, which bind to actin filaments, to ensure caveolae formation.^[^ ^13^, ^47^, ^59^, ^60^ ^]^ Once the invagination is initiated, cavins are recruited from the cytosol, which oligomerizes into trimers, thus promoting membrane remodeling and generating “flask”‐shaped invaginations (see Figure 3‐2).^[^ ^61^ ^]^ Following oligomerization, the EH Domain Containing 2 ATPase is recruited to the site of the invagination for stabilization.^[^ ^58^, ^62^ ^]^ Assisted by the GTPase activity of dynamin, the membrane is then pinched off, generating endocytic vesicles called caveosomes, which are then free to traffic intracellularly (see Figure 3‐3,4).^[^ ^57^, ^63^ ^]^ Predominantly in epithelial cells, caveosomes can undergo transcytosis, which consists of transport across the cell, fusing with the plasma membrane, and releasing the cargo in the extracellular space again, on the opposite side of entry. However, in the majority of cell types, caveosomes enter the endo‐lysosomal pathway and are subjected to degradation. Alternatively, caveosomes can circumvent degradation by transporting their cargo to organelles, such as the endoplasmic reticulum or mitochondria.^[^ ^47^ ^]^ It is believed that many pathogens prefer caveolae‐mediated entry to overcome lysosomal degradation.^[^ ^13^, ^38^, ^47^, ^64^, ^65^, ^66^ ^]^ In this section, we will examine the involvement of caveolae‐mediated endocytosis in the uptake of liposomes and EVs, also summarized in Table S3 (Supporting Information). Furthermore, we address the implications of caveolae‐associated pathways in relation to cargo delivery and describe the possible outcomes.

To assess caveolae‐associated internalization, a variety of pharmacological inhibitors have been employed to target cholesterol, thereby distorting the formation of caveolae and subsequently hindering uptake. Nystatin, filipin, and methyl‐β‐cyclodextrin are the most commonly used inhibitors that sequestrate cholesterol from the plasma membrane and destabilize caveolae formation.^[^ ^28^ ^]^ Other inhibitors, such as genistein and nocodazole, interfere with caveolae‐mediated endocytosis by locally disrupting the actin or microtubule network, respectively.^[^ ^67^, ^68^, ^69^ ^]^ A different inhibitory process can be achieved using indomethacin treatment. This inhibitor blocks the internalization and recycling of folate receptors. These have been found to co‐localize with caveolae, consequently inhibiting internalization.^[^ ^70^, ^71^ ^]^ However, the specificity of these markers remains a topic of debate, as they may interfere with other internalization mechanisms with similar factors at play. One such example is dynasore, a pharmacological inhibitor that interferes with dynamin, whose role is recognized in clathrin‐ and caveolae‐mediated endocytosis, as well as phagocytosis.^[^ ^37^, ^57^, ^72^, ^73^ ^]^ An alternative to inhibitors is co‐localization analysis with well‐established caveolae‐associated markers, such as cholera toxin subunit B.^[^ ^32^ ^]^

To date, several studies have reported the internalization of liposomes through caveolae‐associated pathways.^[^ ^68^, ^74^, ^75^, ^76^ ^]^ For instance, it was established that HUVECs and monkey kidney carcinoma COS‐7 cells take up DOPE:CHEMS (3:2 molar ratio) liposomes predominantly in a caveolae‐dependent manner. The internalization of liposomes was blocked in both cell types following treatment with methyl‐β‐cyclodextrin and filipin. Caveolae‐mediated endocytosis was also confirmed by co‐localization analysis using cholera toxin subunit B.^[^ ^74^ ^]^ Ruyra et al. showed that methyl‐β‐cyclodextrin treatment of zebrafish hepatocytes and trout macrophages reduced internalization efficiency of DLPC:cholesteryl:cholesterol:PEG (50:10:35:5 mol%) liposomes by 60% and 31%, respectively, suggesting caveolae‐mediated uptake.^[^ ^75^ ^]^ The delivery of both lipopolysaccharide and a synthetic analog of viral dsRNA together via liposomes stimulated an antiviral response in fish species.^[^ ^75^ ^]^ Another lipid formulation that was shown to internalize through caveolae‐associated pathways is DOPC:DOPG:MPB‐PE (40:10:50 molar ratio) in HeLa cells. Labeled with DiD, these liposomes showed a more than twofold increase in co‐localization with immunostained caveolin‐1 than with clathrin proteins, suggesting a predominant caveolae‐mediated internalization.^[^ ^76^ ^]^ Nonetheless, as for clathrin‐mediated mechanisms, it is important to note that the transfection efficiencies of drug delivery vesicles in vitro, could be affected by culture conditions, such as the presence or absence of serum in the growth medium.^[^ ^77^ ^]^ As such, Bae et al. studied the effect of serum on the luciferase transfection efficiency of DOPE:cholesterol lipoplexes in COS‐7 cells.^[^ ^78^ ^]^ Filipin and genistein treatment significantly decreased expression in the presence of serum, reducing luciferase activity by 15%, compared to a lack of effect in the absence of serum. Likewise, incubation with methyl‐β‐cyclodextrin decreased transfection efficiency by approximately 80% in the presence of serum, compared to no significant effect in serum‐free medium.^[^ ^78^ ^]^ These findings suggest that while caveolae‐mediated internalization of liposomes plays an important role in uptake, as well as efficient transfection in various cell lines, experimental details, such as serum supplementation can contribute significantly to biological outcomes.

So far, we discussed the contribution of caveolae‐mediated endocytosis in liposome internalization and subsequent gene expression. However, certain studies reported that liposome uptake and caveolae expression could negatively influence one another, which in turn may affect liposome uptake. Inoh et al. showed that internalization of DOPE:OH‐cholesterol (20:30 molar ratio) liposomes in RBL‐2H3 mast cells exerts a negative effect on the translocation of caveolin‐1 from the cytosol to the plasma membrane. This effect interferes with PI3K activity, which in turn may influence caveolae‐mediated internalization.^[^ ^79^ ^]^ Following antigen stimulation of mast cells, caveolin‐1 was translocated from the cytosol to the plasma membrane, ultimately colocalizing with a subunit of PI3K, termed p85. The colocalization was no longer present after liposome uptake, suggesting that these nanocarriers suppressed translocation of caveolin‐1, affecting actin activity.^[^ ^79^ ^]^ This suppressed the activation of mast cells, whose role in inflammatory responses are a key area of research.^[^ ^80^ ^]^ Although liposome uptake can interfere with caveolin‐1 translocation, it appears that caveolae too can impact the therapeutic efficacy of these nanocarriers. siRNA‐mediated knockdown of caveolae and treatment with filipin and nystatin increased the transfection efficiency of amide:DOPE (1:1 molar ratio) lipoplexes by three to fourfold in liver sinusoidal endothelial Sk‐hep1 cells, compared to untreated controls.^[^ ^81^ ^]^ Further examination showed that after inhibition of clathrin‐mediated endocytosis and macropinocytosis, the transfection efficiencies of lipoplexes decreased significantly.^[^ ^81^ ^]^ In general, these data suggest that in certain cases caveolae‐mediated uptake of these lipoplexes leads to poor therapeutic efficacy and, upon inhibition of this pathway, compensatory mechanisms may appear, which would be favorable for gene therapy.

As described above, caveolae‐mediated internalization serves as an important entry route for liposomes, but has also been associated with the uptake of EVs, as we will describe below. Caveolae‐mediated uptake was reported to be important for EVs of various cellular origins.^[^ ^68^, ^82^, ^83^ ^]^ For example, the internalization of EVs derived from Epstein‐Barr Virus‐infected B cells by nasopharyngeal carcinoma cells was dependent on caveolae‐associated pathways.^[^ ^84^ ^]^ Dynasore treatment of these cells almost completely diminished the uptake of both virus infected and noninfected cell‐derived DiI‐labeled EVs. Furthermore, siRNA‐mediated downregulation of caveolin‐1 had similar effects as dynasore treatment, indicating caveolin‐dependency of EV uptake in these cells. Following internalization of EVs isolated from virus‐infected B cells of a particular latency, i.e., type III, it was shown that latent membrane protein 1 was transferred to recipient cells which in turn enhanced cell proliferation rate and upregulated ICAM‐1 expression.^[^ ^84^ ^]^ Caveolae‐mediated endocytosis was also shown to be present in the case of *Trichomonas vaginalis*‐derived EVs in Benign Prostatic Hyperplasia‐1 (BPH‐1) cells.^[^ ^82^ ^]^ Genistein, filipin, and methyl‐β‐cyclodextrin treatment reduced uptake by approximately 80–90%. Supplementing these cells with cholesterol during the methyl‐β‐cyclodextrin treatment led to the uptake of EVs increasing by 70%, underlining the role of caveolae‐mediated uptake. Furthermore, overexpressing caveolin‐1 in HEK293 cells increased *T. vaginalis*‐derived EV uptake by ≈75%, compared to wild‐type HEK293 cells that naturally express very low levels of caveolin‐1. This suggests an active role of caveolin‐1 in the uptake of EVs, though this will depend on the recipient cell type.^[^ ^56^ ^]^ Furthermore, non‐engineered HEK293 cells took up 80% less EVs than BPH‐1 and wild‐type Chinese hamster ovary K1 (CHO‐K1), in the absence of pharmacological inhibitors, indicating caveolae‐dependent endocytosis may be involved.^[^ ^82^ ^]^

Interestingly, certain cell types have the potential to regulate their caveolin‐1 levels to control uptake. A recent study by Yue and co‐workers indicated that oxygen‐ and glucose‐deprived neurons upregulate caveolin‐1 expression, thereby stimulating uptake of HUVEC‐derived EVs.^[^ ^83^ ^]^ These EVs were found to be enriched in miR‐1290 content, which is important for neuronal protection against damage induced by oxygen‐glucose deprivation. Neuronal cells exposed to ischemia‐reperfusion overexpressed caveolin‐1, which in turn stimulated miR‐1290 transfer through EV uptake and attenuated apoptosis of primary neurons.^[^ ^83^ ^]^ Conversely, natural expression of caveolin‐1 reduced uptake of glioblastoma cell‐derived EVs in various cell lines, such as Uppsala 87 Malignant Glioma (U87‐MG), CHO‐K1, and HeLa cells. This was evidenced by the increased uptake of EVs in caveolin‐1 knockout mouse embryonic fibroblasts cells, compared to controls. Importantly, this suggests that caveolin‐1 in certain recipient cell types can negatively regulate internalization.^[^ ^85^ ^]^ Taken together, these findings suggest that the expression of caveolin‐1 is able to mediate the uptake of EVs, which possibly influences the exerted biological response. However, more data is needed to establish the precise role of caveolae‐mediated uptake of EVs and its implications in therapeutic efficacy, since it appears to depend heavily on recipient cell type.

To establish similarities and differences in uptake and the subsequent biological outcomes between liposomes and EVs, it is crucial to perform head‐to‐head comparisons. Saux et al. compared the internalization mechanisms of murine mesenchymal stromal cells (MSCs)‐derived EVs with HSPC:cholesterol (56:39 mol:mol) liposomes, the standard Doxil composition, in MSCs and mouse fibroblasts (NIH3T3 cells).^[^ ^68^ ^]^ While EVs were internalized mainly via caveolae‐mediated pathways in a cholesterol‐dependent manner, HSPC:cholesterol liposomes were partly taken up through caveolae‐associated pathways, but independent of cholesterol, as shown by genistein and methyl‐β‐cyclodextrin treatment. For uptake analysis, liposomes and EVs were fluorescently labeled with DiI and although the number of particles was constant, EVs were internalized to a greater extent than liposomes (more than twofold). Additionally, cholesterol depletion using methyl‐β‐cyclodextrin improved the uptake efficiency of liposomes almost twofold, compared to those of EVs which were reduced.^[^ ^68^ ^]^ Overall, EVs appear to be more favorable for drug delivery purposes in MSCs and NIH3T3 cells, as these nanocarriers yielded higher cellular internalization than the HSPC:cholesterol liposomes. Nonetheless, the therapeutic relevance of such differences in cellular uptake remains to be investigated and is likely to be different for different therapeutic targets.

Recently, to improve the systemic circulation of lipid nanoparticles and enhance their cellular internalization a new type of drug delivery system has been suggested: EV‐inspired liposomal formulations, or so‐called EV‐mimicking liposomes. Lu and co‐workers synthesized EV‐mimicking lipoplexes using a DOPC:SM:cholesterol:DOPS:DOPE (21:17.5:30:14:17.5 molar ratio) lipid formulation and assessed stability, uptake mechanisms, encapsulation, and transfection efficiency.^[^ ^86^ ^]^ The authors found, based on transfection efficiency, that the internalization mechanism of EV‐mimicking liposomes was cell type‐dependent. For instance, uptake in A549 cells occurred via caveolae‐mediated endocytosis, macropinocytosis, and membrane fusion. However, macropinocytosis appears to be the dominating uptake mechanism of EV‐mimicking lipoplexes in A549 cells. On the other hand, in HUVECs, caveolae‐mediated endocytosis was found to prevail for the EV‐mimicking lipoplexes. Uptake of other lipoplex formulations such as Lipofectamine 2000 and DOTAP:DOPC:cholesterol (40:40:20 molar ratio) was found to depend on both clathrin‐ and caveolae‐mediated pathways, but not on macropinocytosis, suggesting uptake to be formulation‐dependent. EV‐mimicking lipoplexes exhibited threefold higher cellular uptake and transfection efficiency than those of DOPC:cholesterol (70:30 molar ratio). Yet, Lipofectamine 2000 and DOTAP:DOPC:cholesterol formulations were most effective, in terms of internalization and transfection efficiency of all formulations tested.^[^ ^86^ ^]^ Importantly, it is known that different liposomal formulations exhibit significant variations in their physical properties, such as surface charge.^[^ ^4^ ^]^ For example, the zeta potential of the EV‐mimicking, the DOTAP:DOPC:cholesterol, and the DOPC:cholesterol lipoplexes measured in PBS were −23.77 ± 2.12 mV, +34.9 ± 4.15 mV, and −3.9 ± 0.39 mV, respectively. Conversely, the size of these nanocarriers was relatively similar, ranging from 116 to 129 nm. As such, the difference in surface charge may be a contributing factor to the observed differences in internalization pathways of these nanocarriers.^[^ ^86^ ^]^ This study further highlights the importance of lipid composition and target cells for head‐to‐head comparison studies between natural and synthetic drug delivery systems and preferential uptake mechanisms, which present great differences in the identified pathways. It remains challenging to attribute therapeutic potential to one specific uptake pathway without assessing the influence of pharmacological inhibitors used.

Overall, similar to clathrin‐dependent uptake, caveolae‐mediated endocytosis is a key mechanism contributing to the delivery of liposomes and EVs. However, it has been shown that, in certain cell types, caveolin expression and the type of liposomes used can negatively affect one another. In this regard, the uptake of certain types of EVs was also shown to be negatively impacted by the presence of caveolins. However, the role of lipid composition and recipient cell types in such situations remains to be explored to unlock the full potential of natural and artificial drug delivery systems. Therefore, it is imperative to conduct head‐to‐head comparisons to accurately evaluate liposomes and EVs for therapeutic applications.

#### 2.1.3 Clathrin‐ and Caveolae‐Independent Endocytosis

A distinct class of internalization mechanisms is referred to as clathrin‐ and caveolae‐independent endocytosis, which internalize a variety of cargo, without the involvement of clathrin and caveolin.^[^ ^87^ ^]^ Similar to caveolae‐dependent uptake, this class of endocytosis involves lipid raft domains and encompasses several endocytic routes that are mediated by specific GTPases, namely ADP‐ribosylation factor 6 (Arf6), cell division control protein 42 homolog (Cdc42), and Ras homolog family member A (RhoA).^[^ ^88^, ^89^ ^]^ Arf6‐mediated endocytosis is responsible for the internalization of proteins involved in nutrient transport, matrix interaction, and immune function.^[^ ^89^ ^]^ This mechanism also aids in recycling, actin remodeling, regulation of macropinocytosis, and endosome dynamics.^[^ ^19^, ^89^, ^90^ ^]^ Uptake via this mechanism is independent of dynamin.^[^ ^91^ ^]^ Similarly to Arf6‐dependent uptake, Cdc42‐dependent endocytosis is also independent of dynamin and it is also involved in macropinocytosis, as a regulator of actin polymerization.^[^ ^89^, ^90^ ^]^ The membrane scission of dynamin‐independent endocytic pathways are mediated by actin dynamics.^[^ ^92^ ^]^ In contrast to the Arf6‐ and Cdc42‐pathways, RhoA‐dependent endocytosis depends on dynamin and is responsible for the internalization of β‐chain of the interleukin‐2 receptor and for the regulation of actin cytoskeleton dynamics.^[^ ^90^, ^91^ ^]^ Some of the molecules mediating RhoA‐dependent endocytosis are Rac1, P21 (RAC1) Activated Kinase 1 (PAK1), and PI3K.^[^ ^90^ ^]^ Another clathrin‐ and caveolae‐independent endocytic route is mediated by flotillin, which shares certain characteristics with caveolae‐regulated pathways, including protein topology, morphology of the endocytic pit, and regulation by Src‐family kinases. Furthermore, flotillins have a role in phagocytosis, mediation of the actin cytoskeleton, and polarization of hematopoietic cells among others.^[^ ^66^ ^]^ A few studies have reported uptake of EVs through clathrin‐ and caveolae‐independent endocytosis, as will be addressed in this section. However, to our knowledge, there are no studies that reported liposome uptake associated with these pathways.

The number of studies that focused on clathrin‐ and caveolae‐independent uptake of EVs remains limited and often these mechanisms act alongside other endocytosis mechanisms. For instance, Verdera and co‐workers showed that along caveolae‐mediated internalization and macropinocytosis, A431‐derived EVs also enter HeLa cells independently of clathrin and caveolin. Upon siRNA‐mediated knockdown of flotillin‐1, RhoA, Rac1, and PAK1, cellular uptake of EVs decreased significantly.^[^ ^93^ ^]^ Similarly, BMSC‐derived EVs were shown to be predominantly internalized in RPMI 8226 and U266 B lymphocytes, via classical endocytosis mechanisms, but also enter cells via flotillin‐mediated pathways to some extent. shRNA‐mediated knockdown of flotillin‐1 inhibited the uptake of EVs by 20% and 40% in RPMI 8226 and U266 B lymphocytes, respectively.^[^ ^94^ ^]^ Also *Porphyromonas gingivalis*‐derived EVs were reported to enter HeLa cells using clathrin‐, caveolae‐, and dynamin‐independent pathways.^[^ ^95^ ^]^ The authors showed that transfected cells expressing a dominant‐negative form of Rac1, induced a strong inhibition of EV uptake, whereas Cdc42‐, dynamin‐, and epidermal growth factor receptor pathway substrate 15‐dominant‐negative cells had no effect. These findings suggest that *P. gingivalis*‐derived EVs are internalized in HeLa cells through a Rac1‐regulated pathway. This was further substantiated by the reduction of EV uptake upon wortmannin treatment, which affects PI3K, required for the activation of RhoA and its downstream target Rac1. Furthermore, cholesterol depletion via methyl‐β‐cyclodextrin treatment also interfered with EV uptake, whereas co‐localization analysis with caveolin showed no significant results. Together, these findings suggest that *P. gingivalis*‐derived EVs enter HeLa cells via lipid raft‐mediated pathways.^[^ ^95^ ^]^ However, Rac1 and PI3K are also known for their role in macropinocytosis, which cannot be excluded as a potential entry route, based on the data provided.^[^ ^96^, ^97^ ^]^

Interestingly, the contents of EVs may also stimulate clathrin‐ and caveolae‐independent endocytosis, thereby potentiating their own uptake. Mayor et al. demonstrated that EVs derived from oligodeoxynucleotide‐stimulated macrophages contain elevated levels of Cdc42, as compared to those isolated from non‐treated cells.^[^ ^98^ ^]^ Uptake of EVs, isolated from stimulated RAW264.7 cells, induced TNF‐α release in native macrophages, which in turn activated cellular Cdc42 and potentiated EV uptake.^[^ ^98^ ^]^ Furthermore, Morad and co‐workers reported that astrocytes internalized MDA‐MB‐231 breast cancer cell‐derived EVs only through Cdc42‐dependent pathways.^[^ ^99^ ^]^ None of the classical endocytosis inhibitors, such as chlorpromazine, filipin, and 5‐(n‐ethyl‐n‐isopropyl)‐amiloride (EIPA) exerted a significant effect on internalization, as compared to ML141, a Cdc42/Rac1 inhibitor. Subsequent proteomics and co‐localization analysis further revealed that MDA‐MB‐231 cell‐derived EVs express surface proteins with an affinity toward Cdc42‐dependent endocytosis, indicating that internalization of these EVs occurs via Cdc42‐regulated pathways.^[^ ^99^ ^]^

We have not come across studies presenting clathrin‐ and caveolae‐independent endocytosis of liposomes. However, EVs do exploit these mechanisms to gain entry into cells and more insight is needed to elucidate their potential contribution to therapeutic effects. Based on the results from EVs, it holds promising opportunities to exploit these routes to gain access or even enhance entry into cells and studies should be performed with liposomes in relation to these uptake mechanisms to unlock their full potential.

#### 2.1.4 Macropinocytosis

Macropinocytosis, depicted in **Figure** **4**, is an entry route that is mainly regulated by growth factor signaling through the activation of tyrosine kinases and it is associated with cell motility.^[^ ^25^ ^]^ This pathway relies on a signaling cascade involving the Ras superfamily of GTPases, including PI3K, Rac1, Cdc42, and Arf6 (see Figure 4‐1).^[^ ^24^, ^100^, ^101^ ^]^ The cascade controls the actin cytoskeleton, which leads to the formation of membrane ruffles that can engulf large cargos from the adjacent extracellular space (see Figure 4‐2).^[^ ^20^, ^97^, ^100^ ^]^ Furthermore, the actin cytoskeleton ensures proper vesicle trafficking. Other factors involved in macropinocytosis are PAK1, which guarantees proper closure of macropinosomes, endocytic vesicles formed upon internalization via macropinocytosis.^[^ ^37^ ^]^ As dynamin inhibition interferes with the targeting of Rac1 to membrane ruffles, this GTPase may also play a role in the proper enclosure of macropinosomes (see Figure 4‐3).^[^ ^96^ ^]^ In general, macropinosomes can be recycled back to the plasma membrane, releasing the cargo into the extracellular space, or continue their journey to lysosomes (see Figure 4‐4).^[^ ^25^, ^102^ ^]^ Macropinocytosis can engulf larger nanoparticles than the endocytosis mechanisms discussed so far, resulting in endocytic vesicles that can carry a bigger cargo given their size (≈0.5–1 µm).^[^ ^20^, ^25^, ^87^, ^103^ ^]^ In particular in cancer cells, macropinocytosis was shown to regulate nutrient uptake, thus contributing to cell proliferation. Therefore, targeting drug delivery systems for macropinocytotic uptake may represent an approach to enhance accumulation of nanocarriers in cancerous tissues.^[^ ^102^ ^]^ This pathway is also exploited by pathogens, such as viruses and bacteria.^[^ ^25^, ^96^ ^]^ While many natural and artificial nanocarriers are internalized through clathrin‐ and caveolae‐associated pathways, in the following section we address how macropinocytosis contributes to the uptake of liposomes and EVs, and how this mechanism could be exploited for therapeutic applications. A summary of these findings can also be found in Table S4 (Supporting Information).

As macropinocytosis heavily relies on actin reorganization, the majority of inhibitors used to identify this endocytosis mechanism are designed to interfere with PI3K activity. Such inhibitors interfering with membrane ruffling are wortmannin, LY294002, cytochalasin D, and colchicine.^[^ ^45^, ^96^, ^104^ ^]^ Amiloride‐based inhibitors, e.g., EIPA, inhibit macropinocytosis by interfering with the Na^+^/H^+^ exchanger located in plasma membranes, which has downstream effects on pH, Rac1 activation, and Cdc42 signaling.^[^ ^32^, ^105^ ^]^ Although cholesterol depletion is not widely accepted to identify macropinocytosis, it should be taken into account, as this technique does not identify solely caveolae‐mediated internalization, but may affect macropinocytosis as well.^[^ ^106^, ^107^ ^]^ Furthermore, macropinocytotic uptake can be corroborated through co‐localization analysis with dextran, a well‐established endocytosis marker for this mechanism.^[^ ^108^, ^109^ ^]^

Several studies have demonstrated the internalization of liposomes and lipoplexes through macropinocytosis using established markers or pharmacological inhibitors. For example, Pozzi et al. observed that DOTAP:DOPC and DOPE:DC‐cholesterol (molar ratios not mentioned)‐based lipoplexes, containing Cy3‐labeled DNA, co‐localized with FITC‐dextran in CHO‐K1 cells.^[^ ^110^ ^]^ In comparison, the same lipid formulations loaded with protamine pre‐condensed DNA, co‐localized with both dextran and Alexa Fluor 488‐labeled transferrin, a clathrin endocytic marker. The additional clathrin‐mediated endocytosis in case of lipoplexes carrying protamine‐complexed DNA may be accounted for by differences in vesicle size, as these formulations are smaller than those containing bare DNA. Furthermore, based on the quantification of internalized lipoplexes, bare DNA‐containing nanoparticles had higher internalization potency than those containing DNA complexed with protamine. Together, these data indicate that macropinocytosis alone has a higher cellular uptake efficiency than when additional uptake mechanisms are present, such as clathrin‐mediated endocytosis. However, the efficiency of lipoplexes to transfect cells for luciferase expression was found to depend on lipid formulation rather than internalization mechanism, since DOPE:DC‐cholesterol lipoplexes containing bare or protamine‐complexed DNA exhibited higher transfection efficiencies than DOTAP:DOPC lipoplexes.^[^ ^110^ ^]^ This is presumably attributed to the fusogenic abilities of DOPE with the endosomal membrane, therefore promoting transfection.^[^ ^77^ ^]^ Thus, macropinocytotic uptake of different liposomal formulations yields different cellular internalization. Nonetheless, the transfection efficiency of nanocarriers seems to depend on both the lipid composition and to some extent, the route of uptake. In this regard, DOPE:DC‐cholesterol lipoplexes seem to be more promising.

Alongside liposomal formulation, the physical characteristics of liposomes are an important factor that may affect the internalization route. Notably, surface charge has been analyzed in this respect. Montizaan et al. studied the effect of zwitterionic modifications on uptake mechanisms of liposomes in HeLa cells.^[^ ^111^ ^]^ The authors used formulations of anionic DOPG and zwitterionic DOPC mixed with cholesterol in a 2:1 molar ratio. Both displayed a low polydispersity index with a diameter of ≈100 nm. The zeta potential measured in PBS was −40 ± 3 mV for DOPG:cholesterol and close to neutral at −1 ± 1 mV for DOPC:cholesterol. This difference in charge was shown to affect cellular uptake in serum‐free conditions. The DOPG:cholesterol liposomes reached higher uptake levels in the course of 5 h, compared to DOPC:cholesterol liposomes. Uptake mechanisms in these conditions were not explored further. However, incubating the liposomes in cell culture medium containing human serum, significantly altered the zeta potential: DOPG:cholesterol liposomes became more neutral (−8 ± 1 mV) and DOPC:cholesterol liposomes showed similar values (−6 ± 1 mV). DOPG:cholesterol liposomes were shown to adsorb more protein on their surface than DOPC:cholesterol and also this protein corona had a different composition. Similar to serum‐free conditions, a higher uptake for DOPG:cholesterol was seen. Still, uptake kinetics in these conditions differed strongly, indicating that the original charge may be less relevant, but that the composition of the protein corona is a decisive factor. Pharmacological inhibitors and siRNA silencing were used to block several key endocytic pathways. EIPA treatment reduced uptake: DOPC:cholesterol by 60% after 7 h and DOPG:cholesterol by 75% after 1 h. This implicates macropinocytosis in both formulations, be it to a different extent and with different kinetics. In contrast, treatment with cytochalasin D and nocodazole to block polymerization of F‐actin and microtubules, respectively, reduced uptake of the DOPG:cholesterol formulation (80% and 50%, respectively) far more than DOPC:cholesterol (<30% and 30% maximum, respectively). Treatment with chlorpromazine was shown to reduce the uptake of DOPG:cholesterol by 55% over time, but had no effect on DOPC:cholesterol. This indicates involvement of clathrin‐mediated endocytosis in DOPG:cholesterol uptake. Overall, it would appear that the internalization of both formulations is dominated by different pathways, even if their surface charge is similar.^[^ ^111^ ^]^

For other liposomal formulations, Cardarelli and co‐workers investigated the endocytosis mechanisms using luciferase expression to examine the internalization routes of DOTAP:DOPC (1:1 molar ratio) and DOPE:DC‐cholesterol (1:1 molar ratio) lipoplexes in CHO‐K1 upon endocytosis inhibition.^[^ ^112^ ^]^ Macropinocytosis inhibition using wortmannin treatment reduced the transfection efficiency of DOTAP:DOPC and DOPE:DC‐cholesterol lipoplexes to 10% and 90%, respectively. Meanwhile, cholesterol perturbation, by means of methyl‐β‐cyclodextrin treatment, reduced the transfection efficiency of DOTAP:DOPC formulations to 50% and those of DOPE:DC‐cholesterol to 5%. Although methyl‐β‐cyclodextrin is often used to block caveolae‐mediated internalization, other caveolae‐interfering inhibitors, i.e., genistein, had no effect on luciferase protein expression. These findings suggest that macropinocytosis is sensitive to cholesterol depletion and the internalization efficiency is dependent on lipid composition. As such, the DOTAP:DOPC lipoplexes were taken up primarily by fluid‐phase macropinocytosis, while those consisting of DOPE:DC‐cholesterol were primarily engulfed in a cholesterol‐dependent manner, entering through the tight clustering of sphingolipids and cholesterol.^[^ ^112^ ^]^ Although clathrin‐mediated endocytosis is thought to only have a minor, if any, contribution to the internalization of DOPE:DC‐cholesterol (1:1 molar ratio) lipoplexes in CHO‐K1 cells, chlorpromazine treatment reduced the uptake of the same liposome formulation to ≈66% in A549 cells, indicating clathrin‐mediated endocytosis and dependence on recipient cell type.^[^ ^52^ ^]^ Moreso, luciferase knockdown in luciferase‐transfected A549 cells by targeted lipoplexes was diminished to approximately 5% after chlorpromazine treatment, suggesting successful transfection efficiency following clathrin‐mediated uptake (as also discussed in section 2.1.1.). Additionally, methyl‐β‐cyclodextrin and EIPA treatment of the cells reduced the uptake of siRNA loaded liposomes by ≈45–60%, compared to control, while siRNA target interaction and luciferase activity were reduced to a greater extent by ≈54–57% and ≈89–98%, respectively.^[^ ^52^ ^]^ This study further supports the cholesterol sensitivity of macropinocytosis.

Cell type dependency of macropinocytosis was also evidenced in the case of DOPE:CHEMS (3:2 molar ratio) liposomes. HUVECs displayed mainly macropinocytotic uptake of these liposomes, among other endocytosis mechanisms, yet in COS‐7 cells it was absent.^[^ ^74^ ^]^ These results indicate that the uptake and gene transfection potency of DOPE:CHEMS lipoplexes could depend on the recipient cell type, as well as on the contribution of additional uptake mechanisms. Furthermore, macropinocytosis has been deemed crucial for successful transfection of CHO‐K1 cells by charge‐reversal amphiphile lipoplexes containing GFP‐ and β‐galactosidase‐encoding DNA.^[^ ^113^ ^]^ Amiloride and wortmannin treatment reduced uptake by 44% and 78%, respectively, and lowered transfection efficiency by 95%. This suggests that macropinocytosis of charge‐reversal amphiphile lipoplexes is a major contributor for successful gene transfection, which may be due to the inherent leakiness of macropinosomes. Although genistein treatment had no interference with uptake or gene transfection, methyl‐β‐cyclodextrin treatment reduced both processes by 80–85%, further substantiating the cholesterol sensitivity of macropinocytosis.^[^ ^113^ ^]^ Overall, macropinocytosis plays an important role both in uptake and transfection of liposomal formulations. Nonetheless, it seems that this process is dependent on cell type and lipid composition. Importantly, to date, the role of cholesterol in macropinocytosis is not fully elucidated, and appropriate controls are needed to distinguish between caveolae‐mediated endocytosis and macropinocytosis upon cholesterol depletion.

EVs have also been reported to exploit macropinocytosis to gain entry in several cell types. Oligodendrocyte‐secreted EVs are efficiently taken up through macropinocytosis in microglial EOC‐20 cells, predominantly in a subpopulation of major histocompatibility complex (MHC)‐class‐II‐negative cells in vitro and in vivo.^[^ ^114^ ^]^ Several treatments with pharmacological inhibitors, such as dynasore, NSC23766 (a Rac1 inhibitor), cytochalasin D, and amiloride reduced the uptake of PKH67‐labeled EVs by about half. Furthermore, cells co‐incubated with EVs and liposomes made from a mixture of phosphatidylcholine and phosphatidylserine exhibited a twofold lower EV uptake, compared to the co‐incubation with liposomes containing only phosphatidylcholine. Thereby, together with the pharmacological inhibition analysis, the reduction in EV uptake is thought to be due to the competitive recognition of phosphatidylserine by microglia cells. It is hypothesized that lipid nanoparticles with high phosphatidylserine content may have an affinity for macropinocytotic uptake.^[^ ^114^ ^]^ Furthermore, macropinocytosis of EVs also appeared in cancer cells expressing K‐Ras mutations through which their cellular internalization was enhanced. Human pancreas carcinoma‐derived MIA PaCa‐2 cells expressing oncogenic K‐Ras, led to 14‐fold higher uptake of HeLa cell‐isolated EVs, compared to BxPC‐3 cells expressing wild‐type K‐Ras.^[^ ^115^ ^]^ Kamerkar and co‐workers further corroborated the enhanced macropinocytotic uptake of EVs in pancreatic cancer cells expressing oncogenic K‐Ras, compared to BxPC‐3 cells expressing wild‐type K‐Ras.^[^ ^116^ ^]^ Confocal images of PANC‐1 cells expressing oncogenic K‐Ras^G12D^ incubated with PKH67‐labeled EVs isolated from fibroblast‐like mesenchymal cells showed a significantly larger uptake than BxPC‐3 cells expressing wild‐type K‐Ras. In contrast to the large amount of internalized EVs, the signal of PKH67‐labeled liposomes (lipid composition not mentioned) presented less fluorescence signal, suggesting reduced cellular uptake efficiency, compared to EVs. Furthermore, EIPA treatment reduced EV uptake in a concentration‐dependent manner, while that of liposomes remained unaffected. Additionally, proteinase K treatment completely diminished the signal of AF647‐labeled siRNA delivered by EVs into PANC‐1 cells, as opposed to those delivered by liposomes. Together, these findings suggest that the enhanced cellular uptake of EVs is due to the synergistic effect of the stimulated macropinocytosis by oncogenic K‐Ras mutations and the inherent membrane surface constituents of natural drug delivery systems.^[^ ^116^ ^]^ In the same study, in vivo analysis demonstrated high affinity of PKH67‐labeled EVs for pancreatic accumulation, since the pancreas was the third organ containing the largest amount of EVs, after the liver and lung. Moreover, pancreatic cells expressed almost threefold higher accumulation of AF647‐labeled siRNA targeting K‐Ras^G12D^ upon EV delivery, compared to liposomes. Therefore, the cellular uptake and targeting efficiency of siRNA‐encapsulated EVs ensured pancreatic cancer suppression and an extended survival rate in multiple mouse models.^[^ ^116^ ^]^ Overall, macropinocytosis is a highly exploited pathway for EV uptake, especially in cancerous cell lines containing K‐Ras mutations.

Macropinocytotic uptake of liposomes can be induced by clathrin‐mediated uptake of these nanoparticles. This was evidenced in a study conducted by Gilleron and co‐workers in which DLin‐MC3‐DMA:DSPC:cholesterol:DMG‐PEG (≈50:10:38.5:1.5 molar ratio) lipoplexes were added to HeLa cells.^[^ ^117^ ^]^ The uptake kinetics of lipoplexes followed an exponential increase, whereas those of low‐density lipoprotein presented linear uptake kinetics, indicating that the uptake of these nanoparticles was biphasic. An intracellular trafficking analysis of the lipoplexes provided a better understanding of their biphasic uptake. Following the downregulation of specific endocytic markers, such as clathrin heavy chain, C‐terminal‐binding protein‐1, Rac1, and Rabankyrin‐5 (a macropinocytosis regulator), the uptake of AF647‐siRNA carrying lipoplexes was reduced by ≈50–60%, indicating clathrin‐mediated endocytosis and macropinocytosis. Clathrin heavy chain silencing reduced the number of Rabankyrin‐5‐positive vesicles containing lipoplexes by approximately 70%, suggesting that clathrin‐mediated endocytosis stimulated macropinocytotic uptake of these nanocarriers. Taken together, the findings suggest that the first phase (≈1.5 h post‐exposure) of the uptake kinetics accounts for clathrin‐mediated internalization, while the second phase (≈2–6 h post‐exposure) is attributed to entry by macropinocytosis. Furthermore, 98% of the total uptake of liposomes was found to take place in the second phase, suggesting that macropinocytosis is a far more effective uptake mechanism than clathrin‐dependent endocytosis for the internalization of DLin‐MC3‐DMA:DSPC:cholesterol:DMG‐PEG lipoplexes.^[^ ^117^ ^]^ Nonetheless, it remains unclear how stimulation of macropinocytosis by clathrin could contribute to a therapeutic effect of DLin‐MC3‐DMA:DSPC:cholesterol:DMG‐PEG lipoplexes in vivo.

Another strategy to stimulate macropinocytotic uptake to improve transfection efficiency of nanocarriers is by functionalizing the particle membrane with stearylated‐arginine‐rich peptides, as suggested by Khalil and co‐workers.^[^ ^118^ ^]^ In this study, conjugation of EPC:cholesterol (7:3 molar ratio) and DOPE:CHEMS (9:2 molar ratio) lipoplexes with stearylated‐arginine‐rich peptides at high density was shown to increase macropinocytotic uptake and transfection efficiencies in NIH3T3 cells. Lipoplexes conjugated with octa arginine (R8) peptides at low density (0.86 mol%) were internalized predominantly via clathrin‐mediated endocytosis. Transfection with these lipoplexes resulted in low expression of the delivered luciferase gene, likely due to lysosomal entrapment and degradation. On the other hand, conjugation with high density (5.2 mol%) R8 peptide led to a three–fold increase in expression of luciferase after macropinocytotic uptake in NIH3T3 cells. As expected, the fusogenic lipid composition of DOPE:CHEMS lipoplexes exhibited a higher induction of gene expression than EPC:cholesterol lipoplexes. Overall, these data suggest that by changing the density of R8 peptides on the surface of lipoplexes, macropinocytotic uptake can be stimulated to increase therapeutic efficacy.^[^ ^118^ ^]^ This was also exemplified by a certain type of non‐viral delivery system, called multifunctional envelope‐type nanodevices (MEND). The device consists of a nucleic acid core wrapped in a lipid envelope that can be decorated with various functional moieties. Decoration of these devices with R8 peptides showed enhanced macropinocytotic uptake and lysosomal circumvention in cells.^[^ ^119^, ^120^, ^121^, ^122^ ^]^

Functionalization with R8 peptides was also deemed an effective approach in case of EV internalization through macropinocytosis. The cellular uptake and subsequent therapeutic efficiency of EVs isolated from HeLa cells improved significantly upon conjugation with stearylated R8 peptides in parental cells.^[^ ^123^ ^]^ Stearyl‐R8 peptide conjugation of EVs led to internalization through macropinocytosis and yielded higher uptake efficiency, compared to unmodified EVs. Subsequently, the effect of the ribosome‐inactivating protein saporin encapsulated into functionalized EVs was studied. Saporin acts as an anti‐cancer drug, showing a 40% increase in cytotoxicity in HeLa cells, compared to its incorporation into non‐functionalized EVs.^[^ ^123^ ^]^ These results were also confirmed in CHO‐K1 cells.^[^ ^124^ ^]^ By increasing the number of arginine residues in the peptide sequence, the cellular uptake efficiency was altered. R8‐conjugated EVs yielded the greatest level of internalization in CHO‐K1 cells, as compared to those conjugated with different length of peptide sequences. Although EVs decorated with hexadeca‐arginine (R16) peptides had a 40% lower cellular uptake than those decorated with R8, upon loading with saporin, R16 conjugation showed the highest decrease in cell viability (approximately 80%), compared to non‐decorated controls. Therefore, the therapeutic effectiveness of R16‐decorated EVs was higher than those decorated with peptide‐poor sequences potentially due to induced perturbation within the endosomal membrane, which subsequently promoted cytosolic release. Nonetheless, EIPA treatment inhibited uptake of R16‐functionalized EVs by 66%, compared to those conjugated with R8, whose internalization was completely abolished. This suggests that the enhanced therapeutic activity of R16‐decorated EVs is not acquired solely due to macropinocytotic uptake, but possibly due to the presence of other endocytosis mechanisms.^[^ ^124^ ^]^

Aside from R8 peptides, coiled‐coil forming peptides can also increase the cellular uptake of EVs through macropinocytosis. Stearyl (K_4_)‐functionalized EVs isolated from HeLa cells recognized E_3_ sequence‐modified EGF receptors expressed on the surface of MDA‐MB‐231 cells and consequently triggered endocytosis.^[^ ^125^ ^]^ Macropinocytotic uptake of engineered EVs was confirmed by EIPA treatment, which consequently reduced internalization by approximately 80%. This indicates that EV‐ and target cell‐functionalization with coiled‐coil peptides can induce the rearrangement of actin filaments in target cells, thereby potentiating uptake of natural drug delivery systems. Subsequently, saporin loading into the stearyl (K_4_)‐functionalized EVs enhanced the drug's bioactivity, compared to nonmodified EVs or MDA‐MB‐231 cells not expressing E_3_ sequence‐modified EGF receptors.^[^ ^125^ ^]^ Interestingly, conjugation of the same coiled‐coil peptide onto DOPC:DOPE:cholesterol liposomes enhanced uptake in HeLa cells through membrane fusion instead of macropinocytosis (discussed in Section 2.2.).^[^ ^126^ ^]^ In the case of liposomes, coiled‐coil forming peptides were conjugated to a cholesterol moiety via a polyethylene glycol (PEG) spacer, compared to the direct fusion of peptides to EVs, potentially causing differences in internalization mechanism. However, variations in lipid and protein composition should also be taken into consideration.^[^ ^126^ ^]^

Induction of macropinocytosis to enhance cellular uptake of drug delivery systems could be accomplished by external stimulation, without the need of functionalization. Activation of overexpressed epidermal growth factor receptors (EGFR) in cancer cells can be exploited to enhance cellular uptake of drug delivery systems through macropinocytosis. Specifically, interaction with the EGFR activates signal transduction through Rac, which in turn promotes the formation of lamellipodial extensions, needed for macropinocytosis.^[^ ^127^ ^]^ Exposure of A431 cells to HeLa cell‐derived EVs in the presence of EGF induced an increase in cellular uptake, with the overall effect depending on the presence of serum. In medium supplemented with serum, a 27‐fold increase in cellular uptake was reported, compared to a fivefold increase in the absence of serum.^[^ ^115^ ^]^ These data further highlight the effect of serum supplementation on cellular uptake. Interestingly, the encapsulation of EGF in EVs also exhibited an eightfold increase in internalization, compared to mock‐encapsulated EVs, suggesting that EV content is also able to enhance cellular internalization. Another factor capable of potentiating EV uptake is stromal cell‐derived factor‐1α. This cytokine binds to the CXCR4 chemokine receptor on the cells’ surface, triggering internalization of cargo through actin polymerization. Flow cytometry analysis showed that the addition of stromal cell‐derived factor‐1α to HeLa cells enhanced CD63‐GFP‐EV uptake more than twofold.^[^ ^115^ ^]^ These findings suggest that uptake of drug delivery systems through macropinocytosis represents an opportunity for therapeutic applications, as this mechanism is associated with high cellular uptake and vesicle leakiness during transport across the endo‐lysosomal pathway.

In summary, both liposomes and EVs are efficiently internalized and are able to deliver nucleic acids via macropinocytosis. Several approaches have been identified to enhance cellular uptake through macropinocytosis, such as engineering with R8 peptides or coiled‐coil peptides, EGF exposure or the presence of oncogenic K‐Ras mutation. However, coiled‐coil peptide conjugation only induces macropinocytosis in case of EVs, while the same conjugation of liposomes triggers uptake via membrane fusion. Nonetheless, the cause of these differences in uptake mechanisms remains unclear. Thereby, head‐to‐head comparisons are needed to determine which stimulation approach works best for natural and artificial drug delivery systems.

#### 2.1.5 Phagocytosis

Phagocytosis is the uptake mechanism responsible for the elimination of foreign objects within the body, depicted in **Figure** **5**. As drug delivery systems travel through the body, they can be recognized as foreign objects and tagged by biomolecules, termed opsonins.^[^ ^128^ ^]^ Upon interaction with phagocytic receptors, e.g., Fc‐, mannose‐, and scavenger receptors, a signaling cascade takes place, which is also involved in macropinocytosis. This cascade is responsible for actin re‐arrangement through the activity of the Ras superfamily of GTPases, including PI3K, Rac1, Cdc42, and Arf6 (see Figure 5‐1).^[^ ^24^ ^]^ Thereupon, the cell membrane extends around the particle (see Figure 5‐2), engulfing the cargo in a vesicle, of which scission is ensured by dynamin, thus securing proper formation of the vesicle (see Figure 5‐3).^[^ ^73^, ^88^, ^129^, ^130^ ^]^ Once the vesicle budded off from the plasma membrane, it is referred to as the phagosome and it is free to undergo further intracellular trafficking (see Figure 5‐4). Lysosomal membrane proteins and lytic enzymes accumulate into these phagosomes, generating so‐called phagolysosomes for degradation purposes (see Figure 5‐5).^[^ ^37^, ^131^, ^132^ ^]^ Proper maturation of phagosomes, among other processes, is dependent on Na^+^/H^+^ exchange, glycolysis, and calcium.^[^ ^132^, ^133^ ^]^ Importantly, phagocytosis is inherent to immune cells, such as macrophages, neutrophils, and monocytes to remove foreign objects, dead cells, and cell debris. Therefore, phagocytosis is an interesting strategy to gain access to immune cells or to target liver cells, since it is the final accumulation site of phagocytosed cargo.^[^ ^13^, ^134^ ^]^ In this section, we describe the approaches used to determine uptake via phagocytosis and discuss the studies that reported phagocytosis of liposomes and EVs.

Phagocytosis closely resembles macropinocytosis as an internalization mechanism and both mechanisms share constituents.^[^ ^101^ ^]^ Therefore, the pharmacological inhibitors used to identify these uptake mechanisms overlap. Inhibitors used to establish phagocytosis, as well as macropinocytosis, are based on interference with Na^+^/H^+^ exchanger, F‐actin polymerization, or PI3K activity, such as cytochalasins.^[^ ^28^ ^]^ For example, Cytochalasin B interferes with phagocytosis by disrupting actin polymerization through binding to the barbed end of actin filaments, therefore impeding internalization.^[^ ^135^ ^]^ Although phagocytosis is regulated by Na^+^/H^+^ exchange, the inhibitory effect of amilorides on this mechanism is inconsistent.^[^ ^28^, ^136^ ^]^ Other inhibitors, such as 2,4‐dinitrophenol and iodoacetate, interfere with glycolysis and oxidative phosphorylation, leading to uptake inhibition.^[^ ^137^, ^138^ ^]^ As phagocytosis is dependent on calcium signaling, chelating agents, e.g. EDTA, are sometimes used to identify phagocytosis.^[^ ^139^ ^]^ An alternative method to identify phagocytic uptake is co‐localization analysis of lipid nanoparticles with fluorescent latex beads, a model system for phagocytosis.^[^ ^140^, ^141^ ^]^ Altogether, these techniques allow us to determine the presence of phagocytosis in cells.

Typically, larger nanoparticles are taken up through phagocytosis, compared to smaller ones that would enter through clathrin‐ and caveolae‐mediated endocytosis. Nonetheless, Male et al. showed that human platelet cells internalized DSPC:cholesterol (2:1 molar ratio) liposomes of ≈74 nm diameter through phagocytosis.^[^ ^142^ ^]^ The authors incubated platelets with several phagocytosis inhibitors, such as EDTA, cytochalasin B, and 2,4‐dinitrophenol cosupplemented with iodoacetate, consequently reducing liposomal uptake by 48%, 46%, and 66%, respectively.^[^ ^142^ ^]^ Other examples of smaller lipid nanoparticles that are taken up via phagocytosis are K562 human erythroleukemia and MT4 T‐cell leukemia cell‐derived EVs of approximately 50–100 nm.^[^ ^143^ ^]^ These nanoparticles were efficiently internalized by phagocytic cells, such as RAW264.7‐, U937 monocyte‐derived‐, and J774A.1 macrophages, as compared to nonphagocytic cells, namely NIH3T3‐, Jurkat T‐, and 293T cells. In the non‐phagocytic cells, EVs remained attached to the outside of the plasma membrane. In phagocytic cells, EV internalization was mediated by PI3K activity, dynamin, and Tim‐4 receptors. As such, antibody blocking of Tim‐4 significantly inhibited cellular uptake of EVs, suggesting that these receptors are involved in the uptake of EVs via phagocytosis.^[^ ^143^ ^]^ Overall, these data suggest that liposomes and EVs can be taken up by immune cells via phagocytosis irrespective of their size.

EVs with different cellular origins, such as *P. gingivalis*, *Treponema denticola*, and *Tannerella forsythia*, were also reported to be cleared from systemic circulation by macrophages through phagocytosis.^[^ ^144^ ^]^ This indicates that gram‐negative bacteria‐derived EVs are highly prone to clearance by the immune system via phagocytosis. Although phagocytosis of EVs is desirable when targeting immune cells, EVs intended for therapeutic delivery in non‐immune cells are required to evade recognition by the immune system. To circumvent EV recognition, macrophages can be saturated through the preadministration of liposomes containing phosphatidylserine.^[^ ^145^, ^146^ ^]^ This was applied in vivo, where BALB/c mice were pre‐administered liposomes enriched in specific lipids, such as phosphatidylcholine or phosphatidylserine, prior to B16BL6‐derived EV exposure. Biodistribution analysis showed that EVs accumulated in different organs, which was quantified by measuring radioactivity of the (3‐^125^I‐iodobenzoyl)norbiotinamide label from the surface of EVs. Following treatment with phosphatidylserine‐rich liposomes, only 26% of EVs accumulated in the liver, as compared to the 40% EV accumulation after PBS and phosphatidylcholine‐rich liposome treatment. Additionally, the presence of EVs was approximately threefold higher in the blood, following treatment with phosphatidylserine‐rich liposomes, compared to the preadministration of PBS and phosphatidylcholine‐rich liposomes.^[^ ^145^ ^]^ These data suggest that macrophages are saturated by phosphatidylserine‐rich liposomes, allowing EVs to have a prolonged circulation time. Furthermore, clearance of EVs by immune cells will be a generally rare‐occurring event due to the presence of inherent CD47 within the surface‐protein composition of EVs, which acts as an invisibility cloak, thereby circumventing phagocytosis.^[^ ^116^, ^147^ ^]^

Overall, both liposomes and EVs can be internalized via phagocytosis by immune cells. However, studies on phagocytosis of liposomes are more limited and less recent. Furthermore, data indicate that the amount of phosphatidylserine in the lipid composition of drug delivery vesicles dictates their internalization via phagocytosis in immune cells. This mechanism can be exploited to actively target immune cells for immunotherapy, as well as saturation of the reticuloendothelial system to prolong circulation times of drug delivery systems.

#### 2.1.6 Membrane Components Aiding in the Endocytosis of Liposomes and EVs

##### Uptake Associated with Heparan Sulfate Proteoglycans (HSPGs)

Thus far, we discussed the classical endocytosis mechanisms of liposomes and EVs and the proteins responsible for internalization, which typically allows a distinction between uptake mechanisms. However, these entry routes do share certain factors that may cause multiple uptake mechanisms to occur simultaneously. These factors include, among others, surface receptors in the plasma membrane that are scattered across different types of endocytic pits, thereby enabling uptake through multiple mechanisms. One such class of surface receptors that aids in the endocytosis of nanocarriers is HSPGs. HSPGs are glycoproteins located on the outer surface of plasma membranes and serve as cellular surface receptors for various macromolecules, such as lipoproteins and growth factors.^[^ ^148^ ^]^ There are two types of HSPGs, the glycosylphosphatidylinositol‐anchored proteoglycans and syndecan transmembrane proteins. These serve as binding sites for growth factors, cytokines, and even viruses.^[^ ^149^, ^150^ ^]^ Both classes of HSPGs contain covalently bound heparan sulfate, a type of glycosaminoglycan that is exploited to actively target liposomes and EVs to cells expressing HSPGs.^[^ ^82^, ^151^, ^152^, ^153^ ^]^

To identify the involvement of HSPGs in the uptake of drug delivery systems, competition for these receptors can be assessed using highly anionic polysaccharides, e.g., exogenous heparin and fucoidan.^[^ ^154^ ^]^ Other methods include treatment with pharmacological inhibitors, such as sodium chlorate, which inhibit proteoglycan sulfation and suppress HSPG‐dependent uptake. Other inhibitors, e.g., heparinase, sequester surface HSPGs, thus hindering HSPG‐mediated endocytosis.^[^ ^82^, ^155^ ^]^ Additionally, knockdown or overexpression of HSPGs are different means to determine the involvement of HSPG in endocytosis. Below we will discuss the importance of HSPG‐mediated uptake for liposomes and EVs.

Over the years, an increasing number of studies have implicated HSPGs in the uptake of liposomes and lipoplexes, including their subsequent transfection efficiencies.^[^ ^150^, ^154^, ^155^, ^156^ ^]^ For example, Mounkes and co‐workers found that HSPG expression on the cellular membrane is necessary for the uptake of luciferase gene‐carrying DOTIM:cholesterol (1:16 molar ratio) lipoplexes.^[^ ^154^ ^]^ Genetically modified Raji cells expressing the HSPG syndecan‐1 were successfully transfected by these lipoplexes, compared to non‐modified recipient cells that lack this protein. Furthermore, the authors treated B16 cells with heparinase, which significantly inhibited luciferase protein expression after exposure to lipoplexes. This confirmed the importance of HSPG‐mediated internalization for gene delivery.^[^ ^154^ ^]^ To study the contribution of HSPGs in uptake, Korsholm and co‐workers used DDA cationic liposomes incubated with ovalbumin as a model antigen. Following heparin treatment, liposome uptake in murine bone marrow‐derived dendritic cells was reduced by almost an order of magnitude, as compared to untreated cells.^[^ ^157^ ^]^ Letoha et al. investigated which of the syndecan isoforms (1, 2, or 4) was the main contributor to the cellular uptake and therapeutic efficacy of commercially available DMRIE:cholesterol (1:1 molar ratio) liposomes.^[^ ^150^ ^]^ For this purpose, K562 cells, naturally lacking HSPGs, were transfected to express various syndecans and subsequently exposed to the liposomes. Flow cytometry data indicated that syndecan‐4 expressing cells had the highest cellular uptake, as shown by an approximately sevenfold increase in liposome internalization, compared to wild‐type cells. Furthermore, when liposomes were loaded with EGFP‐encoding plasmids, transfection efficacy improved in a similar manner. This indicates that syndecan‐4 is an interesting candidate to trial in nanomedicine applications. The authors also investigated the contribution of syndecans to other endocytosis mechanisms by assessing luciferase activity. After amiloride treatment, luciferase activity was reduced for all cells expressing syndecan‐1, −2, and −4. Potentially, this was due to interference with syndecan signaling and the subsequent actin rearrangement, leading to inhibition of liposome uptake via macropinocytosis.^[^ ^150^ ^]^ Despite the positive contribution of HSPGs in the internalization of liposomes in diverse cell lines, these proteins seem to negatively impact uptake of these nanocarriers in CHO‐K1 cells.^[^ ^156^ ^]^ Mutant CHO‐K1 cell lines, devoid of glycosaminoglycans and heparan sulfate polymerase activity, presented higher uptake with DOTAP and DOTAP:DOPE (1:1 molar ratio) lipoplexes than wild‐type cells. Additionally, luciferase expression was also increased following luciferase‐gene delivery by DOTAP and DOTAP:DOPE lipoplexes.^[^ ^156^ ^]^ The variations across the transfection abilities of lipoplexes in the presence of proteoglycans was possibly due to the differences in liposome composition, recipient cell type, and/or proteoglycan isoforms. More investigation is needed to determine exactly which factors are necessary for liposome uptake to predict successfully gene therapy efficiency.

The above examples highlight the involvement of HSPGs in the uptake of nonmodified liposomes. However, functionalized vesicles may also benefit from the presence of HSPGs to gain access to recipient cells. For example, high‐density R8‐conjugated lipoplexes were endocytosed by NIH3T3 cells, in a HSPG‐dependent manner. The R8 peptide‐conjugates were found to bind to surface HSPGs, which induced aggregation and phosphorylation of these proteins. As a result, the liposomes were internalized through macropinocytosis (see also section 2.1.4 for more information on macropinocytosis).^[^ ^118^ ^]^ Another functionalization moiety, namely the RRRRRRGGRRRRG cell‐penetrating peptide, enhanced cellular internalization of DOPE:cholesterol:DMPG (9:2:2 molar ratio) lipoplexes in murine melanoma B16F10 cells.^[^ ^158^ ^]^ Compared to nonfunctionalized lipoplexes, surface conjugation with this cell‐penetrating peptide enhanced cellular uptake in a time‐dependent manner. Uptake of the lipoplexes was drastically impaired by heparin and amiloride treatment, inhibiting internalization by approximately 95% and 80%, respectively. Together, these results suggest that cell‐penetrating peptide‐conjugated lipoplexes are internalized in a HSPG‐dependent manner via macropinocytosis. Analysis of their transfection efficacy revealed that the luciferase activity of luciferase‐expressing B16F10 cells was knocked down 70% after incubation with cell‐penetrating peptide‐conjugated lipoplexes containing luciferase‐targeted siRNA. Hence, HSPG‐mediated macropinocytotic uptake leads to successful gene delivery in B16F10 cells.^[^ ^158^ ^]^ Similar knockdown efficiency (≈72%) was achieved in EGFP stably transfected MDA‐MB‐231 cells by DOPG:DOPE (40:60 molar ratio) lipoplexes after macropinocytotic uptake in a HSPG‐dependent manner.^[^ ^159^ ^]^ Here, despite the anionic surface charge of lipoplexes, HSPG‐mediated internalization was achieved through loading of nanocarriers with high Ca^2+^ content (lipid:Ca^2+^:siRNA = 1.3:2.5:1 molar ratio). In comparison, their Ca^2+^‐poor counterparts (lipid:Ca^2+^:siRNA = 1.3:0.3:1 molar ratio) exhibited internalization solely through macropinocytosis and showed almost twofold lower cellular uptake than those rich in Ca^2+^ content.^[^ ^159^ ^]^

Targeting HSPG‐mediated entry through surface functionalization can aid in enhancing liposome uptake and consequently improve the cytotoxic effect of cancer treatments. For example, SPC:cholesterol:D,L α‐tocopherol (1:0.2:0.001 molar ratio) liposomes functionalized with protein transduction domains, such as Antennapedia and HIV transactivator of transcription (TAT), elicited a 15 to 25‐fold higher uptake in B16F1 cells through HSPG‐mediated endocytosis than their unmodified counterparts. Moreover, the cytotoxic effect of these drug‐loaded liposomes was reduced in the presence of heparin, evidencing the relevance of HSPG‐mediated uptake for drug delivery.^[^ ^151^ ^]^ In addition, Kuo and co‐workers showed enhanced cellular internalization of liposomal doxorubicin via HSPGs present on A549 cells by inserting glycosaminoglycan‐binding peptides into the membrane of DSPE‐PEG_2000_ (1:1.1 molar ratio) liposomes.^[^ ^160^ ^]^ In contrast, cellular uptake efficiency of these liposomes in the presence of heparan sulfate was diminished. However, uptake was unaffected by the presence of other glycosaminoglycans, namely chondroitin sulfate type B and hyaluronic acid. These data indicate that glycosaminoglycan‐binding peptides are specific for heparan sulfates. In addition, the authors used spheroid co‐cultures to test the penetration and therapeutic efficacy of glycosaminoglycan‐binding peptide‐functionalized HSPC:cholesterol:DSPE‐mPEG_2000_ (3:2:0.045 molar ratio) liposomes. In heterospheroids composed of A549 and NIH3T3 cells, the glycosaminoglycan‐binding peptides aided in a deeper penetration and increased therapeutic efficacy of liposomes, compared to non‐conjugated or PEGylated controls. Furthermore, the density of glycosaminoglycan‐binding peptides dictated the penetration depth and cellular uptake efficiency in heterospheroids.^[^ ^160^ ^]^ Another example of exploiting HSPG‐mediated uptake was inspired by the invasion abilities of *Plasmodium berghei*. Robertson and co‐workers reported that intravenous injection of liposomes, containing the *Plasmodium*‐derived amino acid sequence acetyl‐CKNEKKNKIERNNKLKQPP‐amide, actively targeted the liver through interaction with HSPGs.^[^ ^161^, ^162^ ^]^ As such, this amino acid sequence was shown to have an affinity for HSPGs found on liver.^[^ ^161^ ^]^

HSPGs also prove relevant in EV internalization.^[^ ^163^, ^164^, ^165^ ^]^ Mutant CHO‐K1 cell lines, lacking glycosaminoglycans and heparan sulfate, showed a reduced uptake of *T. vaginalis*‐derived EVs by 70% and 45%, respectively, compared to the wild‐type cells.^[^ ^82^ ^]^ Similarly, the presence of exogenous heparan sulfate impaired the internalization of EVs in BPH‐1 cells, due to competition for the cells’ HSPG surface receptors. Further investigations revealed that TVAG_157 940, the most abundant protein of the three glucanotransferases present in the EV proteome, was the ligand responsible for HSPG binding.^[^ ^82^ ^]^ Atai and co‐workers investigated the role of HPSGs in the uptake of tumor‐ and non‐tumor‐derived EVs using heparin.^[^ ^166^ ^]^ Even the lowest concentration of heparin used (0.1 µg mL^−1^), negatively affected the internalization of EVs. At a tenfold higher heparin concentration, the uptake was reduced by 90% and 95% in glioblastoma (GBM) U87‐MG and GBM11/5 cells, respectively. The authors attributed this reduction to two mechanisms. Firstly, heparin binds to HSPGs present on the surface of EVs causing them to aggregate, thereby reducing uptake. Formation of these aggregates was confirmed by their clustering shown by TEM imaging and differences in zeta potential, ranging between −39.1 and −32.6 mV, in the absence or presence of heparin, respectively. Secondly, heparin was used as a competing molecule for the HSPG cell receptors and therefore lowered the receptors’ availability for EVs to interact with the cell plasma membrane. This HSPG‐associated uptake was responsible for EV‐mediated transfer of genetic material. After exposure to EGFRvIII mRNA‐containing EVs, isolated from Gli36 cells, the expression of EGFRvIII in U87‐MG cells in the presence of exogenous heparin was half of that in the absence of heparin.^[^ ^166^ ^]^ In a study conducted by Christianson et al., co‐localization measurements suggested that two major types of cell surface HSPGs, syndecans and glycosylphosphatidylinositol‐anchored membrane proteins, mediated the cellular internalization of cancer‐derived EVs in U87‐MG cells.^[^ ^153^ ^]^ These results were confirmed by depletion of HSPG, through heparinase treatment of GBM cells, leading to an approximately 50% reduction in EV uptake, compared to untreated controls. The internalization route proved to be dependent on HSPGs of recipient cells, given that mutant CHO‐K1 cells devoid of heparan sulfate 2‐O‐sulfation and N‐sulfation exhibited significantly lower EV uptake. In contrast to the cellular HSPGs being relevant for EV uptake, EV‐associated HSPGs were shown to have no relevance for uptake by acceptor cells, as enzymatic depletion of HSPG did not alter their uptake.^[^ ^153^ ^]^ Although HSPGs from the surface of EVs did not seem to affect cellular internalization, recent findings suggest the opposite. Williams and co‐workers treated murine hepatic cell‐derived EVs with glycosidases, namely PNGase f and neuraminidase, to cleave n‐glycans and terminal sialic acids from the surface of EVs, respectively.^[^ ^167^ ^]^ Upon cleavage, the uptake of EVs in various cell lines (Huh7‐, Sk Hep‐1 hepatic cells, U2‐OS osteoblast‐like cells, and M1 fibroblastoid cells) increased in most cases. This suggests that the presence of HSPGs on the surface of EVs impedes HSPG‐mediated uptake of these nanocarriers. The same study also showed that this treatment could lead to a preference of EVs being taken up by specific cell lines in vitro. However, it remains unclear whether this observed effect is due to alteration of glycan‐receptor interactions or surface charge modifications.^[^ ^167^ ^]^

To fully elucidate HSPG‐mediated internalization, there is a growing interest in finding specific ligands that are involved in EV‐cell interaction and in their subsequent internalization mechanism. Fibronectin is one of such ligand identified to mediate EV‐cell interaction by bridging the interaction between HSPGs located on EVs and cellular plasma membranes.^[^ ^168^, ^169^ ^]^ Recently, the DAL‐¼.1B skeletal protein was recognized to control HSPG expression, which in turn mediated the cellular internalization of EVs from different cellular origins.^[^ ^170^ ^]^ DAL‐¼.1B is located underneath the membrane, connecting transmembrane proteins to the cytoskeleton, thereby regulating their function. Upon overexpression of DAL‐¼.1B in human lung cancer H292 cells, HSPG expression increased more than twofold, which in turn more than doubled the uptake of EVs derived from A549, MDA‐MB‐231, MCF‐7, H1299, and H292 cells. These results were further supported by the decrease in EV uptake after siRNA‐mediated knockdown of DAL‐¼.1B.^[^ ^170^ ^]^ Another factor identified to mediate the cellular internalization of EVs in a HSPG‐mediated manner is syntenin. Within cells, this scaffold protein binds to clustered syndecans through which the uptake and recycling of syndecans and syndecan cargo, such as EVs, are regulated. Upon siRNA‐mediated knockdown of syntenin in mouse embryonic fibroblasts, uptake of EVs derived from MCF‐7 breast cancer cells decreased by 64%, confirming the importance of this protein in HSPG‐mediated internalization. As such, the presence of syntenin in cells may be exploited to enhance the cellular internalization of EVs for drug delivery purposes.^[^ ^171^ ^]^ Overall, uptake mechanisms mediated by plasma membrane‐resident HSPGs play an important role in the uptake of EVs. Furthermore, the naturally occurring ligands within EVs could be exploited to mediate their uptake, which is an important advantage, compared to liposomes that would require functionalization to achieve such purposes.

In EVs derived from tumor cells, the intrinsic membrane proteins present, including HSPGs, potentially endow the vesicles with cellular selectivity. Gan et al. developed a hybrid lipid‐EV formulation by fusing the membrane of human hepatocellular carcinoma (HCC) Sk‐hep1 cell‐derived EVs with DPPC phospholipid (1:1 w/w).^[^ ^172^ ^]^ The resulting hybrid DPPC:EVs were compared to DPPC liposomes for cellular uptake, amongst others. The physicochemical characteristics of the two vesicle types were comparable, with a size of 117 nm and zeta potential of −25.9 ± 0.6 mV for DPPC:EVs and −14.9 ± 0.9 mV DPPC liposomes. Uptake of DPPC:EVs in Sk‐hep1 cells was twofold higher than DiI‐labeled DPPC liposomes. Furthermore, the cellular internalization mechanism of DPPC:EVs and DPPC liposomes in parental cells was shown to be mediated by HSPGs, at least to some degree. Flow cytometry analysis revealed that heparin treatment to block HSPGs in Sk‐hep1 cells, reduced uptake of DiI‐labeled DPPC:EVs approximately threefold, compared to untreated cells. In contrast, though heparin treatment resulted in a significant reduction in uptake of DiI‐labeled DPPC liposomes, this was to a far lesser extent than for DPPC:EVs. These results indicate that the inherent HSPGs in the DPPC:EV membrane increase cellular uptake. Interestingly, Sk‐hep1 cells exhibited a 4.3‐fold higher HSPG expression compared to LO2 human fetal hepatocytes.^[^ ^172^ ^]^ The authors showed that DPPC:EVs resemble the parental cell line, potentially resulting in enhanced and specific uptake in parental cells. Indeed, uptake of DPPC:EVs in Sk‐hep1 cells demonstrated a 3.7‐fold increase compared to LO2 cells. This preference of DPPC:EVs for uptake in cells overexpressing HSPGs was further substantiated by a 3.2‐fold higher uptake of DPPC:EVs in HepG2 HCC cells, which resemble Sk‐hep1 in HSPGs expression levels.^[^ ^172^ ^]^ Overall, these results indicate at least a certain level of cellular selectivity in the uptake of DPPC:EV hybrid vesicles, though the full impact warrants further investigation.

In summary, different moieties can be considered for liposomal functionalization to actively target HSPG‐mediated endocytosis and enhance cellular uptake, such as protein transduction domains and glycosaminoglycan‐binding peptides. In addition to membrane functionalization, loading of *Plasmodium*‐derived amino acid sequences into liposomes, also deemed effective to actively target surface HSPGs. However, whether these functionalizing entities are cell type‐dependent or not, remains to be elucidated. In comparison, native surface proteins found in EVs can facilitate HSPG‐mediated uptake, which offers EVs an advantage compared to liposomes. Nonetheless, the HSPGs present in EV membranes seem to pose a controversy, regarding the stimulation or hindrance of their uptake via HSPG‐mediated internalization. To the best of our knowledge, there are no studies on the functionalization of EVs to actively target HSPG receptors. Thus, more research is needed to determine whether the above‐mentioned functionalizing techniques have the same effect in EV uptake and to be able to compare liposomes to EVs as drug delivery rivalries.

##### Uptake Associated with Lipid Raft‐Mediated Internalization

Aside from HSPG receptors, lipid raft domains are another plasma membrane constituent associated with classical endocytosis mechanisms. Similarly to HSPGs, these domains are spread across the plasma membrane and contribute to other uptake mechanisms, e.g., caveolae‐dependent endocytosis, as lipid rafts are particularly enriched in caveolae.^[^ ^173^ ^]^ This led to the so‐called lipid raft‐dependent endocytosis. Lipid rafts contain clusters of lipids enriched in sphingolipids with long unsaturated hydrocarbon chains, which associate with cholesterol and proteins spread across the site of the membrane.^[^ ^173^, ^174^ ^]^ The asymmetric distribution of sphingolipids and cholesterol across the two lipid layers of the membrane allows the internalization of molecules through the dynamic clustering of these factors, leading to the formation of lipid rafts that move within the fluidic bilayer.^[^ ^175^ ^]^ In addition to these lipids, lipid rafts are also enriched in glycosylphosphatidylinositol‐anchored proteins that are located in the outer leaflet of the membrane.^[^ ^173^ ^]^ These microdomains serve as a platform for the assembly of various signaling molecules, which in turn mediate signal transduction and membrane trafficking.^[^ ^176^ ^]^ Lipid raft domains are used by both viruses and nanocarriers to gain access to cells. This type of internalization is mostly associated with caveolae‐dependent internalization. However, it can be involved in other uptake mechanisms, like clathrin‐ and caveolae‐independent endocytosis, macropinocytosis, and phagocytosis.^[^ ^173^, ^175^, ^176^, ^177^, ^178^, ^179^, ^180^ ^]^ Below, we address lipid raft‐mediated uptake of liposomes and EVs, as well as the impact of the entry pathway for transfection. Furthermore, methods and strategies are described to enhance delivery by exploiting this pathway.

To identify lipid raft‐dependent uptake, pharmacological inhibitors that sequestrate cholesterol from the plasma membrane, such as methyl‐β‐cyclodextrin, are employed to inhibit internalization.^[^ ^176^ ^]^ Another way to recognize lipid raft‐mediated uptake is by establishing co‐localization with cholera toxin subunit B, which is a marker for lipid raft‐mediated endocytosis.^[^ ^181^ ^]^ One should keep in mind that cholesterol depletion and cholera toxin subunit B co‐localization are also used to establish caveolae‐mediated internalization, thus it may be challenging to fully differentiate caveolae‐dependent from caveolae‐independent lipid raft‐mediated endocytosis.

Lipid raft‐mediated endocytosis of liposomes was found to occur in various cell lines. For example, Li et al. demonstrated that lipid raft‐mediated endocytosis was the prevailing mechanism for the internalization of Tween80:DOTAP:DPPC:cholesterol (3:40:20:37 mol%) and DOTAP:DOPE:cholesterol:DSPE‐PEG (40:40:19:1 mol%) liposomes in HepG2 and A375 cells. Incubation with methyl‐β‐cyclodextrin, reduced liposome uptake by 50–70%, while genistein had no significant effect, suggesting lipid raft‐associated entry in a caveolae‐independent manner.^[^ ^182^ ^]^ Lipid raft‐dependent endocytosis of liposomes also plays a role in the internalization of functionalized delivery systems. In a study conducted by Qhattal and co‐workers, PC:PE:cholesterol (65:5:30 molar ratio) liposomes were functionalized with different densities of hyaluronan (0.2 and 1.5 mg) and subsequently cellular internalization was determined in A549 cells.^[^ ^183^ ^]^ Of all the endocytosis inhibitors used, only methyl‐β‐cyclodextrin significantly reduced the percentage of internalized liposomes (≈40–75% inhibition) of hyaluronan conjugated nanocarriers, indicating cholesterol dependency. None of the other inhibitors affect caveolae‐mediated endocytosis or macropinocytosis, which are also cholesterol‐sensitive mechanisms, hindered liposomes uptake. Together, this suggests that the internalization of hyaluronan‐decorated liposomes was mediated by lipid raft domains.^[^ ^183^ ^]^ In another study, Gunawan et al. showed that active targeting of lipid raft‐mediated endocytosis may improve cellular uptake efficiency of liposomes in endothelial cells in inflammatory conditions.^[^ ^184^ ^]^ The researchers studied the uptake mechanism of DOPC or DPPC liposomes decorated with antibodies against cell adhesion molecule‐1 (CD54) and endothelial leukocyte adhesion molecule‐1 (CD64E), which are located within lipid rafts.^[^ ^185^ ^]^ As such, the presence of these receptors in lipid rafts of injured or inflamed endothelial cells successfully enhanced cellular internalization. After methyl‐β‐cyclodextrin treatment, the uptake of the liposomes was reduced, which may imply lipid raft‐mediated endocytosis as the predominant uptake mechanism.^[^ ^184^ ^]^

Similar to liposomes, uptake of EVs may be dictated by lipid rafts. As such, EVs derived from various pathogens use specific factors for internalization.^[^ ^186^ ^]^ For example, EVs derived from *P. gingivalis* contain fimbriae on their surface.^[^ ^187^, ^188^ ^]^ These structures are deemed essential for the internalization of *P. gingivalis*‐derived EVs by HeLa and immortalized human gingival epithelial (IHGE) cells, as those isolated from fimbriae‐null strains were reported to have negligible interactions, though data supporting this were not shown.^[^ ^95^ ^]^ Furthermore, the uptake was likely to be mediated by lipid rafts, given that cholesterol sequestration by methyl‐β‐cyclodextrin treatment almost completely abolished EV entry. Similarly, cytochalasin D and wortmannin incubation also halted EV uptake. This indicates that *P. gingivalis*‐derived EVs were internalized via macropinocytosis through lipid raft domains in HeLa and IHGE cells. Lipid raft‐mediated endocytosis was also confirmed through the co‐localization of EVs, stained with antibodies against native fimbria, with GFP‐labeled glycosylphosphatidylinositol anchored proteins, located in lipid raft domains.^[^ ^95^ ^]^ Thus protein composition of EVs can mediate uptake through lipid raft‐dependent endocytosis.

Cancer cell‐derived EVs also require lipid raft‐associated pathways for their internalization. For instance, approximately 20% of GBM‐derived EVs, taken up by HUVEC and U87 MG cells, co‐localized with cholera toxin subunit B.^[^ ^85^ ^]^ The involvement of lipid raft domains in EV internalization was further supported by methyl‐β‐cyclodextrin treatment, reducing EV uptake by ≈40–60% in HUVECs and U87‐MG cells. The same study showed that EVs may have triggered lipid raft‐mediated uptake through signaling activation of extracellular signal‐regulated kinase‐(ERK)1/2‐dependent pathways. Generally, activation of these pathways is followed by the activation of heat shock protein 27, which plays a role in cytoskeleton rearrangement needed for endocytosis. Furthermore, caveolin‐1 co‐localized with ERK‐1/2 near the plasma membrane and suppressed its activation, thereby partly inhibiting uptake of EVs.^[^ ^85^ ^]^ Plebanek and co‐workers determined that the scavenger receptor type B‐1, residing in cholesterol‐rich membrane microdomains, is involved in lipid raft‐mediated uptake of A375 melanoma cell‐derived EVs in human dermal microvascular endothelial‐, A375 cells, and RAW264.7 macrophages.^[^ ^189^ ^]^ This scavenger receptor has an affinity for high‐density lipoproteins. In order to establish its role in cellular internalization, biomimetic high‐density lipoprotein‐like nanoparticles were synthesized. Coincubation of lipoprotein‐like nanoparticles with EVs, led to the cellular association of lipoprotein‐like nanoparticles and to the subsequent clustering of the scavenger receptor type B‐1, while inhibiting EV uptake in cells.^[^ ^189^ ^]^

In summary, both natural and synthetic drug delivery systems are, at least to some extent, internalized through lipid raft‐mediated endocytosis. Although surface functionalization to target lipid raft‐dependent uptake seems to enhance cellular uptake of liposomes in cells, the means to enhance EV uptake remain to be elucidated. Similarly, more research is needed to explore this mechanism for biomedical applications, such as nucleic acid delivery, and to establish specific approaches to distinguish lipid raft‐mediated internalization from other mechanisms. Nonetheless, targeting lipid raft‐mediated endocytosis through decorating moieties showed promising results in enhancing cellular uptake of lipid‐based carriers, which could be exploited for treating cells under inflammatory conditions.

### 2.2 Membrane Fusion

Although endocytosis prevails as the primary uptake mechanism of different drug delivery systems, in some cases direct membrane fusion may take place. Upon close proximity, liposomes and EVs can fuse with the plasma membrane, releasing their cargo directly into the cytosol and circumventing the endo‐lysosomal pathway. As such, membrane fusion would be especially useful for delivering therapeutic molecules directly into the cells’ cytoplasm, as lysosomal degradation is evaded.^[^ ^190^, ^191^ ^]^ To detect membrane fusion of lipid nanoparticles, lipid mixing assays using self‐quenching fluorophores are used.^[^ ^192^ ^]^ As the drug delivery system interacts with the plasma membrane, the fluorophores embedded in the membrane of drug delivery systems are being diluted, thereby reducing self‐quenching and enabling a detectable fluorescent signal.^[^ ^192^ ^]^ Alternatively, the pharmacological inhibitor botulinum toxin serotype A can be used for establishing membrane fusion, as treatment hinders membrane fusion activities by blocking the activity of SNARE proteins, located on the surface of membranes.^[^ ^193^ ^]^ Below, we review the uptake of natural and artificial lipid‐based nanoparticles through membrane fusion, highlighting the cargo delivery to recipient cells.

The cellular internalization of liposomes generally takes place via endocytosis and there are only a handful of reported cases in which uptake occurred via membrane fusion, indicating that this mechanism is a rare event. For instance, Lu et al. showed that R18‐labeled EV‐mimicking lipoplexes, composed of DOPC:SM:cholesterol:DOPS:DOPE (21:17.5:30:14:17.5 mol:mol), exhibited some fusion activity in A549 cells, established through a membrane fusion assay.^[^ ^86^ ^]^ Still, a significant inhibition of uptake (≈30–50%) was found after indomethacin and amiloride treatment, indicating that caveolae‐mediated endocytosis and macropinocytosis play a more important role in the internalization of these lipoplexes.^[^ ^86^ ^]^ On the other hand, R18‐labeled Lipofectamine 2000, DOTAP:DOPC:cholesterol (40:40:20 molar ratio), and DOPC:cholesterol (70:30 molar ratio) lipoplexes generated a negligible difference in fluorescence following incubation with A549 cells, suggesting a lack of fusion activity. Similarly, there was no significant increase in the fluorescence of R18‐labeled EV‐mimicking lipoplexes with HUVECs.^[^ ^86^ ^]^ Overall, these data indicate that membrane fusion does not occur frequently and for liposomes is cell type‐dependent. Since membrane fusion is an energy‐independent mechanism unlike endocytosis, Huth and co‐workers assessed fusion activity of DOPE:CHEMS (3:2 molar ratio) liposomes in COS‐7 and HUVECs during incubation at 4 °C.^[^ ^74^ ^]^ Uptake of these nanocarriers was partially inhibited, suggesting that membrane fusion may have been partly involved in the internalization of liposomes, but endocytosis still remains the predominant mechanism.^[^ ^74^ ^]^ Changing lipid composition was shown to change the internalization mechanisms of liposomes in CHO‐K1 cells from endocytosis to membrane fusion.^[^ ^194^ ^]^ Confocal images showed that DOPC:DOTAP:TFPE (1:1:0.1 molar ratio) liposomes were located inside the cytoplasm, seen as punctae, whereas those composed of DOPE:DOTAP:TFPE (1:1:0.1 molar ratio) fused with cells, recognized by staining of the plasma membrane. Based on the fluorescence intensity values of liposomes, it was established that by exchanging DOPC with DOPE in the lipid composition of liposome fusion activity was increased from 10% to 90%.^[^ ^194^ ^]^ Therefore, uptake of liposomes through membrane fusion can be achieved by offering a suitable lipid composition.

As membrane fusion leads to direct cytosolic delivery of encapsulated cargo, research has focused on inducing this mechanism by engineering drug delivery systems. For example, liposome conjugation with lectins and PEGylation of cell membranes can enhance fusion activity, which can be exploited to improve cargo delivery to the cytoplasm.^[^ ^195^ ^]^ As such, Szoka et al. used LC:PG:PC:cholesterol (1:0.5:8.5:8 molar ratio) unilamellar liposomes with two lectins, either Ricinus communis agglutinin I or concanavalin A, conjugated to their surface.^[^ ^195^ ^]^ These lectins were shown to orchestrate cellular binding to glycoproteins and glycolipids in BG‐9 human fibroblasts, NIL‐8M2 hamsters, and L‐929 mouse cells. Subsequently, treatment of these cells with PEG was shown to enhance the fusion activity of liposomes.^[^ ^195^ ^]^ PEG was suggested to expose the lipids within the plasma membrane by segregating surface proteins, allowing membrane fusion to take place. This activity was established following transfer of fluorescence from DiI‐C16[3]‐labeled liposomes to the plasma membrane or by detecting the presence of fluorescently labeled albumin after liposomal delivery. Surface functionalization of liposomes triggered membrane fusion and subsequent transfer of membrane constituents and vesicle cargo, while the absence of the conjugated moieties halted fusion activity.^[^ ^195^ ^]^ Another study focused on liposomes engineered with either cleaved or uncleaved hemagglutinin of influenza virus strains and tested their fusion activity in chicken embryonic fibroblasts.^[^ ^196^ ^]^ The engineered liposomes containing cleaved hemagglutinin presented fusion activity within the first 15 min of exposure at room temperature. When the temperature was raised to 37°C, fusion events were no longer detectable, which was attributed to their rapid occurrence, hence the inability to record such fast events. In contrast, liposomes containing uncleaved hemagglutinin strongly adhered to the cell surface, but did not display any fusion activity. As such, it was suggested that the hydrophobic segment of hemagglutinin was exposed to proteolytic cleavage, which in turn triggered fusion.^[^ ^196^ ^]^

A different approach to induce uptake via membrane fusion, similar to macropinocytosis, is by taking advantage of coiled‐coil lipopeptides. For example, Yang and co‐workers assessed cytosolic delivery of (EIAALEK)_3_‐conjugated DOPC:DOPE:cholesterol (50:25:25 mol%) liposomes in vivo and in vitro.^[^ ^126^ ^]^ Fusion of these liposomes with the cell membrane was facilitated by complementary coiled‐coil lipopeptides, embedded in both the liposomal formulations, as well as the cell membrane, thus enabling targeted fusion. A variety of cell lines such as HeLa, CHO‐K1, and NIH3T3 cells were decorated with (KIAALKE)_3_ peptides, which fused with liposomes functionalized with (EIAALEK)_3_ complementary sequences. Confocal images revealed that 1 mol% nitrobenzoxadiazole (NBD)‐labeled DOPE liposomes successfully fused with the plasma membrane, as fluorescence transfer of NBD into the cell was confirmed. Furthermore, the aqueous content of either the nucleic acid stain propidium iodide or TOPRO3 was present in the cytosol, confirming the membrane fusion of liposomes with cells. Similar results were observed in zebrafish embryos by NBD fluorescence transfer and doxorubicin labeling encapsulated in liposomes.^[^ ^126^ ^]^

Membrane fusion has also been reported for EV internalization. For example, metastatic melanoma cell‐derived EVs labeled with the self‐quenching probe R18 exhibited membrane fusion in parental cells.^[^ ^197^ ^]^ Furthermore, EVs isolated from metastatic melanoma cells cultured under acidic conditions were taken up through membrane fusion more readily in recipient cells than those isolated from cells cultured under buffered conditions at neutral pH. This was supported by the 1.5‐fold increase in fluorescence after exposure to EVs cultured in acidic conditions, compared to those of buffered conditions, suggesting an increase in membrane fusion. The enhanced fusion activity was suggested to be due to the increase in membrane rigidity of EVs isolated from low pH conditions, as a consequence of enhanced sphingomyelin/ganglioside GM3 content in EV membrane composition.^[^ ^197^ ^]^ Along the same line, phosphatidylserine and anionic phospholipids of biological membranes were also found to aid in membrane fusion and may be one of the key mediators of this uptake mechanism.^[^ ^198^, ^199^ ^]^ Therefore, EVs containing large amounts of phosphatidylserine, may represent an ideal candidate for direct cytosolic delivery.^[^ ^200^ ^]^ Furthermore, filipin treatment reduced the membrane fusion activity of EVs isolated under buffered conditions by 50%, compared to untreated cells, suggesting that cholesterol is important for internalization. Subsequent analysis revealed that, due to enhanced membrane fusion of EVs isolated from cells grown under acidic conditions, these vesicles were able to transfer more protein content, such as caveolin‐1 or lysosomal‐associated membrane protein‐2, than those isolated under buffered conditions.^[^ ^197^ ^]^ Another example of EV uptake via membrane fusion was provided by Obregon and co‐workers. The authors showed that EVs isolated from human‐activated dendritic cells fused with resting dendritic cells as an alloantigen transferring mechanism. To confirm the membrane fusion activity of EVs, dendritic cells were stimulated with DiI‐labeled lipopolysaccharide which continued to be present in the isolated EVs. After exposing DiI‐labeled EVs to DiO‐labeled dendritic cells, co‐localization analysis presented overlap between the fluorescent signal of EVs and the cell membrane.^[^ ^201^ ^]^ Additionally, Montecalvo and co‐workers looked into the communication between bone marrow dendritic cells via miRNA delivery through EVs. Using a de‐quenching assay, the authors showed fusion activity of bone marrow dendritic cell‐derived EVs with recipient dendritic cells. Further analyses showed that pretreatment of cells with filipin decreased the signal substantially, indicating cholesterol dependency of membrane fusion. After membrane fusion, EVs were able to effectively transfer miR‐451 and miR148a directly into the cytosol of other recipient dendritic cells. These dendritic cells naturally express very low, if any, of these miRNAs. A miRNA reporter assay confirmed that the transfer resulted in repressed target mRNA, underlining the potential of membrane fusion in gene therapy.^[^ ^202^ ^]^ Hence, membrane fusion activity is of great importance for direct cytosolic delivery of EV content in recipient cell lines. Additionally, one of the factors that seem to influence this uptake mechanism is lipid composition, which can be used to benefit therapeutic delivery.

Alternatively to lipid composition, EV membrane components can also mediate membrane fusion activities between EVs and cells.^[^ ^203^ ^]^ For instance, membranes of EVs isolated from proinflammatory RAW264.7 macrophages are enriched in C─C chemokine receptor type 2. Meanwhile, levels of its ligand are elevated in tumor tissues, compared to those of healthy liver and ovary.^[^ ^204^ ^]^ Receptor–ligand interaction was found to mediate selective uptake of EVs in tumor cells through membrane fusion. After doxorubicin loading into EVs, the vesicles were treated with botulinum toxin serotype A to establish fusion activity, upon which only 25% of cells presented doxorubicin‐positive signals, compared to untreated controls, confirming membrane fusion activity. Furthermore, uptake enabled direct cytosolic release of doxorubicin and accumulation in nuclei, which in turn exerted robust antitumor efficacy and improved survival rate in tumor‐bearing mice. In comparison, a commercial liposomal doxorubicin formulation had lower cellular accumulation, decreased survival rate and higher multidrug resistance incidences than EVs.^[^ ^204^ ^]^ Another EV membrane constituent identified to regulate membrane fusion activity is P‐selectin glycoprotein ligand‐1.^[^ ^205^ ^]^ In activated platelets, antibody treatment against this ligand lowered fusion activity by approximately twofold of THP‐1 monocyte‐derived EVs, compared to untreated controls. This indicates that THP‐1 monocyte‐derived EVs require the presence of P‐selectin glycoprotein ligand‐1 to fuse with activated platelets. Following membrane fusion, the recipient cells acquired EV content, such as tissue factors along with acquiring P‐selectin glycoprotein ligand‐1 itself on their membrane. The same study identified another factor involved in the membrane fusion of these EVs, that of phosphatidylserine, established through the blockage of its receptor with Annexin V. These findings confirm the role of P‐selectin glycoprotein ligand‐1 and phosphatidylserine in the uptake of THP‐1‐derived EVs by activated platelets.^[^ ^205^ ^]^ Therefore, it is pivotal to identify the factors that are able to mediate the membrane fusion activities of EVs, as these tools can be exploited to induce direct cytosolic delivery of cargo.

Based on current findings, membrane fusion appears to occur to a lesser extent than endocytosis for the uptake of liposomes and EVs. Still, membrane fusion should be considered for cargo transfer, especially for those whose therapeutic action resides in the cytosol. Membrane fusion also holds great promise to bypass lysosomal accumulation, therefore circumventing cargo degradation. However, drug delivery systems do require functionalization to improve membrane fusion efficiency instead of being overruled by endocytosis. Nonetheless, more work needs to be done to establish the efficiency of membrane fusion for drug delivery applications.

## 3 Intracellular Fate of Drug Delivery Systems

The “magic bullet” concept describes the ideal scenario in which drugs can be delivered at the site of interest without causing harm to healthy cells or tissues. Following internalization of drug delivery systems by recipient cells, efficacy is achieved once the therapeutic cargo reaches the desired target organelle, such as mitochondria, endoplasmic reticulum or, in numerous cases, the nucleus.^[^ ^206^ ^]^ Before reaching an intracellular target, the plasma membrane is one of the barriers that need to be overcome. In the process of endocytosis, the drug delivery system is engulfed in a vesicle derived from the plasma membrane. Once the vesicle is pinched off, it is referred to as early or sorting endosome and it is designated for cargo sorting. Herein, the cargo can either be recycled back to the plasma membrane for excretion or transported towards a lysosome via a late endosome for degradation.^[^ ^15^, ^88^ ^]^ Circumvention of lysosomal accumulation is often desirable for the therapeutic activity of cargo encapsulated in lipid nanoparticles. In contrast, nanocarriers may also rely on endo‐lysosomal trafficking to release their therapeutic cargo through endosomal disruption.^[^ ^207^ ^]^ In the following section, we discuss the intracellular trafficking of liposomes and EVs in relation to their internalization mechanism, which generally consists in the transport across the endo‐lysosomal system.

### 3.1 Endolysosomes: Comparison of Trafficking and Accumulation Sites of Liposomes and EVs

#### 3.1.1 Intracellular Trafficking of Liposomes

The vast majority of internalized liposomes are transported along the endo‐lysosomal pathway, irrespective of their uptake mechanism. Firstly, following caveolae‐mediated internalization of DiD‐labeled DOPC:DOPG:MPB‐PE (40:10:50 molar ratio) liposomes by HeLa cells, the nanoparticles were detected inside lysosomes. Only a small fraction was located in TGN38‐positive organelles, a representative for the trans‐Golgi complex. Regardless of the accumulation site of liposomes, encapsulated doxorubicin was released into the cytosol, where it was able to reach the nucleus 3 h post‐exposure.^[^ ^76^ ^]^ Similarly, Cy3‐labeled DOPE:cholesterol lipoplexes were colocalized with endo‐lysosomal compartments, indicated by early endosomes antigen 1 and LAMP‐1 markers, after clathrin‐dependent uptake by COS‐7 cells. Although lipoplexes accumulated in lysosomes, interfering with lysosomal degradation using lysosomotropic agents, such as chloroquine and bafilomycin, reduced the transfection potency of lipoplexes instead of enhancing it.^[^ ^78^ ^]^ This suggests that in this case, lysosomal accumulation was favorable for transfection. Likewise, DOTAP:DOPC (1:1 molar ratio) lipoplexes containing Cy3‐labeled DNA presented an overlap with organelles labeled with Lysosensor, corresponding to lysosomes, in CHO‐K1 cells after macropinocytotic uptake.^[^ ^112^ ^]^ Together, these findings suggest that lysosomal accumulation of lipid‐based nanocarriers takes place irrespective of the internalization mechanism.

Despite the transport of liposomes to lysosomes, in time the nanocarriers may continue the intracellular trafficking toward different organelles. Although after clathrin‐mediated endocytosis, DOTAP‐containing liposomes were located inside lysosomes at 1 h post‐exposure in HeLa cells, 24 h later the nanoparticles were found in mitochondria, endoplasmic reticulum, and trans‐Golgi complex.^[^ ^208^ ^]^ To unravel which lipid‐trafficking proteins were in play for intracellular trafficking, numerous proteins were suppressed through gene silencing and DOTAP was labeled with NBD to identify changes in the amount of intracellular lipid. Following the suppression of Niemann‐Pick C1 protein and oxysterol‐binding protein 1, the amount of intracellular phospholipid after exposure to DOTAP:DOPC (1:1 molar ratio) liposomes was increased. Niemann‐Pick C1 protein and oxysterol‐binding protein 1 are known for transporting lipid molecules to the cytoplasm from endosomes and lysosomes, respectively. Therefore these proteins were involved in the trafficking of lipoplexes from endo‐lysosomal compartments to the cytoplasm. Meanwhile, the amount of phospholipid remained unaltered with the knockdown of oxysterol‐binding protein 1 and exposure to DOTAP:cholesterol (1:1 molar ratio) liposomes. Hence, DOTAP:DOPC liposomes were transported to the cytoplasm from both endosomes and lysosomes, while those composed of DOTAP:cholesterol were transported only from the latter. After these nanocarriers reached the cytoplasm, they gained access to the mitochondria, endoplasmic reticulum, and trans‐Golgi complex. Furthermore, silencing of ceramide‐transfer protein and sec31A led to an increase in the intracellular phospholipid content. These proteins are known for their role in lipid molecule transport between the trans‐Golgi complex and endoplasmic reticulum. Therefore, ceramide‐transfer protein and sec31A were involved in the transport of liposomes between the trans‐Golgi complex and endoplasmic reticulum. Additionally, silencing of oxysterol‐binding protein 2 and phosphatidylinositol transfer protein increased the number of phospholipids after exposure to DOTAP:DOPC liposomes. In contrast, phosphatidylinositol transfer protein silencing had no effect after incubation with DOTAP:cholesterol liposomes. Phosphatidylinositol transfer protein is known for trafficking lipid molecules from trans‐Golgi complex and endoplasmic reticulum to the plasma membrane, whereas oxysterol‐binding protein 2 is involved in the transport from endoplasmic reticulum to plasma membrane. Therefore, DOTAP:DOPC liposomes were transported from both trans‐Golgi complex and endoplasmic reticulum to the plasma membrane, while those based on DOTAP:cholesterol were transported only from the trans‐Golgi complex. Subsequently, liposomes were extracellularly effluxed through ATP‐binding cassette transporters located in the plasma membrane.^[^ ^208^ ^]^ It should be noted that it is possible that after lysosomal accumulation the NBD‐labeled lipids dissociated from liposomes and the intracellular trafficking described thus far refers only to the trafficking of DOTAP alone and not the liposomes as a whole. Overall, lipid composition can to some extent influence the intracellular trafficking of liposomes and certain liposomes may localize in distinct cellular compartments even after lysosomal accumulation.

Still, trafficking of lipoplexes to lysosomes does not necessarily result in an unsuccessful transfection. Gilleron et al. studied the uptake, intracellular trafficking, and delivery of DLin‐MC3‐DMA:DSPC:cholesterol:DMG‐PEG (≈50:10:38.5:1.5 molar ratio) lipoplexes in HeLa cells.^[^ ^117^ ^]^ Analysis of intracellular trafficking revealed that 70% of the lipoplexes co‐localized with Rabankyrin‐5‐positive vesicles, early endosomes, and lysosomes. Subsequent analysis showed that ≈50% of these lysosomes co‐localized with Rabankyrin‐5‐positive vesicles and early endosomes. In comparison, the number of double co‐localized structures in cells not exposed to lipoplexes was negligible, arguing that these nanoparticles may trigger hybridization between early and late endocytic organelles. This implies that after clathrin‐mediated endocytosis and macropinocytosis, lipoplexes accumulate at the same site, namely lysosomes. Furthermore, electron microscopy showed that upon gold‐labeled siRNA encapsulation, only a minor fraction (1–2%) of siRNA was able to escape the endo‐lysosomal pathway. Despite the low cytosolic release, the GFP signal intensity of GFP‐expressing HeLa cells was reduced by ≈60–65% after exposure to 20 × 10^‐9^ m siRNA‐encapsulated lipoplexes. This suggests that even a minor amount of cytosolic delivery of siRNA can achieve efficient transfection.^[^ ^117^ ^]^ The inefficient endosomal escape of siRNA in HeLa cells was also confirmed in the case of L319:DSPC:cholesterol:DMG‐PEG (55:10:32.5:2.5 molar ratio) lipoplexes, in which the ionizable L319 lipid is a biodegradable derivative of the DLin‐MC3‐DMA lipid.^[^ ^209^ ^]^ During intracellular trafficking analysis, it was shown that siRNA release from lipoplexes occurred as early as 5–10 min after internalization, during endosome maturation into late endosomes. Only 3.5% of siRNA was found to be present in the cytosol after lipoplex‐mediated delivery. Nonetheless, approximately 55% of EGFP‐transfected HeLa cells showed a decrease in fluorescence intensity, after exposure to 20 × 10^‐9^ m of lipoplexes.^[^ ^209^ ^]^ These data further corroborate that transfection by lipoplexes can be achieved in spite of the reduced endosomal escape of encapsulated siRNA.

Aside from lysosomal degradation, poor cytosolic delivery of nucleic acids by lipoplexes can also be caused by exocytosis of the cargo.^[^ ^210^ ^]^ Following macropinocytotic uptake of C12‐200:DSPC:cholesterol:DMG‐mPEG_2000_ lipoplexes by HeLa cells, the nanocarriers showed low co‐localization with early endosomes 15 min after exposure. However, the lipoplexes kept accumulating inside lysosomes up to 1 h, after which this started to decrease. Meanwhile, co‐localization analysis of lipoplexes with transferrin and Rab11 domains, markers for recycling endocytic compartments, revealed that a small fraction of nanoparticles accumulated in recycling organelles in 2–3 h.^[^ ^210^ ^]^ Furthermore, by measuring the fluorescence signal of AF647‐labeled siRNA in the extracellular medium after lipoplex exposure, the authors found 2.5‐fold higher siRNA content in the extracellular milieu, compared to that inside cells. Nonetheless, the low amount of siRNA present in the cytosol exerted 50% transfection efficiency in cells. Together, these findings suggest that despite the exocytosis of the majority of nucleic acids, transfection can still be achieved. Further investigations revealed that the Niemann‐Pick type C1 glycoprotein, located on the surface of multivesicular late endosomes, is one of the factors contributing to siRNA recycling. Mouse embryonic fibroblasts deficient in this protein exhibited ≈15‐fold increase in siRNA accumulation in perinuclear vesicles, 24 h post‐exposure to lipoplexes. Moreover, enhanced gene silencing was present in these fibroblasts. The successful transfection in the absence of Niemann‐Pick type C1 glycoprotein is possibly due to the increased retention of lipoplexes in late endosomes and lysosomes, where the siRNA can slowly diffuse into the cytosol.^[^ ^210^ ^]^ Overall, transport across the endo‐lysosomal pathway is irrespective to the internalization mechanism of lipoplexes. Furthermore, only a small fraction of nucleic acids, encapsulated in lipoplexes, is able to escape lysosomal accumulation during endo‐lysosomal transport to arrive in the cytosol. Nonetheless, the cytosolic delivery of even a small amount of siRNA can exert a potent transfection.

Direct cytosolic release and enhanced transfection can also be achieved through cellular uptake of lipoplexes via membrane fusion, which in turn allows the lipid‐based nanocarrier to bypass the endo‐lysosomal pathways. Lu and co‐workers studied the uptake of commercially available DharmaFECT1 lipoplexes, carrying siRNA against cyclophilin B, in BSC‐40 African green monkey kidney epithelial cells.^[^ ^211^ ^]^ The authors showed that ≈95% of lipoplexes were internalized through clathrin‐, caveolae‐, lipid raft‐dependent pathways, and macropinocytosis, accumulating in endo‐lysosomal compartments over time. While lysosomal accumulation is expected after endocytosis, upon treatment with several inhibitors, the knockdown efficiency of the DharmaFECT1 transfection agent, against endogenous gene cyclophilin B, remained unaltered. Nonetheless, depletion of cholesterol using nystatin and filipin, completely abolished knockdown of the target transcript, despite having minimal effect on cellular uptake. This indicates that endocytosis is the primary uptake mechanism for these lipoplexes. However, the transfection potency of lipoplexes may have been dictated by a cholesterol‐dependent uptake mechanism, possibly membrane fusion, by which the cargo was delivered directly into the cytosol.^[^ ^211^ ^]^ Therefore, membrane fusion‐mediated uptake of liposomes can improve cytosolic delivery and the subsequent transfection potency of lipoplexes.

In conclusion, liposomes are carried across the endo‐lysosomal route, irrespective of their uptake mechanism, leading to their degradation. Only a small fraction of cargo is able to escape lysosomal accumulation during endosome maturation. Nonetheless, the minor fraction of nucleic acid inside the cytosol can exert transfection to a certain degree. Lysosomal escape can be potentiated by adjusting lipid composition of liposomes, such as adding fusogenic lipids. Still, internalization via membrane fusion is likely the most effective means of direct cytosolic delivery with an assured therapeutic effect.

#### 3.1.2 Intracellular Trafficking of EVs

Natural drug delivery systems share the same intracellular trafficking routes as their synthetic counterparts. EVs isolated from HEK293 cells exhibited slow lysosomal accumulation in human primary fibroblasts.^[^ ^212^ ^]^ After actin‐mediated uptake, EVs were engulfed in endocytic vesicles and shuttled in close proximity of the endoplasmic reticulum. Over the course of 2 d, approximately ≈50–60% of EVs accumulated inside lysosomes. Based on these data, it was hypothesized that EVs are transported towards the endoplasmic reticulum as a possible site of cargo release, followed by retrograde transport to lysosomes for degradation.^[^ ^212^ ^]^ Similarly, EVs derived from PC12 cells were transported to lysosomes after actin‐mediated uptake in recipient parental cells.^[^ ^213^, ^214^ ^]^ To reveal the intracellular fate of EVs and its content, dual labeling was carried out using DiD and amine‐reactive TAMRA‐NHS to distinguish between lipids and proteins, respectively. During intracellular trafficking, labeled EV proteins co‐localized with dextran, indicating lysosomal accumulation. Meanwhile, the lipophilic dye was located at the cell periphery, suggesting lipid recycling. This may be due to presence of sphingomyelin being an abundant EV‐membrane component, which was shown to possess recycling properties.^[^ ^213^, ^214^ ^]^ To stimulate endosomal escape, EVs can be engineered with endosomolytic peptides, such as L17E. U87 cells were genetically engineered to express bioluminescence via a miR21‐responsive luciferase reporter system and incubated with blood‐derived EVs containing L17E and miR‐21. Luciferase expression of the U87 cells incubated with miR‐21‐EVs alone was reduced to approximately 70%, while those treated with miR‐21‐EVs containing L17E were reduced by nearly 50%. Additionally, this engineered nanocarrier was loaded with doxorubicin and upon administration into tumor‐bearing mice, the chemotherapeutic activity reduced the size of the tumor more than two‐fold without any side effects, compared to control.^[^ ^215^ ^]^ Additionally, loading of EVs with endosomolytic peptides, such as L17E, not only enhanced the capability of nanocarriers to evade degradation, but greatly improved the efficacy of chemotherapeutics.

Lysosomal accumulation of drug delivery systems is even more prominent in immune cells, as they fulfill a role in eliminating foreign entities by degradation. Following phagocytosis of K562 or MT4‐derived EVs by RAW264.7 macrophages, the nanoparticles were transported via phagosomes to lysosomes, subsequently giving birth to a hybrid organelle, called phago‐lysosomes. Confocal images showed 49%, 38%, and 66% co‐localization between EVs and lyso‐bis‐phosphatidic acid, Rab7, and lysosomal associated membrane protein‐1 (LAMP‐1), respectively. This indicated that phagosomes fused with lysosomes.^[^ ^143^ ^]^ Even though phagocytosis is the main uptake mechanism in immune cells, membrane fusion has also been reported as an uptake mechanism (see Section 2.2.), with dendritic cells shuttling EVs between one another as a form of communication.^[^ ^202^ ^]^

Although lysosomal accumulation is a frequently occurring event, a new intracellular trafficking route was established for endocytosed EVs: a pathway from late endosomes to the nucleoplasmic reticulum.^[^ ^216^ ^]^ Rappa and co‐workers reported that a limited amount of human primary MSCs‐, malignant melanoma FEMX‐I‐ or MDA‐MB‐231 cell‐derived EVs are trafficked in their autologous recipient cells to sub‐nuclear compartments via late endosomes containing Rab7 subdomains.^[^ ^217^ ^]^ To visualize intracellular trafficking, FEMX‐I‐, MDA‐MB‐231 cells, and primary MSCs were engineered to express GFP‐CD9 fusion proteins to produce fluorescent GFP‐CD9 EVs for isolation. Subsequently, the inner nuclear membrane of cells was tagged with antibody against SUN domain‐containing protein 2, a marker of nuclear envelope invaginations. Confocal laser‐scanning microscopy images showed GFP signals within the inner nuclear membrane of FEMX‐I cells and MSCs, after exposure to autologous EVs, indicating that EV content was transferred to the nuclei. Furthermore, labeling of EVs with DiI prior to their incubation with native FEMX‐I cells demonstrated through the presence of DiI fluorescence signal within the nuclei that, along with proteins, EV membrane fragments were also transferred to the nucleoplasm. To investigate the intracellular route of EVs, stromal and cancer cells were infected with baculovirus to produce RFP‐Rab7 to identify late endosomes. Microscopy images showed an overlap between RFP‐Rab7 and GFP‐CD9 signals, surrounded by SUN domain‐containing protein 2. This indicates that late endosomal Rab7 subdomains come together with nuclear envelope invaginations, thereby generating a sub‐nuclear compartment, allowing EVs to deliver their content into the nucleoplasm. One of the factors within EVs, identified as pivotal for their nucleoplasmic trafficking is importin β1, of which presence in EVs was confirmed by proteomic analysis. This molecule is known for mediating nuclear transport of cytoplasmic proteins via nuclear pores. As such, treatment of cells with importazole, an inhibitor of importin β1 function, abolished the nuclear localization of GFP‐CD9 EVs, therefore confirming its role in the nuclear transport of EVs.^[^ ^217^ ^]^

Intriguingly, this intracellular trafficking pathway allowed EVs, isolated from FEMX‐I cells, to transfer their nucleic acid content into the nuclei of MSCs, consequently affecting gene expression. RNA sequencing revealed that 11 out of 15 genes were altered due to the nuclear translocation EVs. These genes included ghrelin, interleukin‐26, −17B, Casp1, and CCL5, known to be involved in inflammatory processes.^[^ ^217^ ^]^ Read et al. also corroborated the nuclear transfer abilities of EVs, specifically membrane receptors, such as EGFR and androgen receptors, and its mutant variants, in cells devoid of these receptors.^[^ ^218^ ^]^ Nuclear delivery of EV content was demonstrated using EVs isolated from cutaneous carcinoma A431 cells that inherently overexpress wild‐type EGFR and CHO‐K1 cells that naturally lack these receptors. After incubation with EVs, cells were fractionated and the nuclear and cytoplasmic extracts were immunoblotted for EGFR levels. Analysis of the nuclear fraction confirmed that CHO‐K1 cells treated with A431‐derived EVs were positive for EGFR, indicating nuclear transport of EV content. Similarly, immunoblotting confirmed that prostate cancer LNCaP cell‐derived EVs containing androgen receptors transferred its content into PC‐3 cells, lacking the receptors. The nuclear transfer of androgen receptors activated the transcription of androgen receptor‐responsive genes. Furthermore, the nucleic transfer of mutant androgen receptor v7 in LNCaP cells led to an enhanced cell proliferation rate, similar to that of cells stimulated with dihydrotestosterone.^[^ ^218^ ^]^ These findings show the importance of understanding the relation between uptake and the pro‐tumorigenic activity of cancerous EVs resulted from internalization. Overall, the intracellular trafficking of EVs is similar to that of liposomes, which is to follow the endo‐lysosomal pathway. Furthermore, as a small fraction of nucleic acid is able to reach the nucleus through lipoplex delivery, so does the nucleic content of EVs.

Thus far, we have discussed the similarities between liposomes and EVs in terms of their tendency to accumulate in lysosomes as a final destination. However, recently a comparative study between liposomes and lipid:EV hybrids revealed notable differences in terms of internalization mechanism and intracellular trafficking.^[^ ^172^ ^]^ Sk‐hep1 cells were exposed to pharmacological inhibitors, namely chlorpromazine, filipin, and amiloride. DPPC liposomes were found to enter cells exclusively via caveolae‐mediated endocytosis, whereas DPPC:EVs internalized through clathrin‐, caveolae‐mediated endocytosis, and macropinocytosis. To investigate their intracellular accumulation, Sk‐hep1 cells were stained against LAMP1, Golgin‐97, and KDEL for lysosomes, the trans‐Golgi complex, and endoplasmic reticulum, respectively. Confocal microscopy revealed approximately 40% co‐localization between DPPC:EVs and the trans‐Golgi complex, which is 6.7‐fold higher than that of DPPC liposomes. Approximately 60% co‐localization was observed between DPPC:EVs and the endoplasmic reticulum, which was 2.2‐fold higher than that of DPPC liposomes. Additionally, DPPC liposomes also showed a 37.3% co‐localization with lysosomes. This ability of DPPC:EVs to circumvent lysosomal accumulation increased their gene silencing efficiency compared to that of DPPC liposomes. Gene transfection efficiency of these vesicles was assessed using cyclin‐dependent kinase 1 (CDK1)‐siRNA as a model system. Following exposure to siCDK1‐DPPC:EVs, the mRNA levels of CDK1 in Sk‐hep1 cells exhibited an 86.7% reduction compared to controls, which was 1.7‐fold more efficient than siCDK1‐DPPC liposomes.^[^ ^172^ ^]^ It has been reported previously that effective suppression of the CDK1 gene in c‐Myc‐overexpressing HCC cells is lethal, leading to cancer cell growth inhibition.^[^ ^219^ ^]^ This was corroborated by the flow cytometry analysis in which Sk‐hep1 cell exposure to siCDK1‐DPPC:EVs resulted in 80.5% apoptotic efficiency, approximately twice as high as those of siCDK1‐DPPC liposomes. Similar silencing efficiencies were reported during in vivo investigations, which resulted in 33% of the mice receiving siCDK1‐DPPC:EVs with complete tumor regression.^[^ ^172^ ^]^

#### 3.1.3 Accumulation of Liposomes and EVs in Lysosomes: Desirable or Undesirable?

So far, we discussed the shuttling of liposomes and EVs across the endo‐lysosomal pathway and occurrences in which these lipid‐based nanocarriers circumvent lysosomal degradation, ending up in different cellular compartments. In the section below, we will explore situations in which lysosomal accumulation is favorable. For instance, the endo‐lysosomal pathway could be exploited to induce immune responses against viruses and tumors by taking up drug delivery systems carrying relevant antigens. Immune responses rely heavily on the presentation of antigens by antigen‐presenting cells to T cells. The antigen needs to be internalized and delivered to endo‐lysosomal compartments within antigen‐presenting cells for processing. Here, the generated peptides can be loaded onto MHC proteins and subsequently transported to the surface of cells, a process also known as cross‐presentation.^[^ ^220^, ^221^ ^]^ EVs naturally contain antigens that could be exploited for this purpose. As such, Morelli and co‐workers investigated the intracellular transport and processing of EVs by dendritic cells.^[^ ^222^ ^]^ Within 5 min after uptake, autologous EVs were found to co‐localize with transferrin, indicating clathrin‐mediated endocytosis. After 2 h, EVs co‐localized with low‐density lipoproteins and LAMP‐1, recognized as late endosome and lysosome markers, respectively. By blocking acidification of endocytic vacuoles using ammonium chloride treatment, the capacity of dendritic cells to present EV‐derived allopeptides—peptides that play a role in distinguishing its own tissues from those of another organism—was impaired. This indicated that EVs traveled across the endo‐lysosomal pathway, where EV‐derived allopeptides were loaded onto MHC molecules for presentation to T cells. These results were confirmed in vivo, in which EVs were administered in mice.^[^ ^222^ ^]^ However, not all lysosomal accumulation of EVs in immune cells will trigger an immune response. This depends on a number of factors, one of which is the recipient cell type. For example, no immunological response was triggered upon selective uptake of oligodendroglia‐derived EVs by a subpopulation of unstimulated and MHC class II‐negative microglia cells, despite their accumulation in lysosomes.^[^ ^114^ ^]^ Stimulation of an immune response is also dependent on the type of CD8^+^ or CD4^+^ T cells of interest, for which antigens are presented by MHC class I or II, respectively.^[^ ^223^ ^]^ The processing of antigens by MHC class I or II could be related to their intracellular trafficking, which in turn depends on the lipid composition. For example, Harding et al. showed that ovalbumin encapsulated in acid‐resistant DOPC:DOPS (4:1 molar ratio) liposomes was processed by MHC class II in macrophages, only after accumulation in lysosomes. Instead, ovalbumin delivered by acid‐sensitive DOPE:PHC (4:1 molar ratio) liposomes was processed by MHC class I after cytosolic release from endosomes. This example shows that lipid composition may dictate intracellular delivery and subsequent processing by MHC, which in turn mediates the level of immunological response.^[^ ^221^ ^]^

In the majority of cases, lysosomal accumulation remains unfavorable for therapeutic molecules that have a different action site, such as the nucleus, endoplasmic reticulum or mitochondria. To circumvent cargo degradation, one approach is to trigger cytosolic release of encapsulated cargo by exploiting the pH at the intended site of release. Confocal imaging showed that EVs tagged with a fusion protein of GFP and Hsp‐70 co‐localized with endo‐lysosomal markers, such as Rab5 for early endosomes and lysosome‐associated membrane protein 1 for lysosomes.^[^ ^224^ ^]^ Furthermore, to confirm the presence of NanoLuc luciferase‐tagged Hsp70 in the cytosol, cells were fractionated by separating the cytosolic from the membranous fraction. By measuring luciferase activity, the signal located in the cytosolic fraction was shown to amount to roughly 20–30% of the internalized EVs releasing their content into the cytosol. Additionally, the cells were treated with bafilomycin A1 to inhibit endosome acidification and less signal was observed in the cytosolic fractions, which led to the conclusion that acidic pH is pivotal for cytosolic release.^[^ ^224^ ^]^ Another study conducted by Joshi and co‐workers also confirmed that acidification triggers cytosolic cargo release of EVs.^[^ ^225^ ^]^ In this regard, the N‐terminal of CD63 located on the cytosolic site of HEK293T cell‐derived EV was conjugated with GFP. In addition, HEK293T cells were engineered to express anti‐GFP nanobodies fused with mCherry. In case of endo‐lysosomal accumulation and EV‐endosome membrane fusion, the GFP‐CD63 would be exposed to the cytoplasm where anti‐GFP nanobodies are able to recognize this cargo and form double‐positive punctae of GFP/mCherry. Fluorescence microscopy of engineered HEK293T cells incubated with GFP‐CD63‐EVs confirmed the formation of double‐positive punctae in approximately 95% of the cells, indicating that in the majority of cells EVs fused with endosomal compartments. Furthermore, upon inhibition of acidification with bafilomycin A1 treatment, the formation of double‐positive punctae was completely abolished, corroborating membrane fusion of EVs during endo‐lysosomal trafficking.^[^ ^225^ ^]^

A different approach to escape lysosomal degradation is to insert fusogenic lipids into liposomes to promote cytosolic release by interaction with the endo‐lysosomal membrane.^[^ ^77^, ^226^, ^227^, ^228^, ^229^ ^]^ When these liposomes are in proximity to an endo‐lysosomal compartment, the cationic moiety of these fusogenic lipids interacts with the anionic endosomal membrane. This interaction leads to a mixing between the lipids of the nanocarrier and those of endosomal membrane, subsequently releasing the cargo into the cytosol.^[^ ^230^, ^231^ ^]^ Khalil and co‐workers showed that by changing the lipid envelope of R8‐conjugated non‐fusogenic EPC:cholesterol (7:3 molar ratio) lipoplexes to fusogenic DOPE:cholesteryl hemisuccinate (9:2 molar ratio) ones increases cytosolic release.^[^ ^118^ ^]^ After encapsulating plasmid DNA encoding a luciferase reporter gene in both types of lipoplexes and exposing these nanoparticles to NIH3T3 cells, the luciferase activity was measured in cell lysate. The luciferase activity of fusogenic lipoplexes was two to three orders of magnitude higher than their non‐fusogenic counterparts, confirming efficient cytosolic release by using fusogenic lipids.^[^ ^118^ ^]^ In another study, Jiang et al. used plasmid‐laden chitosan nanoparticles, which were coated with PC‐98T:cholesterol (4:1 w/w), followed by post‐insertion of DOTAP lipids (total lipid:DOTAP = 6:1 w/w). The latter was shown to enhance the gene transfection ability of liposomes after lysosomal accumulation. Aside from stimulating multiple endocytosis mechanisms, DOTAP insertion aided in lysosomal escape and increased GFP expression by approximately threefold.^[^ ^232^ ^]^

Collectively, natural and synthetic drug delivery systems are typically destined for lysosomal accumulation, which can lead to degradation depending on the lipid composition. To circumvent this intracellular pathway, tweaking the lipid composition by altering the lipid formulation of liposomes or by varying parent cell origin of EVs, may represent an elegant solution to target different cellular compartments.

### 3.2 Controlling the Intracellular Accumulation Sites of Liposomes and EVs

To enhance the desired therapeutic effects, researchers have focused on targeting lipid‐based drug delivery systems toward specific organelles. In this section, we will explore how functionalization of drug delivery vesicles affects the transport inside cells in relation to their uptake mechanism.

#### 3.2.1 Dictating the Final Fate of Engineered Drug Delivery Vesicles

The efficiency of therapeutic delivery heavily relies on its ability to accumulate at the desired site of action. By targeting receptors of which internalization mechanisms and trafficking are known, the intracellular fate of drug delivery systems may be modulated and therapeutic efficacy enhanced. This was evidenced in the case of liposomes. In a study by Arta and co‐workers the cellular internalization mechanism and the intracellular trafficking route of DPPC:cholesterol:DSPE‐PEG_2000_:maleimide liposomes differed in primary human retinal endothelial cells depending on the decorating agents of these nanocarriers.^[^ ^233^ ^]^ The cationic liposomal formulation DPPC:DSTAP:Cholesterol:DSPE‐PEG_2000_:DiO (25.9:30:40:4:0.1 molar ratio) was internalized via clathrin‐mediated endocytosis, which led to lysosomal accumulation. Their anionic counterparts, i.e., (DPPC:cholesterol:DSPE‐PEG_2000_:maleimide:DiO with a 55.9:40:3:1:0.1 molar ratio), lacking DSTAP and functionalized to target the endothelial protein C receptor, had a similar uptake mechanism, yet it was equally distributed between early‐, late‐, recycling endosomes, and lysosomes. The same anionic formulation, but functionalized with transferrin, internalized only via clathrin‐mediated entry and accumulated mainly in recycling endosomes.^[^ ^233^ ^]^ This indicates that the uptake mechanism is not connected to intracellular fate and targeting moieties may alter the entry route and subsequent intracellular trafficking of liposomes. Nonetheless, differences in lipid composition should also be taken into account.

Enhancing the internalization of EVs draws inspiration from biological entities, such as viruses, and their approach to induce internalization. Temchura and co‐workers decorated the surface of EVs with a pH‐responsive viral fusion protein derived from vesicular stomatitis virus G‐proteins (VSV‐G), with the aim to increase cross‐presentation of ovalbumin for immunotherapy.^[^ ^234^ ^]^ Engineered EVs showed enhanced uptake by dendritic cells, possibly due to the increased association of EVs with the cell membrane. This association facilitated cellular entry of EVs into dendritic cells. Thereafter, the nanocarriers accumulated in phago‐lysosomal compartments, where the acidic environment caused cytosolic release of ovalbumin, allowing MHC class I presentation of the antigen.^[^ ^234^ ^]^ A similar approach was exploited in cancer immunotherapy research, where the surface of macrophage‐derived EVs was decorated with VSV‐G to enhance the therapeutic efficacy of encapsulated siRNA to block the programmed death‐ligand 1 pathway.^[^ ^104^ ^]^ Fusion of VSV‐G‐EVs with CT26 murine colorectal carcinoma cells was found to facilitate direct release of Cy5‐labeled siRNA into the cytoplasm, triggering effective gene silencing. Fusion activity of VSV‐G decorated EVs was confirmed by the quantitative analysis of CypHer5 cyanine dye present in cells after delivery via EVs at different pH conditions (7.4 and 6.5). As VSV‐G is a pH‐sensitive protein, 56% of the cells incubated with CypHer5‐labeled EVs decorated with VSV‐G exhibited fluorescence signals at pH 6.5, while only ≈10% of the cells had a signal at pH 7.4. This suggests that cargo delivery occurs in a pH‐dependent manner via membrane fusion and fusogenic proteins of viral origin can be exploited for direct cytosolic delivery of therapeutics.^[^ ^104^ ^]^ In agreement with these findings, Yang and co‐workers showed that VSV‐G engineered EVs fused with the cell membrane of HEK293T cells under acidic conditions, whereas at neutral pH the engineered EVs were internalized through endocytosis as demonstrated through co‐localization analysis.^[^ ^235^ ^]^ However, nucleic acid delivery and transfection efficiency of VSV‐G engineered EVs were found to be similar to that of Lipofectamine 2000, making the latter potentially more practical in terms of production.^[^ ^104^ ^]^ Flow cytometry analysis revealed that 73–75% of cells contained Cy5‐siRNA after Lipofectamine 2000 or EV treatment. Meanwhile 54–58% of cells presented mRNA knockdown of programmed death‐ligand 1, despite the differences in intracellular trafficking. Following co‐localization analysis, Cy5‐siRNA accumulated inside endo‐lysosomal compartments after Lipofectamine 2000 delivery, compared to their cytosolic presence upon membrane fusion of VSV‐G engineered EVs. However, there were significant differences in cytotoxicity level, where Lipofectamine 2000 loaded with 800 × 10^‐9^ m siRNA presented 41% cell viability, compared to that of EVs.^[^ ^104^ ^]^ Although VSV‐G engineered EVs have similar uptake and gene delivery efficiencies as Lipofectamine 2000, based on the cytotoxicity levels, EVs are deemed safer for therapeutic applications in this setting.

Fusogenic peptides of non‐viral origin, such as GALA, have also been designed to deliver cargo directly into the cytosol in acidic environments. Nakase et al. showed that cellular uptake and cytotoxic effects of saporin‐encapsulated EVs were greatly improved by the addition of cationic lipids, such as Lipofectamine LTX, and by the incorporation of GALA peptides.^[^ ^236^ ^]^ The cationic lipid acted as a “glue” between the negatively charged EV and plasma membrane, leading to enhanced cellular uptake. After internalization, the acidic environment of the endosome activated the fusogenic properties of GALA peptides, causing endosomal disruption and successful cytosolic release. Interestingly, the uptake of unmodified EVs was completely abolished in the absence of cationic lipids, as well as the cytotoxic effects of therapeutic cargo in the absence of GALA peptides.^[^ ^236^ ^]^ As described in Section 2.2 on membrane fusion, a comparable method made use of complementary coiled‐coil lipopeptides for direct membrane fusion of nanoparticles to ensure direct cytosolic release. As such, Yang et al. conjugated the coiled‐coil forming peptide (EIAALEK)_3_ to liposomes through a cholesterol moiety and a PEG spacer. These engineered liposomes demonstrated direct membrane fusion abilities with (KIAALKE)_3_ peptide‐decorated HeLa cell membranes. As only a small fraction of the engineered liposomes was co‐localized with endosomes, it was concluded that the liposomal cargo was directly delivered into the cytosol of cells.^[^ ^126^ ^]^ Interestingly, the same functionalization on EPC:cholesterol liposomes triggered different internalization mechanism, namely that of clathrin‐mediated endocytosis.^[^ ^237^ ^]^ These differences might be caused by the lipid composition or presence or absence of surface proteins. Overall, these data suggest that fusogenic and coiled‐coil peptides are a useful tool to trigger cytosolic release of cargo via membrane fusion. However, the uptake mechanism may be influenced by the lipid composition of the drug delivery system or by the recipient cell type.

Decoration of the drug delivery systems’ surface with PEG is commonly used to prolong blood circulation and to shield nanoparticles from the reticuloendothelial system.^[^ ^39^ ^]^ However, the presence of PEG appears to promote a reduction in cellular uptake and an intracellular trafficking that leads to lysosomal degradation.^[^ ^238^, ^239^ ^]^ Remaut and co‐workers showed that the insertion of 5 mol% DSPE‐PEG in oligonucleotide containing DOTAP:DOPE (1:1 molar ratio) liposomes prevented destabilization of endosomal compartments, which ultimately targeted these nanocarriers to lysosomes. Oppositely, DOTAP:DOPE liposomes lacking PEG promoted endosomal escape.^[^ ^239^ ^]^ High‐density surface decoration with PEG was also shown to inhibit cellular internalization of drug delivery systems and shield‐targeting molecules.^[^ ^240^ ^]^ For example, insertion of DPPE‐PEG2000 into DPPC:DOPE:DC‐cholesterol:DHPE (1.3:1:1:0.0003 molar ratio) liposomes interfered with both uptake and endosomal escape in GBM cell lines. Confocal microscopy revealed that insertion of PEG halted the release of the encapsulated propidium iodide from liposomes. In comparison, cargo from non‐PEGylated liposomes was successfully released, as shown by the homogenous distribution of propidium iodide throughout the cytosol.^[^ ^241^ ^]^ Furthermore, PEGylation of liposomes has also been associated with reduced cellular uptake and decreased lysosomal escape of encapsulated plasmid DNA.^[^ ^242^, ^243^ ^]^ For example, PEG functionalization of Antennapedia‐decorated SPC:cholesterol:D,L α‐tocopherol liposomes led to lower binding efficiency and lower cellular uptake.^[^ ^151^ ^]^ A recent study conducted by Shen and co‐workers demonstrated that the reduced cellular uptake of PEGylated liposomes is caused by the aggregation of PEG polymers during internalization.^[^ ^242^ ^]^ This leads to ligand‐free regions on the surface of liposomes and causes a penalty within the free energy of approximately 800k_B_T, which is more than twice of the membrane bending energy necessary for internalization. The limited number of available ligands and the resulted energy barrier halts proper wrapping of the membrane during endocytosis and therefore reduces their chance of internalization by cells.^[^ ^242^ ^]^

In order to circumvent these issues, Fu et al. synthesized DSPE:SPC:cholesterol liposomes decorated with cleavable PEG and TAT cell‐penetrating peptides and encapsulated paclitaxel to reduce tumor growth.^[^ ^244^ ^]^ Murine B16F1 melanoma tumor cells were incubated with these liposomes in the presence of the reducing agent glutathione to cleave PEG and expose the TAT peptides. Cleavage of PEG resulted in enhanced cellular uptake and successful escape from lysosomal degradation. Incubation with several endocytosis inhibitors, such as sodium azide, monensin, cytochalasin D, and filipin led to a significant decrease in cellular uptake, suggesting caveolae‐mediated endocytosis and macropinocytosis as the main cellular entry mechanisms.^[^ ^244^ ^]^ An alternative to decoration with cleavable PEG is functionalization with pH‐sensitive PEG_2000_, which enhanced the transfection efficiency of DOTAP:DOPC (various molar ratios) lipoplexes in mouse fibroblast, compared to those functionalized with acid‐stable PEG_2000_.^[^ ^245^ ^]^

In contrast, PEGylation of EVs has largely benefited their uptake efficiency by targeted cells.^[^ ^246^, ^247^ ^]^ Unfortunately, research on the influence of EV PEGylation on cellular uptake and intracellular trafficking is very limited. Overall, although PEG is widely used to increase circulation time of drug delivery systems by circumventing immune surveillance, as well as to improve stability, it raises major concerns in overcoming several cellular barriers. These barriers are mainly cell association and circumvention of degradation. Nonetheless, the substitution of PEG with cleavable PEG showed promising results in overcoming those obstacles.

In summary, although liposomes and EVs are destined for enzymatic degradation, engineering with different moieties represents a useful tool to alter their fate. Functionalization with VSV‐G successfully evaded lysosomal accumulation of both liposomes and EVs and consequently delivered cargo directly into the cytosol, subsequently increasing therapeutic efficacy. Similar to VSV‐G, GALA peptides were also able to induce membrane fusion to facilitate cytosolic release of the cargo. Hence, functionalization of drug delivery systems represents a useful tool to dictate the intracellular trafficking of liposomes and EVs.

#### 3.2.2 Surface Functionalization of Liposomes to Promote Lysosomal Escape

Endosomal escape of drug delivery systems to prevent degradation of cargo is an active area of research. In this section, we will address different functionalization strategies of liposomes that ultimately circumvent degradation. For example, Ahmed et al. modified DOPC:DOPE (1:1 molar ratio) liposomes with hydrophobic polyampholytes.^[^ ^248^ ^]^ This functionalization allowed DOPC:DOPE liposomes to be taken up via macropinocytosis by mouse fibroblasts L929 cells, in addition to caveolae‐mediated uptake. Conjugation with polyampholytes and stimulation of macropinocytotic entry potentiated both endosomal escape and cytosolic release of lysozymes, used as a model protein, upon liposomal delivery to the cells.^[^ ^248^ ^]^ Similarly, high‐density R8‐decorated MENDs, based on DOPE:CHEMS (9:2 molar ratio) lipid composition, efficiently released their cargo into the cytosol of HeLa cells.^[^ ^120^ ^]^ After macropinocytotic entry of liposomes, AF546‐labeled siRNA was partly transported near and inside the nucleus, separately from the NBD‐dye labeled DOPE, 1 h post‐exposure. The subsequent increased transfection efficiency was attributed to the successful release of siRNA from the lipid envelope and to the translocation from macropinosomes to the cytosol.^[^ ^120^ ^]^ In comparison, high‐density R8‐MEND composed of EPC:CHEMS (9:2 molar ratio) liposomes exhibited a different intracellular fate. The stearyl R8‐functionalized GFP delivered by these liposomes was located in lysosomal compartments 6 h postexposure, while those encapsulated by DOPE:CHEMS liposomes were located in the cytosol suggesting endosomal destabilization by the fusogenic liposome.^[^ ^121^ ^]^ The presence of cationic lipid within the composition of liposomes is indispensable for endosomal destabilization, as these lipids engage with the anionic ones within the endosomes’ membrane, causing a flip‐flop motion from the outer to the inner face, thereby allowing the cargo to be displaced into the cytosol.^[^ ^39^, ^77^ ^]^

Another strategy to avoid degradation is by targeting specific cellular components using tailored lipid compositions. Various neurodegenerative diseases, such as Alzheimer's and Parkinson's disease are associated with mitochondrial dysfunction.^[^ ^249^ ^]^ As such, active targeting of drug delivery systems to mitochondria could be an effective therapeutic approach to address these neurodegenerative diseases. Yamada and co‐workers screened different liposomal formulations and evaluated their fusogenicity with isolated rat liver mitochondria and analyzed the intracellular delivery of GFP‐encapsulated liposomes in HeLa cells.^[^ ^250^ ^]^ The highest mitochondrial membrane fusion activities were reported for DOPE liposomes containing sphingomyelin or phosphatidic acid (9:2 molar ratio), formulations also referred to as MITO‐Porters. In the same study, confocal microscopy revealed that approximately 10% of HeLa cells internalized GFP‐encapsulated MITO‐Porters. After uptake, MITO‐porters co‐localized with mitochondria. Furthermore, surface conjugation of MITO‐porters, with high‐density R8 peptide, stimulated both uptake through macropinocytosis and successful mitochondrial membrane fusion through electrostatic interaction in HeLa cells.^[^ ^250^ ^]^

In addition to targeting mitochondria, targeting other organelles, such as the endoplasmic reticulum, could offer therapeutic potential in endoplasmic reticulum‐budding viruses, e.g., hepatitis C and bovine viral diarrhea virus. In this regard, Pollock and co‐workers studied the active delivery of iminosugars to endoplasmic reticulum using liposomes in the context of viral interference.^[^ ^251^ ^]^ In order to do so, the liposome composition was designed to resemble that of the endoplasmic reticulum membrane, namely PE:PC:PI:PS (1.5:1.5:1:1 molar ratio). Shortly after incubation with Huh7.5 cells, liposomes were located in early endosomal compartments, followed by co‐localization with trans‐Golgi complex and the endoplasmic reticulum, 20 min post‐exposure. After 22–25 min post‐incubation, liposomes fused with the membrane of endoplasmic reticulum. The authors also tested different liposomal formulations to examine differences in co‐localization levels. However, the PE:PC:PI:PS formulation presented the highest co‐localization (88%) with targeted endoplasmic reticulum of all formulations tested. As such, it appears that the presence of PI and/or PS increases liposome transport to the endoplasmic reticulum.^[^ ^251^ ^]^

Aside from tweaking lipid composition, surface functionalization with various moieties represents another method to successfully evade degradation, while targeting specific pathways. Conjugation with tosyl groups is deemed useful to actively target delivery of liposomes to endoplasmic reticulum. Following a 24 h incubation period of tosyl‐conjugated 17AAG‐PC:DSPE‐PEG_2000_ liposomes with HeLa cells, the nanocarriers co‐localized 59.2% with the endoplasmic reticulum. In addition, a small fraction of 21.5% and 35.3% were also detected in lysosomes and mitochondria, respectively.^[^ ^252^ ^]^

All together these results suggest that active targeting of organelles is possible, consequently circumventing degradation. Yet, there is still room for improvement of efficiency. To the best of our knowledge, there are no studies that reported surface functionalization of EVs to promote lysosomal circumvention, this area remains to be explored and the comparison between EVs and liposomes made.

## 4 Conclusion and Future Perspectives

In this review, we explored the underlying mechanisms of uptake of liposomes and EVs in target cells and the subsequent intracellular trafficking routes and accumulation sites, making a comparison between synthetic and natural origin. The classical endocytosis mechanisms involved in the internalization of these lipid nanoparticles are predominantly clathrin‐, caveolae‐, lipid raft‐, and HSPG‐dependent entry routes, and macropinocytosis with no clear preference for uptake of liposomes or EVs. Meanwhile, membrane fusion events appear to contribute less to the uptake of both types of drug delivery systems. Furthermore, the endocytic process of phagocytosis occurs mainly in specialized immune cells. The contribution of several endocytic routes to the internalization of liposomes and EVs, especially those independent of clathrin and caveolin, remains to be fully elucidated. Importantly, in the majority of cases, internalization does not rely on a single uptake mechanism. Instead, multiple mechanisms can contribute for both liposomes and EVs. This makes the identification of the exact uptake mechanism(s) challenging with the tools currently available. Furthermore, establishing similarities and differences between liposomes and EVs is complicated due to the differences in experimental design across studies. These include lipid formulations, absence or presence of serum in the experiment's culture, pharmacological inhibitor(s) used, exposure times, and recipient cell type tested. Additionally, in contrast to liposomes, EVs inherently express membrane surface proteins and intraluminal factors, which may influence their recognition by cells, consequently altering their internalization mechanism. However, so far, studies performing a head‐to‐head comparison between liposomes and EVs are scarce, making it challenging to assess whether these differences in composition are reflected in different uptake mechanisms and efficiencies. Results may be influenced by the lipid composition of the liposomes, the cell type used to produce EVs and the procedures employed to isolate them, the recipient cell type, and various other experimental parameters. Altogether, to compare uptake of liposomes and EVs across studies remains challenging due to the varying conditions. Conducting comparative studies in the future will be necessary to explore this topic in depth.

The intracellular trafficking route of liposomes and EVs generally follows the endo‐lysosomal pathway, accumulating inside lysosomes and undergoing degradation by hydrolytic enzymes, irrespective of the endocytosis mechanism. Although clathrin‐mediated endocytosis is believed to be the prevalent mechanism leading to lysosomal accumulation, in this review we have established that the majority of endocytosis mechanisms lead to the same fate for liposomes and EVs. Membrane fusion is the only internalization mechanism that can efficiently deliver the therapeutic cargo directly into the cytosol after internalization, circumventing degradation. Furthermore, macropinocytotic uptake may provide a higher chance of achieving cytosolic release, since the generated macropinosomes are leakier organelles than other endocytic vesicles. One of the main strategies to circumvent lysosomal accumulation is to actively target specific organelles based on the site of interest by conjugation with targeting moieties. Another is to tweak lipid composition by the incorporation of fusogenic lipids, such as DOPE, thus leading to endosomal destabilization. In this respect, liposomes may be more favorable nanocarriers than EVs, as they offer more control and flexibility in terms of changing lipid composition. Still, in general liposomes and EVs have largely the same fate within cells after uptake. Furthermore, liposomes have been studied for a longer period of time, also in the context of achieving a better performance by exploiting functionalization, whereas EVs have not yet been so extensively studied. Another possible outcome following endocytosis is accumulation in sorting endosomes after which the internalized lipid nanoparticles are recycled back to the plasma membrane and exocytosed from recipient cells, as shown in the case of liposomes. To date, this vesicle recycling has been underexplored even though recent evidence suggests that it could be a major contributor in unlocking the full potential of liposomes and EVs for drug delivery. Furthermore, late endosome‐nucleoplasmic reticulum transport is another research area, which should receive more attention to improve gene therapy of lipid‐based vesicles.

The techniques used to study the entry and intracellular shuttling of lipid nanoparticles, including labeling of vesicles with fluorescent probes, interference with uptake pathways using pharmacological inhibitors, siRNA‐mediated knockdown of specific proteins, and co‐localization analysis with established endocytic markers, are vital in elucidating the biomolecular machinery by which lipid nanocarriers are processed. Yet, there are several factors to keep in mind by using these tools for biological investigation. Fluorescent labeling of liposomes and EVs may alter their surface composition, thereby interfering or changing the cellular recognition of these entities. Therefore, it may result in different outcomes for in vitro and in vivo assessment. Another factor that should be taken into consideration is that fluorescent dyes could detach from or may even outlast lipid‐based vesicles, leading to false‐positive results. Alternatively, genetically transfected cells to express fluorescent signals may be used for EV labeling in order to circumvent membrane surface alteration with labeling dyes. Similarly, pharmacological inhibitors may also represent a poor reflection of internalization processes that would take place under natural conditions. This strategy may perturb the cellular machinery and trigger compensatory mechanisms for the uptake of drug delivery systems. This could also apply to siRNA‐mediated knockdown of specific endocytic proteins. Additionally, the currently available pharmacological inhibitors for endocytosis lack specificity in targeting a single uptake pathway, potentially leading to data misinterpretation. A few studies have shown that interference with specific endocytic proteins by treatment with pharmacological inhibitors increased the uptake of lipid‐based nanocarriers. However, siRNA‐mediated knockdown of those proteins did not have any effect on internalization, underlining the perturbation abilities of pharmacological inhibitors and risk of misinterpretation. In our view, several factors need consideration when studying uptake of lipid‐based nanoparticles. First, it is important to establish the appropriate concentrations for treatment with inhibitors by conducting cell viability assays. Secondly, positive controls for uptake mechanisms should be in place, including well‐established endocytic markers, such as transferrin, cholera toxin subunit B, and dextran, to aid in data interpretation. Also given the lack of specificity of pharmacological inhibitors, siRNA‐mediated knockdown or co‐localization analysis with endocytic markers may represent better‐suited techniques to establish cellular internalization mechanisms. For future investigations, clathrin‐, caveolae‐independent, and fast endophilin A2‐dependent endocytosis of liposomes and EVs are mechanisms that should be further explored, as they may possess great potential for therapeutic applications.

Overall, exploration of the uptake mechanism and the subsequent intracellular trafficking remains a key area of research, important for achieving the maximum potential of lipid‐based nanoparticles. Despite the increasing number of studies reporting on the internalization mechanisms of liposomes and EVs, the biological relevance in relation to their entry route remains to be fully established. As this review shows, there is a highly complex interplay between the composition of lipid‐based vesicles and their target cells when it comes to uptake. Therefore, it remains essential that the similarities and differences between liposomes and EVs be also compared in head‐to‐head studies. Furthermore, dictating intracellular fate is an objective imperative to having full control of effective drug delivery. The field should work towards a fundamental understanding of the uptake and intracellular fate of nanoparticles to reach its full potential.

## Conflict of Interest

The authors declare no conflict of interest.

## Supporting information

Supplemental Table 1

Supplemental Table 2

Supplemental Table 3

Supplemental Table 4
