# Supplementary material for: A Comparison of Cellular Uptake Mechanisms, Delivery Efficacy, and Intracellular Fate between Liposomes and Extracellular Vesicles
Source: Adv Healthc Mater. 2023 Jul 9;12(25):2300319. doi: 10.1002/adhm.202300319 (PMC11469107; doi:10.1002/adhm.202300319)
Supplement: Supplementary file 1 — Supplemental Table 1 [file ADHM-12-2300319-s004.pdf]

ADVANCED HEALTHCARE MATERIALS

Supporting Information

for *Adv. Healthcare Mater.*, DOI 10.1002/adhm.202300319

A Comparison of Cellular Uptake Mechanisms, Delivery Efficacy, and Intracellular Fate between Liposomes and Extracellular Vesicles

*Timea B. Gandek, Luke van der Koog and Anika Nagelkerke**

Supplementary Table 1. Overview of lipids and lipid-like components available for the generation of liposomes.

Abbreviation	Name in full
Amide	2-hydroxy- *N*-methyl-*N*,*N*-bis(2-tetradecanamidoethyl)ethanaminium chloride
CHEMS	Cholesteryl hemisuccinate
DC-Cholesterol	3ß-[*N*-(*N'*,*N'*-dimethylaminoethane)-carbamoyl]-Cholesterol
DDA	Dimethyldioctadecylammonium
DDAB	Dimethyldioctadecylammonium bromide
DHPE	1,2-dihexadecanoyl-sn-glycero-3-phosphoethanolamine
DLin-MC3-DMA	dilinoleylmethyl-4-dimethylaminobutyrate
DLPC	1,2-dilauroyl-sn-glycero-3-phosphocholine
DMG	1,2-dimyristoyl-rac-glycerol
DMPC	1,2-dimyristoyl-sn-glycero-3-phosphocholine
DMPG	1,2-dimyristoyl-sn-glycero-3-phosphoglycerol
DOPA	1,2-dioleoyl-sn-glycero-3-phosphatidic acid
DOPC	1,2-dioleoyl-sn-glycero-3-phosphocholine
DOPE	1,2-dioleoyl-sn-glycero-3-phosphorylethanolamine
DOPG	1,2-dioleoyl-sn-glycero-3-phosphoglycerol
DOPS	1,2-dioleoyl-sn-glycero-3-phospho-*L*-serine
DOTAP	1,2-dioleoyl-3-trimethylammonium-propane
DPPC	1,2-dipalmitoyl-sn-glycero-3-phosphocholine
DPPE	1,2-dipalmitoyl-sn-glycero-3-phosphorylethanolamine
DPPG	1,2-dipalmitoyl-sn-glycero-3-phosphoglycerol
DPPS	1,2-dipalmitoyl-sn-glycero-3-phospho-*L*-serine
DSPA	1,2-distearoyl-sn-glycero-3-phosphatidic acid
DSPC	1,2-distearoyl-sn-glycero-3-phosphocholine
DSPE	1,2-distearoyl-sn-glycero-3-phosphorylethanolamine
DSTAP	1,2-distearoyl-3-trimethylammonium-propane
EPC	Egg phosphatidylcholine
HSPC	Hydrogenated Soy Phosphatidylcholine
LC	*N*-Stearoyldihydrolactocerebroside
MMP-9	Matrix metalloproteinase-9
MPB-PE	*N*-(4-(*p*-maleimidophenyl)butyryl)dimyristoyl phosphatidylethanolamine
PC	Phosphatidylcholine
PC-98T	Egg yolk lecithin
PE	Phosphatidylethanolamine
PG	Phosphatidylglycerol
PHC	Palmitoylhomocysteln
PI	Phosphatidylinositol
PS	Phosphatidylserine
SA	Stearylamine
SM	Sphingomyelin
SPC	Soy Phosphatidylcholine
TFPE	1,2-dioleoyl-sn-glycero-3-phosphoethanolamine-*N*-(dipyrrometheneborondifluoride)butanoyl
