# Supplementary material for: A Comparison of Cellular Uptake Mechanisms, Delivery Efficacy, and Intracellular Fate between Liposomes and Extracellular Vesicles
Source: Adv Healthc Mater. 2023 Jul 9;12(25):2300319. doi: 10.1002/adhm.202300319 (PMC11469107; doi:10.1002/adhm.202300319)
Supplement: Supplementary file 2 — Supplemental Table 2 [file ADHM-12-2300319-s001.pdf]

ADVANCED HEALTHCARE MATERIALS

Supporting Information

for *Adv. Healthcare Mater.*, DOI 10.1002/adhm.202300319

A Comparison of Cellular Uptake Mechanisms, Delivery Efficacy, and Intracellular Fate between Liposomes and Extracellular Vesicles

*Timea B. Gandek, Luke van der Koog and Anika Nagelkerke**

Supplementary Table 2. Uptake of liposomes and EVs via clathrin-mediated endocytosis in various recipient cells.

Inhibitors	Targets	Drug delivery systems	Recipient cells	Inhibitor concentrations	Incubation times of drug delivery systems with cells	Serum supplemen- tation	Inhibition efficiencies	Intracellular fate of drug delivery systems	Key results	Ref
Chlorpromazine	Amphipathic pharmacological inhibitor that depletes the plasma membrane of clathrin and adaptor protein AP2 complex by their assembly at the site of endosomal compartments thus hindering internalization via clathrin-mediated endocytosis.	Gold-encapsulated liposomes (lipid composition not mentioned)	UROtsa cells	14-28 μM	1 h	n.r.	~30-95% inhibition	n.r.	• Gold-encapsulated liposomes entered UROtsa cells predominantly in a clathrin-dependent manner.	[1]
		DOPE:DC-Cholesterol lipoplexes	A549 cells	20 μg mL^{-1}	4 h	+	34% inhibition<br>95% silencing	Endosomes	• Clathrin-mediated endocytosis was one of the main internalization mechanisms of DOPE:DC-Cholesterol lipoplexes, along with macropinocytosis.<br>• The transfection efficiency of lipoplexes was almost completely abolished following inhibition of clathrin-dependent endocytosis.	[2]
		DOPE:CHEMS liposomes	COS-7 cells<br>HUVECs	28 μM	15 minutes	n.r.	30% inhibition	Endosomes	• DOPE:CHEMS liposomes were partly internalized through clathrin-mediated endocytosis in both HUVECs and COS-7 cells.	[3]
		DOTAP:DOPC:Cholesterol lipoplexes	A549 cells	10 μg mL^{-1}	4 h	n.r.	~35% inhibition	n.r.	• Cationic lipoplex formulations, DOTAP:DOPC:Cholesterol and Lipofectamine 2000, were partly	[4]

									internalized through clathrin-associated pathways in HUVECs and A549 cells	
		Lipofectamine 2000 lipoplexes					~45% inhibition		● Uptake of the negatively charged DOPC:SM:Cholesterol:DOPS:DOPE EV-mimicking lipoplexes was not clathrin-mediated.	
		DOPC:SM:Cholesterol:DOPS:DOPE EV-mimicking lipoplexes					- (based on transfection)		● DOTAP:DOPC:Cholesterol and Lipofectamine 2000 lipoplexes had a greater transfection efficiency than those of EV-mimicking lipoplexes.	
			HUVECs				~25% inhibition<br>~30% inhibition<br>-<br>(based on transfection)			
		PC-98T:Cholesterol-enveloped plasmid-laden chitosan nanoparticles	Human conjunctival epithelial cells	5 µg mL^{-1}	2 h	n.r.	~63% inhibition<br>~58% inhibition	Endo-lysosomal compartments	● Both plasmid-laden chitosan nanoparticle formulations were predominantly taken up via clathrin-mediated internalization in conjunctival epithelial cells.<br>● The DOTAP based formulation had a slightly higher uptake than plasmid-laden chitosan nanoparticles and more than two-	[5]

		PC-98T:Cholesterol:DOTAP-enveloped plasmid-laden chitosan nanoparticles							fold increase in internalization than those lacking DOTAP.<br>● DOTAP insertion facilitated lysosomal escape, which in turn greatly enhanced their transfection ability both in vitro and in vivo.	
		DOTAP:DOPE lipoplexes<br><br>DOPE:Cholesterol lipoplexes	COS-7 cells	8 μg mL^{-1}	1 h	-	~60% uptake inhibition<br>~95% silencing<br><br>~60% uptake and silencing	n.r.<br><br>Early endosomes and lysosomes	● Both DOTAP:DOPE and DOPE:Cholesterol lipoplexes were internalized predominantly via clathrin-mediated endocytosis in COS-7 cells.	[6]
		DOPC:Cholesterol liposomes<br><br>DOPG:Cholesterol liposomes	HeLa cells	10 μg mL-1	5 h	+	-<br>55% inhibition	Lysosomes	● Clathrin-mediated endocytosis was one of the two prevailing internalization mechanisms for DOPG:Cholesterol liposomes in HeLa and TRP3 cells, but not in A549 cells. Meanwhile, clathrin-mediated endocytosis had no contribution in the internalization of DOPC:Cholesterol liposomes in any of the recipient cell types tested.<br>● The effect of the anionic surface charge of DOPG:Cholesterol liposomes and the resulting protein corona can be attributed to the emergence of the	[7]
			A549 cells				~100% increase<br>~85% increase	n.r.		
			TRP3 cells				-<br>~65% inhibition	n.r.		

									additional clathrin-mediated endocytosis mechanism in cells.	
		Amide:DOPE lipoplexes<br><br>Amide:Cholesterol lipoplexes	SK-HEP1 cells	2.5 µg mL^{-1}	48 h	n.r.	~40% inhibition<br><br>20% inhibition<br><br>(based on transfectio n)	n.r.	● Clathrin-mediated internalization was one of the two prevailing uptake mechanisms in the uptake of Amide:DOPE lipoplexes in SK-HEP1 cells. Meanwhile, clathrin-associated pathways had a minor role in the uptake of Amide:Cholesterol lipoplexes.<br>● Amide:DOPE lipoplexes were superior to those of Amide:Cholesterol in terms of transfection efficiency.	[8]
		PE:PC:PI:PS liposomes	Huh7.5 cells	25 µM	1 h	-	13% inhibition	Endoplasmic reticulum	● A minor fraction of liposomes may have been internalized through clathrin-mediated endocytosis in Huh7.5 cells.<br>● Uptake was mediated by scavenger and low density lipoprotein receptors.<br>● The lipid composition of PE:PC:PI:PS liposomes actively targeted and fused with endoplasmic reticulum.	[9]
		Charge-reversal amphiphile lipoplexes	CHO-K1 cells	10 µM	3 h	-	~30-35% uptake inhibition<br><br>and silencing	n.r.	● CHO-K1 cells internalized charge-reversal amphiphile lipoplexes containing GFP or β-galactosidase-encoding DNA partially through clathrin-dependent endocytosis.	[10]

									• Lipoplexes did not accumulate inside lysosomes.	
		DOTAP:DOPC liposomes<br><br>DOTAP:Cholesterol liposomes	HeLa cells	50 μM	2 h	n.r.	~65% inhibition	Lysosomes, mitochondria, endoplasmic reticulum, trans-Golgi complex	• Both DOTAP:DOPC and DOTAP:Cholesterol liposomes were internalized through clathrin-dependent endocytosis in HeLa cells.<br>• Both lipid-based nanocarriers were transported to lysosomes, followed by their accumulation in mitochondria, endoplasmic reticulum, and trans-Golgi complex.	[11]
		DPPC liposomes<br><br>Hybrid DPPC:EVs-derived from Sk-hep1 cells	Sk-hep1 cells	30 μM	4 h	n.r.	-<br><br>~25% inhibition	Lysosomes and endoplasmic reticulum<br><br>Trans-Golgi complex and endoplasmic reticulum	• Clathrin-mediated endocytosis was partly responsible for the internalization of DPPC:EVs in parental cell lines, whereas it did not play a role in the internalization of DPPC liposomes.<br>• DPPC:EVs circumvented lysosomal accumulation, in contrast to DPPC liposomes, and accumulated mainly in the endoplasmic reticulum and trans-Golgi complex.<br>• DPPC:EVs showed 1.7-fold increased siRNA transfection efficiency than DPPC liposomes.<br>• DPPC:EVs demonstrated enhanced antitumor efficacy in	[12]

									HCC bearing mice, compared to DPPC liposomes.	
		Adipose-derived regenerative cell-derived EVs	Cardiomyocytes	10 μM	n.r.	n.r.	~50% inhibition<br><br>(in both normoxia and hypoxia conditions)	n.r.	● Clathrin-mediated endocytosis of EVs isolated from adipose-derived regenerative cells played a critical role in suppressing damage induced by hypoxia conditions in cardiomyocytes through miR-214 delivery both in vivo and in vitro.<br><br>● Hypoxia conditions stimulated the uptake of EVs via clathrin-dependent endocytosis in cardiomyocytes, compared to normal oxygen conditions.	[13]
		Normal syncytiotrophoblast-derived EVs<br><br>Preeclamptic syncytiotrophoblast-derived EVs	HCAECs	10 μg mL^{-1}	2 h	n.r.	98% inhibition<br><br>95% inhibition	n.r.	● EVs were primarily taken up via clathrin-mediated endocytosis in a dynamin-dependent manner.<br><br>● The cellular internalization of vesicles was dependent on PI3K activity.<br><br>● Uptake of normal EVs down-regulated ICAM-1 protein expression, compared with preeclamptic syncytiotrophoblast-derived EVs that had no effect.	[14]
		MSC-derived EVs<br><br>HSPC:Cholesterol liposomes	MSCs	5 μg mL^{-1}	2 h	n.r.	38% inhibition	n.r.	● HSPC:Cholesterol liposomes and MSC-derived EVs were taken up partly through clathrin-mediated endocytosis.	[15]

			NIH3T3 cells				35% inhibition<br><br>(in MSCs, NIH3T3 reported to have similar values)		• EVs exhibited a two-fold higher uptake than liposomes.	
		PC12 cell-derived EVs	BMSCs	10 μM	3 h	n.r.	41% inhibition	n.r.	• Uptake of PC12 cell-derived EVs proceeded partly through clathrin-mediated endocytosis in BMSCs.<br><br>• EVs delivered microRNAs, i.e. miR-21, through which transforming growth factor β receptor II and tropomyosin-1 expression were downregulated in recipient cells.	[16]
		BMSC-derived EVs	Multiple myeloma 1S cells	5-10 μM<br><br>20 μM	4 h	n.r.	~25-30% inhibition<br><br>-	n.r.	• Clathrin-mediated endocytosis may have contributed to the internalization of BMSC-derived EVs in multiple myeloma 1S cells, but not in others. Furthermore, the highest concentration of chlorpromazine did not inhibit uptake of these nanocarriers.<br><br>• Internalization was dependent on heparin, actin, dynamin, and PI3K activity.<br><br>• EV delivery promoted cell proliferation and facilitated chemotherapeutic resistance to bortezomib in multiple myeloma	[17]

									cell lines, namely MM1S, RPMI 8226, and U266.	
		K562 and MT4 cell-derived EVs	RAW264.7 macrophages	50 μM	2 h	n.r.	24% inhibition	Phago-lysosomes	● A minor fraction of K562 and MT4-derived EVs was internalized in a clathrin-dependent manner. The majority was internalized via phagocytosis in various phagocytes, whilst in non-phagocytic cells they remained attached to the cell membrane.	[18]
Dynasore	Inhibits the GTPase activity of dynamin, thereby halting plasma membrane scission.	DLin-MC3-DMA:DSPC:Cholesterol:DMG-PEG lipoplexes	HeLa cells	80 μM	4 h	+	~75% inhibition	Endo-lysosomal compartments	● DLin-MC3-DMA:DSPC:Cholesterol:DMG-PEG lipoplexes were internalized via clathrin-mediated endocytosis, which further stimulated uptake via macropinocytosis.<br>● Only 1-2% of siRNAs were able to escape degradation, during lipoplex translocation from early to late endosomes.	[19]
		Adipose-derived regenerative cell-derived EVs	Cardiomyocytes	50-100 μM	n.r.	n.r.	~50% inhibition<br>(in hypoxia conditions)<br>-	n.r.	● Clathrin-mediated endocytosis of EVs played a critical role in suppressing damage induced by hypoxia conditions in cardiomyocytes through miR-214 delivery both in vivo and in vitro.<br>● Hypoxia conditions stimulated the uptake of EVs via clathrin-dependent endocytosis in	[13]

							(in normoxia conditions)		cardiomyocytes, compared to normal conditions.	
		Normal syncytiotrophoblast-derived EVs<br><br>Preeclamptic syncytiotrophoblast-derived EVs	HCAECs	80 μM	2 h	n.r.	86% inhibition<br><br>76% inhibition	n.r.	• EVs were primarily taken up via clathrin-mediated endocytosis in a dynamin-dependent manner.<br>• The cellular internalization of vesicles was dependent on PI3K activity.<br>• Uptake of normal EVs down-regulated ICAM-1 protein expression, compared with preeclamptic syncytiotrophoblast-derived EVs that had no effect.	[14]
FK506	Interferes with calcineurin, subsequently affecting dynamin.	DOPC:DOPG liposomes	Hippocampal neurons	1 μM	30 minutes	n.r.	¬95% inhibition	50% of lipids recycled back to the plasma membrane, oligo-nucleotides accumulated in nuclei	• Following binding of DOPC:DOPG liposomes with low density lipoprotein receptors, the nanocarriers were taken up in a dynamin and PI3K activity-dependent manner, suggesting clathrin-mediated endocytosis.<br>• After internalization, the encapsulated oligonucleotides were located in nuclei within 1-3 h post-incubation and 50% of liposomal phospholipids were recycled back to the plasma membrane.	[20]
Wortmannin	Inhibits re-arrangement of actin filaments which regulate by the activity of			100 nM			¬80% inhibition			

	phosphatidylinositol 3-kinase.	Normal syncytiotrophoblast-derived EVs<br><br>Preeclamptic syncytiotrophoblast-derived EVs	HCAECs	1 μM	2 h	n.r.	94% inhibition<br><br>89% inhibition	n.r.	● EVs were primarily taken up via clathrin-mediated endocytosis in a dynamin-dependent manner.<br>● The cellular internalization of EVs was dependent on PI3K activity.<br>● Uptake of normal EVs down-regulated ICAM-1 protein expression, compared with preeclamptic syncytiotrophoblast-derived EVs that had no effect.	[14]
Amantadine	Prevents clathrin recycling to the plasma membrane.	DMPC:DMPG liposomes	HCAECs	1 mM	30 minutes	-	46% inhibition without TNF-α<br><br>77% inhibition with TNF-α	n.r.	● HCAECs internalized DMPC:DMPG liposomes via clathrin-associated pathways.<br>● Inflammatory conditions, such as TNF-α stimulation, induced overexpression of clathrin proteins and subsequently enhanced liposome internalization.	[21]
Hyperosmolar sucrose	Prevents interaction between clathrin and adaptor proteins	DOPC:DOPG liposomes	Hippocampal neurons	0.45 M	30 minutes	n.r.	¬98% inhibition	50% of lipids recycled back to the plasma membrane, oligonucleotides accumulated in nuclei	● Following binding of DOPC:DOPG liposomes with low density lipoprotein receptors, the nanocarriers were taken up dependent on dynamin and PI3K activity, suggesting clathrin-mediated endocytosis.<br>● After internalization, the encapsulated oligonucleotides were located in nuclei within 1-3 h post-incubation and 50% of liposomal phospholipids were	[20]

									recycled back to the plasma membrane.	
		0.86 mol% R8-EPC:Cholesterol or -DOPE:CHEMS lipoplexes<br><br>5.2 mol% R8-EPC:Cholesterol or -DOPE:CHEMS lipoplexes	NIH3T3 cells	0.4 M	1 h	-	~80% inhibition<br><br>~35% inhibition	High lysosomal co-localization<br><br>Partial lysosomal co-localization	• Low density (0.86 mol%) R8-lipoplexes internalized mainly via clathrin-mediated endocytosis in NIH3T3 cells, ultimately accumulating inside lysosomes.<br>• High density (5.2 mol%) R8-lipoplexes were taken up partly via clathrin-associated pathways, which led to partial accumulation inside lysosomes. Hence, high density R8-lipoplexes presented higher transfection efficiency.	[22]
		DOTAP:DOPE lipoplexes<br><br>DOPE:Cholesterol lipoplexes	COS-7 cells	800 μM	1 h	-	~35% inhibition<br><br>~50% inhibition<br><br>(based on transfectio n)	-<br><br>Early endosomes and lysosomes	• Both DOTAP:DOPE and DOPE:Cholesterol lipoplexes were internalized predominantly via clathrin-mediated endocytosis in COS-7 cells.	[6]

		DLPC:Cholesterol:Cholesteryl:PEG liposomes	Zebrafish hepatocytes	300 mM	15 minutes	n.r.	15% inhibition	Endo-lysosomal compartments	• Hepatocytes internalized DLPC:Cholesterol:Cholesteryl:PEG liposomes partially in a clathrin-dependent manner, ultimately accumulating inside lysosomes.<br>• Lipopolysaccharide-dsRNA cocktails encapsulated in liposomes were able to stimulate both pro-inflammatory and antiviral responses in cells.	[23]
			Trout macrophages	150 mM	30 minutes		-			

n.r. = not reported

– = no inhibition

References

[1] B. R. Rajaganapathy, M. B. Chancellor, J. Nirmal, L. Dang, P. Tyagi. *PLoS One* **2015**, *10*, e0122766.
[2] A. Alshehri, A. Grabowska, S. Stolnik. *Sci Rep* **2018**, *8*, 3748.
[3] U. S. Huth, R. Schubert, R. Peschka-Suss. *J Control Release* **2006**, *110*, 490-504.
[4] M. Lu, X. Zhao, H. Xing, Z. Xun, S. Zhu, L. Lang, T. Yang, C. Cai, D. Wang, P. Ding. *Int J Pharm* **2018**, *550*, 100-113.
[5] M. Jiang, L. Gan, C. Zhu, Y. Dong, J. Liu, Y. Gan. *Biomaterials* **2012**, *33*, 7621-7630.
[6] Y. U. Bae, B. K. Kim, J. W. Park, Y. B. Seu, K. O. Doh. *Mol Pharm* **2012**, *9*, 3579-3585.
[7] D. Montizaan, K. Yang, C. Reker-Smit, A. Salvati. *Nanomedicine* **2020**, *30*, 102300.
[8] S. C. Maddila, C. Voshavar, P. Arjunan, R. P. Chowath, H. K. R. Rachamalla, B. Balakrishnan, P. Balasubramanian, R. Banerjee, S. Marepally. *Molecules* **2021**, *26*.
[9] S. Pollock, R. Antrobus, L. Newton, B. Kampa, J. Rossa, S. Latham, N. B. Nichita, R. A. Dwek, N. Zitzmann. *FASEB J* **2010**, *24*, 1866-1878.
[10] X. X. Zhang, P. G. Allen, M. Grinstaff. *Mol Pharm* **2011**, *8*, 758-766.
[11] K. Un, K. Sakai-Kato, Y. Goda. *Die Pharmazie - An International Journal of Pharmaceutical Sciences* **2014**, *69*, 525-531.
[12] X. Zhou, Y. Miao, Y. Wang, S. He, L. Guo, J. Mao, M. Chen, Y. Yang, X. Zhang, Y. Gan. *J Extracell Vesicles* **2022**, *11*, e12198.
[13] S. Eguchi, M. Takefuji, T. Sakaguchi, S. Ishihama, Y. Mori, T. Tsuda, T. Takikawa, T. Yoshida, K. Ohashi, Y. Shimizu, R. Hayashida, K. Kondo, Y. K. Bando, N. Ouchi, T. Murohara. *J Biol Chem* **2019**, *294*, 11665-11674.
[14] T. Cronqvist, L. Erlandsson, D. Tannetta, S. R. Hansson. *Placenta* **2020**, *100*, 133-141.
[15] S. Le Saux, H. Aarrass, J. Lai-Kee-Him, P. Bron, J. Armengaud, G. Miotello, J. Bertrand-Michel, E. Dubois, S. George, O. Faklaris, J. M. Devoisselle, P. Legrand, J. Chopineau, M. Morille. *Biomaterials* **2020**, *231*, 119675.
[16] T. Tian, Y. L. Zhu, Y. Y. Zhou, G. F. Liang, Y. Y. Wang, F. H. Hu, Z. D. Xiao. *J Biol Chem* **2014**, *289*, 22258-22267.
[17] C. Tu, Z. Du, H. Zhang, Y. Feng, Y. Qi, Y. Zheng, J. Liu, J. Wang. *Theranostics* **2021**, *11*, 2364-2380.
[18] D. Feng, W. L. Zhao, Y. Y. Ye, X. C. Bai, R. Q. Liu, L. F. Chang, Q. Zhou, S. F. Sui. *Traffic* **2010**, *11*, 675-687.
[19] J. Gilleron, W. Querbes, A. Zeigerer, A. Borodovsky, G. Marsico, U. Schubert, K. Manygoats, S. Seifert, C. Andree, M. Stoter, H. Epstein-Barash, L. Zhang, V. Koteliansky, K. Fitzgerald, E. Fava, M. Bickle, Y. Kalaidzidis, A. Akinc, M. Maier, M. Zerial. *Nat Biotechnol* **2013**, *31*, 638-646.
[20] A. Lakkaraju, Y. E. Rahman, J. M. Dubinsky. *J Biol Chem* **2002**, *277*, 15085-15092.
[21] S. Zhaorigetu, C. Rodriguez-Aguayo, A. K. Sood, G. Lopez-Berestein, B. L. Walton. *J Liposome Res* **2014**, *24*, 182-190.
[22] I. A. Khalil, K. Kogure, S. Futaki, H. Harashima. *J Biol Chem* **2006**, *281*, 3544-3551.
[23] A. Ruyra, M. Cano-Sarabia, S. A. Mackenzie, D. Maspoch, N. Roher. *PLoS One* **2013**, *8*, e76338.
