# Supplementary material for: A Comparison of Cellular Uptake Mechanisms, Delivery Efficacy, and Intracellular Fate between Liposomes and Extracellular Vesicles
Source: Adv Healthc Mater. 2023 Jul 9;12(25):2300319. doi: 10.1002/adhm.202300319 (PMC11469107; doi:10.1002/adhm.202300319)
Supplement: Supplementary file 3 — Supplemental Table 3 [file ADHM-12-2300319-s003.pdf]

ADVANCED
HEALTHCARE
MATERIALS

Supporting Information

for *Adv. Healthcare Mater.*, DOI 10.1002/adhm.202300319

A Comparison of Cellular Uptake Mechanisms, Delivery Efficacy, and Intracellular Fate between Liposomes and Extracellular Vesicles

*Timea B. Gandek, Luke van der Koog and Anika Nagelkerke**

Supplementary Table 3. Uptake of liposomes and EVs via caveolae-mediated endocytosis in various recipient cells.

Inhibitors	Targets	Drug delivery systems	Recipient cells	Inhibitor concentrations	Incubation times of drug delivery systems with cells	Serum supplemen- tation	Inhibition efficiencies	Intracellular fate of drug delivery systems	Key results	Ref
Genistein	Locally disrupts the actin network, resulting in dynamin depletion of the plasma membrane.	Tosyl-17AAG-PC:DSPE-PEG$_{2000}$ liposomes	HeLa cells	n.r.	n.r.	n.r.	Partial inhibition<br><br>(based on microscopy images)	Endoplasmic reticulum and partially in mitochondria	• Tosyl-conjugated 17AAG-PC:DSPE-PEG$_{2000}$ liposomes were taken up via caveolae-mediated endocytosis in HeLa cells and confined to endoplasmic reticulum at later time points.<br><br>• Liposomes induced endoplasmic reticulum stress and DNA impairment, which ultimately led to cell apoptosis.	[1]
		DOPE:Cholesterol lipoplexes	COS-7 cells	150 µM	1 h	+	~15% inhibition<br><br>(based on transfection)	Early endosomes and lysosomes	• After genistein treatment of COS-7 cells, the transfection efficiency of DOPE:Cholesterol lipoplexes in the presence of serum, decreased by 15%, whereas in the absence of serum the inhibitor had no effect. This suggests that caveolae-mediated endocytosis has a minor role in transfection by lipoplexes.	[2]
		Trichomonas vaginalis-derived EVs	BPH-1 cells	200 µM	30 minutes	n.r.	~90% inhibition	n.r.	• Caveolin-1 was deemed crucial for the internalization of *Trichomonas vaginalis*-derived EVs, as HEK293 cells, which naturally exhibit a low caveolae content, elicited ~80% lower uptake compared to BPH-1 and CHO-K1 cells.<br><br>• The uptake of EVs was initiated by binding of 4-α-glucanotransferase	[3]

									protein located on the surface of EVs to heparan sulfates present on the host cell surface.	
		A431 cell-derived EVs	HeLa cells	150-200 μM	4 h	n.r.	¬50% inhibition	n.r.	• The uptake of A431 cell-derived EVs in HeLa cells was dependent on caveolin.	[4]
		BMSC-derived EVs	Multiple myeloma 1S cells	50 μM	4 h	n.r.	~30% inhibition	n.r.	• Multiple myeloma 1S cells internalized BMSC-derived EVs partly in a caveolae-dependent manner.<br>• Internalization was dependent on heparin, actin, dynamin, and PI3K activity.<br>• EV delivery promoted cell proliferation and facilitated chemotherapeutic resistance to bortezomib in multiple myeloma cell lines, namely MM1S, RPMI 8226, and U266.	[5]
		MSC-derived EVs<br><br>HSPC:Cholesterol liposomes	MSCs<br><br>NIH3T3 cells	200 μM	2 h	n.r.	73% inhibition<br><br>92% inhibition<br><br>(in MSCs, NIH3T3 had similar values)	n.r.	• HSPC:Cholesterol liposomes and MSC-derived EVs were taken up mainly through caveolae-mediated endocytosis.<br>• Internalization of EVs was predominantly mediated in a cholesterol-dependent manner, whereas liposome uptake was independent of cholesterol.	[6]

									• Liposome uptake was enhanced after cholesterol sequestration in both cell types.<br>• EVs exhibited a two-fold higher uptake than liposomes.	
Dynasore	Inhibits the GTPase activity of dynamin, consequently halting plasma membrane scission.	Epstein-Barr virus-infected B cell-derived EVs	CNE1	150 μM	2 h	n.r.	~85-90% inhibition	Early and late endosomes	• Uptake of EVs isolated from Epstein-Barr virus-infected B cells was mediated by caveolae-mediated endocytosis in several epithelial cell lines, such as CNE1, HONE1, NU-GC-3 and A549.<br>• Following internalization of EVs derived from type III latency virus infected cells, latent membrane protein 1 was transferred to recipient cells, consequently increasing cell proliferation rate and ICAM-1 expression.	[7]
		BMSC-derived EVs	Multiple myeloma 1S cells	50 μM	4 h	n.r.	~45% inhibition	n.r.	• Multiple myeloma 1S cells internalized BMSC-derived EVs partly in a caveolae-dependent manner.<br>• Internalization was dependent on heparin, actin, dynamin, and PI3K activity.<br>• EV delivery promoted cell proliferation and facilitated chemotherapeutic resistance to bortezomib in multiple myeloma cell lines, namely MM1S, RPMI 8226, and U266.	[5]

Filipin	Interacts with 3-h-hydroxysterols followed by the formation of filipin-sterol complexes. This formation inhibits caveolae-mediated endocytosis by disassembling the filamentous caveolin-1 coat.	PE:PC:PI:PS liposomes	Huh7.5 cells	5 µg mL^{-1}	1 h	-	73% inhibition	Endoplasmic reticulum	• Internalization of PE:PC:PI:PS liposomes occurred mainly through caveolae-associated pathways in a microtubule-dependent manner in Huh7.5 cells.<br>• The lipid composition of PE:PC:PI:PS liposomes was able to actively target and fuse with the endoplasmic reticulum.	[8]
		Amide:DOPE lipoplexes<br>Amide:Cholesterol lipoplexes	SK-HEP1 cells	7.5 µg mL^{-1}	48 h	n.r.	50% increase<br>-<br>(based on transfection)	n.r.	• Caveolae-mediated inhibition significantly increased the transfection efficiency of Amide:DOPE lipoplexes in SK-HEP1 cells.<br>• Caveolae-associated pathways did not contribute to the internalization or transfection of Amide:Cholesterol lipoplexes.<br>• Amide:DOPE lipoplexes were superior to those of Amide:Cholesterol in terms of transfection efficiency.	[9]
		DOPE:CHEMS liposomes	COS-7 cells<br>HUVECs	5-9 µg mL^{-1}	1 h	n.r.	~65-80% inhibition<br>~55-85% inhibition	Endosomes	• DOPE:CHEMS liposomes were predominantly internalized through caveolae-mediated endocytosis in both HUVECs and COS-7 cells.	[10]
		DPPC liposomes<br>Hybrid DPPC:EVs-derived from Sk-hep1 cells	Sk-hep1 cells	5 µg mL-1	4 h	n.r.	~50% inhibition	Lysosomes and endoplasmic reticulum	• Caveolae-mediated endocytosis was solely responsible for the internalization of DPPC liposomes,	[11]

							~30% inhibition	Trans-Golgi complex and endoplasmic reticulum	whereas it was only partly responsible for the internalization of DPPC:EVs.<br>• DPPC:EVs circumvented lysosomal accumulation, in contrast to DPPC liposomes, and accumulated mainly in the endoplasmic reticulum and trans-Golgi complex.<br>• DPPC:EVs showed 1.7-fold increased siRNA transfection efficiency than DPPC liposomes.<br>• DPPC:EVs demonstrated enhanced antitumor efficacy in HCC bearing mice, compared to DPPC liposomes.	
		Trichomonas vaginalis-derived EVs	BPH-1 cells	5 µg mL^{-1}	30 minutes	n.r.	~82% inhibition	n.r.	• Caveolin-1 was deemed crucial for the internalization of *Trichomonas vaginalis*-derived EVs, as HEK293 cells, which naturally exhibit a low caveolae content, elicited ~80% lower uptake compared to BPH-1 and CHO-K1 cells.<br>• The uptake of EVs was initiated by binding of the 4-α-glucanotransferase protein located on the surface of EVs to heparan sulfates present on the host cell surface.	[3]
		Polyampholyte-DOPC:DOPE liposomes	L929 cells	2.5-5 µg mL^{-1}	8 h	n.r.	~20-40% inhibition	Endo-lysosomal compartments	• Polyampholytes-DOPC:DOPE liposomes were taken up partly by caveolae-mediated endocytosis, for non-functionalized this was the only internalization mechanism.<br>• Functionalization of liposomes with polyampholytes enabled endosomal	[12]
		DOPC:DOPE liposomes		5 µg mL^{-1}			~30% inhibition			

									escape and cytosolic release of lysozymes.	
		C-TAT-Cholesterol:SPC:DSPE-PEG liposomes	B16F1 cells	5 µg mL^{-1}	4 h	n.r.	~29% inhibition	n.r.	• C-TAT-Cholesterol:SPC:DSPE-PEG liposomes were partially taken up through caveolae-mediated endocytosis in B16F1 cells.<br>• In the presence of the exogenous reducing agent glutathione, PEG was cleaved from the surface of liposomes, therefore exposing TAT and ultimately improving cellular uptake.<br>• Loading of liposomes with paclitaxel, presented a tumor inhibition of ~70% in B16F1 tumor-bearing mice.	[13]
		PC-98T:Cholesterol-enveloped plasmid-laden chitosan nanoparticles<br><br>PC-98T:Cholesterol:DOTAP-enveloped plasmid-laden chitosan nanoparticles	Human conjunctival epithelial cells	5 µg mL^{-1}	2 h	n.r.	~32% inhibition<br><br>~36% inhibition	Endo-lysosomal compartments	• Both plasmid-laden chitosan nanoparticle formulations were partly taken up via caveolae-mediated internalization in conjunctival epithelial cells.<br>• The DOTAP based formulation had a slightly higher uptake than plasmid-laden chitosan nanoparticles and more than two-fold increase in internalization than those lacking DOTAP.<br>• DOTAP insertion facilitated lysosomal escape, which in turn greatly enhanced their transfection ability both in vitro and in vivo.	[14]

		DOTAP:DOPE lipoplexes	COS-7 cells	1 µg mL^{-1}	1 h	-	~20% inhibition<br><br>(based on transfection)	n.r.	• After filipin treatment of COS-7 cells, the transfection efficiency of DOTAP:DOPE lipoplexes in the absence of serum, decreased by ~20%, whereas the uptake mechanism remained unaffected. This suggests that caveolae-mediated endocytosis has a minor role in transfection by lipoplexes.	[2]
Methyl-β-cyclodextrin	Depletes the plasma membrane of cholesterol.	Gold-encapsulated liposomes	UROtsa cells	6-9 mM	1 h	n.r.	~45-85% inhibition	n.r.	• Gold-encapsulated liposomes entered UROtsa cells partially in a caveolae-dependent manner.	[15]
		DOPE:CHEMS liposomes	COS-7 cells<br><br>HUVECs	9 mM<br><br>6 mM	1 h	n.r.	~30% inhibition<br><br>~50% inhibition	Endosomes	• DOPE:CHEMS liposomes were predominantly internalized through caveolae-mediated endocytosis in both HUVECs and COS-7 cells.	[10]
		DOPC:DOPG:MPB-PE liposomes	HeLa cells	15 mM	1 h	n.r.	~45% inhibition	Early endosomes, lysosomes, and trans-Golgi complex	• DOPC:DOPG:MPB-PE liposomes were taken up by HeLa cells mainly through caveolae-dependent pathways and subsequently transported to endo-lysosomal compartments.<br><br>• Liposomal delivery of doxorubicin improved therapeutic activity and led to tumor growth inhibition and systemic toxicity reduction.	[16]

		DOTAP:DOPE lipoplexes	COS-7 cells	9 mM	1 h	-	~15% inhibition<br><br>(based on transfection)	n.r.	• DOTAP:DOPE lipoplexes were partially taken up in a cholesterol-dependent manner in the absence of serum, suggesting the involvement of caveolae-mediated endocytosis in the context of transfection.<br>• The effect of methyl-β-cyclodextrin was greatly influenced by the addition of serum, causing an 80% decrease in transfection efficiency, compared to serum-free medium.	[2]
		DLPC:Cholesterol:Cholesteryl:PEG liposomes	Zebrafish hepatocytes<br><br>Trout macrophages	5 mM	15 minutes<br><br>30 minutes	n.r.	60% inhibition<br><br>31% inhibition	Endo-lysosomal compartments	• Hepatocytes internalized DLPC:Cholesterol:Cholesteryl:PEG liposomes mainly through caveolae-mediated endocytosis, ultimately accumulating inside lysosomes.<br>• Lipopolysaccharide-dsRNA cocktails encapsulated in liposomes were able to stimulate both pro-inflammatory and antiviral responses in cells.	[17]
		Trichomonas vaginalis-derived EVs	BPH-1 cells	25 mM	30 min	n.r.	~90% inhibition	n.r.	• Caveolin-1 was deemed crucial for the internalization of *Trichomonas vaginalis*-derived EVs, since HEK293 cells, which naturally exhibit a low caveolae content, elicited ~80% lower uptake compared to BPH-1 and CHO-K1 cells.<br>• The uptake of EVs is initiated by binding of 4-α-glucanotransferase protein located on the surface of EVs to heparan sulfates present on the host cell surface.	[3]

		Normal syncytiotrophoblast-derived EVs<br><br>Preeclamptic syncytiotrophoblast-derived EVs	HCAECs	5 mM	2 h	n.r.	60% inhibition<br><br>45% inhibition	n.r.	• Given that cholesterol depletion reduced nanoparticle uptake by 45-60%, caveolae-mediated endocytosis was the secondary entry route for normal and preeclamptic syncytiotrophoblast-derived EVs.<br>• Uptake of normal syncytiotrophoblast-derived EVs down-regulated ICAM-1 protein expression, compared with preeclamptic syncytiotrophoblast-derived EVs that had no effect.	[18]
		MSC-derived EVs<br><br>HSPC:Cholesterol liposomes	MSCs<br><br>NIH3T3 cells	3 mg mL^{-1}	2 h	n.r.	75% inhibition<br><br>86% increase<br><br>(in MSCs, NIH3T3 had similar values)	n.r.	• HSPC:Cholesterol liposomes and MSC-derived EVs were taken up mainly through caveolae-mediated endocytosis.<br>• Internalization of EVs was predominantly mediated in a cholesterol-dependent manner, whereas liposome uptake was independent of cholesterol.<br>• Liposome uptake was enhanced after cholesterol sequestration in both cell types.<br>• EVs exhibited a two-fold higher uptake than liposomes.	[6]
Nystatin	Sequestrates cholesterol from the plasma membrane.	DOPC:DOPG:MPB-PE liposomes	HeLa cells	50 µg mL^{-1}	1 h	n.r.	~40% inhibition	Endosomes, lysosomes, and trans-Golgi complex	• DOPC:DOPG:MPB-PE liposomes were taken up by HeLa cells mainly through caveolae-dependent pathways and subsequently transported to endo-lysosomal compartments.	[16]

									• Liposomal delivery of doxorubicin improved therapeutic activity and led to tumor growth inhibition and systemic toxicity reduction.	
		Amide:DOPE lipoplexes<br><br>Amide:Cholesterol lipoplexes	SK-HEP1 cells	20 μg mL^{-1}	48 h	n.r.	Three to four-fold increase<br><br>-<br><br>(based on transfection)	n.r.	• Caveolae-mediated inhibition significantly increased the transfection efficiency of Amide:DOPE lipoplexes in SK-HEP1 cells.<br><br>• Caveolae-associated pathways did not contribute to the internalization or transfection of Amide:Cholesterol lipoplexes.<br><br>• Amide:DOPE lipoplexes were superior to those of Amide:Cholesterol in terms of transfection efficiency.	[9]
Nocodazole	Disrupts microtubules.	PE:PC:PI:PS liposomes	Huh7.5 cells	25 μM	1 h	-	63% inhibition	Endoplasmic reticulum	• Internalization of PE:PC:PI:PS liposomes occurred mainly through caveolae-associated pathways in a microtubule-dependent manner in Huh7.5 cells.<br><br>• The lipid composition of PE:PC:PI:PS liposomes were able to actively target and fuse with endoplasmic reticulum.	[8]
Indomethacin	Blocks the internalization of caveolae.			100 μM			49% inhibition			
		DOTAP:DOPC:Cholesterol lipoplexes	A549 cells	8 μg mL^{-1}	4 h	n.r.	~35% inhibition	n.r.	• DOTAP:DOPC:Cholesterol and Lipofectamine 2000 lipoplexes were internalized through clathrin-associated pathways, whereas the negatively charged DOPC:SM:Cholesterol:DOPS:DOP	[19]

		Lipofectamine 2000 lipoplexes<br><br>DOPC:SM:Cholesterol:DOPS:DOPE EV-mimicking lipoplexes					~35% inhibition<br><br>~30% inhibition<br><br>(based on transfection)		E EV-mimicking lipoplexes preferred caveolae-mediated endocytosis in A549 cells.<br>● The internalization of EV-mimicking lipoplexes in HUVECs emerged only through caveolae-mediated endocytosis, whereas in COS-7 cells there was an additional mechanism present. Thus caveolae-mediated internalization of these lipoplexes is cell type-dependent.<br>● EV-mimicking lipoplexes presented a three-fold silencing efficiency compared to those of PC:Cholesterol.<br>● DOTAP:DOPC:Cholesterol and Lipofectamine 2000 lipoplexes had a greater transfection efficiency than those of EV-mimicking lipoplexes.	
			HUVECs				~25% inhibition<br><br>~20% inhibition<br><br>~50% inhibition<br><br>(based on transfection)			

n.r. = not reported

- = no inhibition

References

[1] C. Ghosh, A. Nandi, S. Basu. *ACS Appl Bio Mater* **2019**, *2*, 3992-4001.
[2] Y. U. Bae, B. K. Kim, J. W. Park, Y. B. Seu, K. O. Doh. *Mol Pharm* **2012**, *9*, 3579-3585.
[3] A. K. Rai, P. J. Johnson. *Proc Natl Acad Sci U S A* **2019**, *116*, 21354-21360.
[4] H. Costa Verdera, J. J. Gitz-Francois, R. M. Schiffelers, P. Vader. *J Control Release* **2017**, *266*, 100-108.
[5] C. Tu, Z. Du, H. Zhang, Y. Feng, Y. Qi, Y. Zheng, J. Liu, J. Wang. *Theranostics* **2021**, *11*, 2364-2380.
[6] S. Le Saux, H. Aarrass, J. Lai-Kee-Him, P. Bron, J. Armengaud, G. Miotello, J. Bertrand-Michel, E. Dubois, S. George, O. Faklaris, J. M. Devoisselle, P. Legrand, J. Chopineau, M. Morille. *Biomaterials* **2020**, *231*, 119675.
[7] A. Nanbo, E. Kawanishi, R. Yoshida, H. Yoshiyama. *J Virol* **2013**, *87*, 10334-10347.
[8] S. Pollock, R. Antrobus, L. Newton, B. Kampa, J. Rossa, S. Latham, N. B. Nichita, R. A. Dwek, N. Zitzmann. *FASEB J* **2010**, *24*, 1866-1878.
[9] S. C. Maddila, C. Voshavar, P. Arjunan, R. P. Chowath, H. K. R. Rachamalla, B. Balakrishnan, P. Balasubramanian, R. Banerjee, S. Marepally. *Molecules* **2021**, *26*.
[10] U. S. Huth, R. Schubert, R. Peschka-Suss. *J Control Release* **2006**, *110*, 490-504.
[11] X. Zhou, Y. Miao, Y. Wang, S. He, L. Guo, J. Mao, M. Chen, Y. Yang, X. Zhang, Y. Gan. *J Extracell Vesicles* **2022**, *11*, e12198.
[12] S. Ahmed, S. Fujita, K. Matsumura. *Nanoscale* **2016**, *8*, 15888-15901.
[13] H. Fu, K. Shi, G. Hu, Y. Yang, Q. Kuang, L. Lu, L. Zhang, W. Chen, M. Dong, Y. Chen, Q. He. *J Pharm Sci* **2015**, *104*, 1160-1173.
[14] M. Jiang, L. Gan, C. Zhu, Y. Dong, J. Liu, Y. Gan. *Biomaterials* **2012**, *33*, 7621-7630.
[15] B. R. Rajaganapathy, M. B. Chancellor, J. Nirmal, L. Dang, P. Tyagi. *PLoS One* **2015**, *10*, e0122766.
[16] K. I. Joo, L. Xiao, S. Liu, Y. Liu, C. L. Lee, P. S. Conti, M. K. Wong, Z. Li, P. Wang. *Biomaterials* **2013**, *34*, 3098-3109.
[17] A. Ruyra, M. Cano-Sarabia, S. A. Mackenzie, D. Maspoch, N. Roher. *PLoS One* **2013**, *8*, e76338.
[18] T. Cronqvist, L. Erlandsson, D. Tannetta, S. R. Hansson. *Placenta* **2020**, *100*, 133-141.
[19] M. Lu, X. Zhao, H. Xing, Z. Xun, S. Zhu, L. Lang, T. Yang, C. Cai, D. Wang, P. Ding. *Int J Pharm* **2018**, *550*, 100-113.
