# Supplementary material for: A Comparison of Cellular Uptake Mechanisms, Delivery Efficacy, and Intracellular Fate between Liposomes and Extracellular Vesicles
Source: Adv Healthc Mater. 2023 Jul 9;12(25):2300319. doi: 10.1002/adhm.202300319 (PMC11469107; doi:10.1002/adhm.202300319)
Supplement: Supplementary file 4 — Supplemental Table 4 [file ADHM-12-2300319-s002.pdf]

ADVANCED HEALTHCARE MATERIALS

Supporting Information

for *Adv. Healthcare Mater.*, DOI 10.1002/adhm.202300319

A Comparison of Cellular Uptake Mechanisms, Delivery Efficacy, and Intracellular Fate between Liposomes and Extracellular Vesicles

*Timea B. Gandek, Luke van der Koog and Anika Nagelkerke**

Supplementary Table 4. Uptake of liposomes and EVs via macropinocytosis in various recipient cells.

Inhibitors	Targets	Drug delivery systems	Recipient cells	Inhibitor concentrations	Incubation times of drug delivery systems with cells	Serum supplemen- tation	Inhibition efficiencies	Intracellular fate of drug delivery systems	Key results	Ref
Wortmannin	Inhibits re-arrangement of actin filaments which regulate the activity of phosphatidylinositol 3-kinase.	PE:PC:PI:PS liposomes	Huh7.5 cells	100 nM	1 h	-	23% inhibition	Endoplasmic reticulum	• Internalization of PE:PC:PI:PS liposomes occurred partly in a microtubule-dependent manner in Huh7.5 cells.<br>• The lipid composition of PE:PC:PI:PS liposomes was able to actively target and fuse with the endoplasmic reticulum.	[1]
		DOPE:CHEMS liposomes	COS-7 cells<br>HUVECs	10 nM	1 h	n.r.	-<br>~20% inhibition	Endosomes	• DOPE:CHEMS liposomes were taken up partly via macropinocytosis in HUVECs, but not in COS-7 cells. Thus macropinocytotic uptake of these liposomes is cell type-dependent.	[2]
		DOTAP:DOPC lipoplexes<br>DOPE:DC-Cholesterol lipoplexes	CHO-K1 cells	10 μM	4 h	n.r.	~90% inhibition<br>~10% inhibition (based on transfection)	High lysosomal co-localization<br>Partial lysosomal	• Successful transfection of CHO-K1 cells by DOTAP:DOPC lipoplexes was partly dependent on cholesterol.<br>• The transfection potency of DOPE:DC-Cholesterol lipoplexes was predominantly dependent on the presence of cholesterol.<br>• Wortmannin and methyl-β-cyclodextrin were the only inhibitors	[3]

								co-localization	that affected the uptake of lipoplexes, whereas genistein had no effect. Therefore macropinocytosis is a cholesterol sensitive mechanism and important for lipoplex internalization in CHO-K1 cells.<br>● DOPE:DC-Cholesterol formulations were better suited to circumvent lysosomal degradation, compared to their DOTAP:DOPC counterparts.	
		Ca^{2+}-rich DOPG:DOPE lipoplexes<br>Ca^{2+}-poor DOPG:DOPE lipoplexes	MDA-MB-231 cells	100 nM	4 h	+	61% inhibition<br>58% inhibition<br>(based on transfection)	n.r.	● Ca^{2+}-rich lipoplexes entered through additional HSPG-associated pathways, compared to those poor in Ca^{2+}content, which entered solely through macropinocytosis.<br>● Despite their 70% lower siRNA loading efficiency, Ca^{2+}-rich lipoplexes presented approximately seven-fold higher silencing efficiency than their Ca^{2+}-poor counterparts.<br>● Ca^{2+}-rich lipoplexes had almost two-fold higher cellular uptake than those poor in Ca^{2+}.	[4]
		DLPC:Cholesterol:Cholesteryl:PEG liposomes	Zebrafish hepatocytes<br>Trout macrophages	100 nM	15 minutes	n.r.	19% inhibition<br>-	Endo-lysosomal compartments<br>(in hepatocytes)	● Hepatocytes internalized DLPC:Cholesterol:Cholesteryl:PEG liposomes mainly through caveolae-mediated endocytosis and partially in a clathrin-dependent manner, ultimately accumulating inside lysosomes.<br>● Macrophages internalized DLPC:Cholesterol:Cholesteryl:PEG liposomes mainly through caveolae-mediated endocytosis and partially	[5]

									via macropinocytosis, evading lysosomal accumulation.<br>• Lipopolysaccharide-dsRNA cocktails encapsulated in liposomes were able to stimulate both pro-inflammatory and antiviral responses in cells.	
		Charge-reversal amphiphile lipoplexes	CHO-K1 cells	10 μM	3 h	-	78% uptake inhibition<br>95% silencing	n.r.	• CHO-K1 cells internalized charge-reversal amphiphile lipoplexes containing GFP or β-galactosidase-encoding DNA predominantly via macropinocytosis.<br>• The major contributor to the transfection efficiency of lipoplexes was macropinocytosis, owing to the leakiness of macropinosomes.<br>• Although the uptake of lipoplexes was cholesterol-dependent, the mechanism was independent of caveolae-associated pathways suggesting cholesterol-sensitivity of macropinocytosis.<br>• Lipoplexes did not accumulate inside lysosomes.	[6]
		Porphyromonas gingivalis-derived EVs	HeLa cells<br>IHGE cells	100 nM	15 minutes +120 minutes	n.r.	~90% inhibition	Endo-lysosomal compartme nts	• *Porphyromonas gingivalis*-derived EVs entered HeLa and IHGE cells mainly through lipid raft-mediated endocytosis.<br>• EV uptake is dependent on actin polymerization and Rac1, which would indicate macropinocytosis.	[7]

									• EVs associated with cells through fimbriae, a ligand located on the outer membrane of the nanocarrier.<br>• After internalization, EVs were transported to the lysosomes where the nanocarriers survived degradation up to 24 h.	
		A431 cell-derived EVs	HeLa cells	0.2-1 μM	4 h	n.r.	¬30-50% inhibition	n.r.	• The uptake of A431 cell-derived EVs in HeLa cells exhibited a RhoA-, Rac1-, PAK1- and flotillin dependence, therefore macropinocytosis is a major contributor in the internalization of these nanocarriers.	[8]
LY294002	Inhibits re-arrangement of actin filaments which regulate the activity of phosphatidylinositol 3-kinase.	DOPE:CHEMS liposomes	COS-7 cells<br>HUVECs	20-50 μM	1 h	n.r.	-<br>~20% inhibition	n.r.	• DOPE:CHEMS liposomes were taken up partly via macropinocytosis in HUVECs, but not in COS-7 cells. Thus macropinocytotic uptake of these liposomes is cell type-dependent.	[2]
		PC12 cells-derived EVs	BMSCs	10-50 μM	3 h	n.r.	¬25-50 inhibition	n.r.	• Uptake of PC12 cells-derived EVs proceeded partly through macropinocytosis in BMSCs.<br>• EVs delivered microRNAs, i.e. miR-21, through which transforming growth factor β receptor II and tropomyosin-1 expression were downregulated.	[9]

Amiloride or EIPA (an amiloride analogue)	Inhibition of macropinocytosis by lowering intracellular pH thus interfering with Na+/H- exchange which ultimately affects Rac1 activation and actin assembly.	Polyampholytes-DOPC:DOPE liposomes	L929 cells	125-250 µM	8 h	n.r.	~40% inhibition	Endo-lysosomal compartments	• Polyampholytes-DOPC:DOPE liposomes were partly taken up via macropinocytosis, nonfunctionalized liposomes were not.<br>• Functionalization of liposomes with polyampholytes enabled endosomal escape and cytosolic release of lysozymes.	[10]
		DOPC:DOPE liposomes					-			
		DOPE:DC-Cholesterol lipoplexes	A549 cells	0.5 µg mL^{-1}	4 h	+	60% uptake inhibition<br>98% silencing	Endosomes	• DOPE:DC-Cholesterol lipoplexes were taken up by A549 cells via clathrin-mediated process and macropinocytosis in a dynamin-independent, cholesterol-dependent manner.<br>• The internalization efficiency did not reflect directly the transfection efficiency of lipoplexes and endosomal disruption enhanced their potency.	[11]
		DOPC:Cholesterol liposomes	HeLa cells	75 µM	7 h	+	60% inhibition<br>75% inhibition	Lysosomes	• Macropinocytosis was the dominant internalization mechanism for both DOPC:Cholesterol and DOPG:Cholesterol liposomes in HeLa cells, but not in A549 cells.	[12]
		DOPG:Cholesterol liposomes	A549 cells				~100% increase<br>~105% increase	n.r.		

		DLin-MC3-DMA:DSPC:Cholesterol:DMG-PEG lipoplexes	HeLa cells	25-50 μM	4 h	+	~70% inhibition	Endo-lysosomal compartments	• DLin-MC3-DMA:DSPC:Cholesterol:DMG-PEG lipoplexes were internalized via clathrin-mediated uptake, which further stimulates uptake via macropinocytosis.<br>• Macropinocytotic uptake was quantitatively the major entry mechanism.<br>• Only 1-2% of siRNAs were able to escape degradation, during lipoplex translocation from early to late endosomes.	[13]
		DLPC:Cholesterol:Cholesteryl:Cholesterol-PEG liposomes	Zebrafish hepatocytes<br><br>Trout macrophages	50 μM	15 minutes<br><br>30 minutes	n.r.	-<br><br>15% inhibition	Endo-lysosomal compartments<br><br>(in hepatocytes)	• Hepatocytes internalized DLPC:Cholesterol:Cholesteryl:Cholesterol-PEG liposomes mainly through caveolae-mediated endocytosis and partially in a clathrin-dependent manner, ultimately accumulating inside lysosomes.<br>• Macrophages internalized DLPC:Cholesterol:Cholesteryl:PEG liposomes mainly through caveolae-mediated endocytosis and partially via macropinocytosis, evading lysosomal accumulation.<br>• Lipopolysaccharide-dsRNA cocktails encapsulated in liposomes were able to stimulate both pro-inflammatory and antiviral responses in cells.	[5]

		PE:PC:PI:PS liposomes	Huh7.5 cells	250 µM	1 h	-	15% inhibition	Endoplasmic reticulum	• Internalization of PE:PC:PI:PS liposomes occurred partly in a microtubule-dependent manner in Huh7.5 cells.<br>• The lipid composition of PE:PC:PI:PS liposomes were able to actively target and fuse with endoplasmic reticulum.	[1]
		DOTAP:DOPC:Cholesterol lipoplexes	A549 cells	5 µg mL^{-1}	4 h	n.r.	-	n.r.	• Of all the lipid-based vesicles tested, macropinocytotic uptake was present only in the case of DOPC:SM:Cholesterol:DOPS:DOPE EV-mimicking lipoplexes in A549 cells, but not in HUVECs. Macropinocytotic uptake of EV-mimicking lipoplexes is cell type-dependent.<br>• Macropinocytotic uptake was the predominant uptake mechanism in A549 cells for the uptake of EV-mimicking lipoplexes.<br>• EV-mimicking lipoplexes presented a three-fold silencing efficiency compared to those of PC:Cholesterol.<br>• DOTAP:DOPC:Cholesterol and Lipofectamine 2000 lipoplexes had a greater transfection efficiency than those of EV-mimicking lipoplexes.	[14]
		Lipofectamine 2000 lipoplexes					-			
		DOPC:SM:Cholesterol:DOPS:DOPE EV-mimicking lipoplexes					~45% inhibition (based on transfection)			
			HUVECs				-			
							-			
							-			

							(based on transfection)			
		RRRRRRGGRRRRG-DOPE:Cholesterol:DMPG lipoplexes	B16F10 cells	12.5 mM	6 h	+	~80% inhibition	n.r.	● Cell penetrating peptide-conjugated DOPE:Cholesterol:DMPG lipoplexes were internalized via macropinocytosis in B16F10 cells, dependent on HSPG.<br>● After uptake, the knockdown efficiency of lipoplexes against luciferase in luciferase-expressing cells was approximately 70%, similar to that of Lipofectamine 2000, compared to controls.	[15]
		0.86 mol% R8-EPC:Cholesterol or -DOPE:CHEMS lipoplexes<br><br>5.2 mol% R8-EPC:Cholesterol or -DOPE:CHEMS lipoplexes	NIH3T3 cells	5 mM	1 h	-	~40% inhibition<br><br>~80% inhibition	High lysosomal co-localization<br><br>Partial lysosomal co-localization	● Low density (0.86 mol%) R8-lipoplexes internalized partly through macropinocytosis in NIH3T3 cells, ultimately accumulating inside lysosomes.<br>● High density (5.2 mol%) R8-lipoplexes were taken up mainly through macropinocytosis, which led to partial accumulation inside lysosomes. Hence, high density R8-lipoplexes presented higher transfection efficiency.	[16]

		Charge-reversal amphiphile lipoplexes	CHO-K1 cells	20 μM	3 h	-	44% uptake inhibition<br><br>95% silencing	n.r.	• CHO-K1 cells internalized charge-reversal amphiphile lipoplexes containing GFP or β-galactosidase-encoding DNA predominantly via macropinocytosis.<br>• The major contributor to the transfection efficiency of lipoplexes was macropinocytosis, owing to the leakiness of macropinosomes.<br>• Although the uptake of lipoplexes was cholesterol-dependent, the mechanism was independent of caveolae-associated pathways suggesting cholesterol-sensitivity of macropinocytosis.<br>• Lipoplexes did not accumulate inside lysosomes.	[6]
		Amide:DOPE lipoplexes<br><br>Amide:Cholesterol lipoplexes	SK-HEP1 cells	4 μg mL^{-1}	48 h	n.r.	50% inhibition<br><br>-<br><br>(based on transfection)	n.r.	• Macropinocytosis was one of the two prevailing uptake mechanisms in the uptake of Amide:DOPE lipoplexes in SK-HEP1 cells.<br>• Macropinocytosis had no contribution in the uptake of Amide:Cholesterol lipoplexes.<br>• Amide:DOPE lipoplexes were superior to those of Amide:Cholesterol in terms of transfection efficiency.	[17]
		DPPC liposomes	Sk-hep1 cells	5 μg mL-1	4 h	n.r.	~50% inhibition	Lysosomes and endoplasmic reticulum	• Macropinocytosis was partly responsible for the internalization of DPPC:EVs, whereas it did not play a role in the internalization of DPPC liposomes.	[18]

		Hybrid DPPC:EVs-derived from Sk-hep1 cells					~30% inhibition	Trans-Golgi complex and endoplasmic reticulum	• DPPC:EVs circumvented lysosomal accumulation, in contrast to DPPC liposomes, and accumulated mainly in the endoplasmic reticulum and trans-Golgi complex.<br>• DPPC:EVs showed 1.7-fold increased siRNA transfection efficiency than DPPC liposomes.<br>• DPPC:EVs demonstrated enhanced antitumor efficacy in HCC bearing mice, compared to DPPC liposomes.	
		A431 cell-derived EVs	HeLa cells	50-100 µM	4 h	n.r.	¬45-55% inhibition	n.r.	• The uptake of A431 cell-derived EVs in HeLa cells exhibited a RhoA-, Rac1-, PAK1- and flotillin dependence, therefore macropinocytosis is a major contributor in the internalization of these nanocarriers.	[8]
		Stearyl-K4-HeLa cell-derived EVs	E3-EGFR-expressing MDA-MB-231 cells	100 µM	1 h	n.r.	~70% inhibition	n.r.	• Cellular uptake of HeLa cell-derived EVs was greatly enhanced in MDA-MB-231 cells through functionalization with coiled-coil peptides.<br>• The enhanced cellular internalization was achieved through macropinocytosis due to receptor activation.<br>• The therapeutic activity of saporin was enhanced following delivery of functionalized EVs in engineered cells with coiled-coil peptides.	[19]

		PC12 cells-derived EVs	BMSCs	50 μM	3 h	n.r.	¬25% inhibition	n.r.	• Uptake of PC12 cells-derived EVs proceeded partly through macropinocytosis in BMSCs.<br>• EVs delivered microRNAs, i.e. miR-21, through which transforming growth factor β receptor II and tropomyosin-1 expression were downregulated.	[9]
		HeLa cell-derived EVs	A431 cells	20 μM	3h	n.r.	34% inhibition	n.r.	• HeLa cell-derived EVs were taken up via macropinocytosis in A431 cells and in cells expressing oncogenic K-Ras^{G12D} mutation, such as MIA PaCa-2 cells.<br>• Macropinocytotic uptake was enhanced through the stimulation of $CXCR_4$ by co-incubation with stromal cell-derived factor-1α and EGF.<br>• Macropinocytosis stimulation by EGF enhanced the bioactivity of saporin delivered by EVs in A431 cells.	[20]
		R8-HeLa cell-derived EVs<br>R16-HeLa cell-derived EVs	CHO-K1 cells	100 μM	1 h	+	99% inhibition<br>56% inhibition	n.r.	• EV internalization via macropinocytosis in HeLa cells was actively induced by the modification of nanocarriers with arginine-rich cell penetrating peptide sequences.<br>• The anti-cancer activity of ribosome-inactivating protein saporin was greatly enhanced upon surface decoration with R12 and R16 peptide sequences.	[21]

		Stearyl-R8-HeLa cell-derived EVs	HeLa cells	100 µM	1 h	+	25% inhibition	n.r.	• Surface conjugation with stearyl-R8 peptides enhanced the cellular uptake of HeLa cell-derived EVs in recipient cells via macropinocytosis.<br>• The efficiency of cellular internalization was dependent on the density of R8 peptides from the surface of EVs.<br>• The conjugation of liposomes with stearyl-R8 peptides significantly enhanced the cytotoxicity of saporin after delivery via EVs.	[22]
		Fibroblast-like mesenchymal cell-derived EVs	PANC-1 cells	5-75 µM	3 h	-	~35-80% inhibition	n.r.	• Oncogenic K-Ras mutations greatly enhanced the uptake of fibroblast-like mesenchymal cell-derived EVs through macropinocytosis, compared to those of liposomes which remained unaltered.<br>• Biodistribution analysis revealed that the pancreas was among the highest accumulation sites for EVs, next to the liver and lung, indicating a certain affinity.<br>• K-Ras^{G12D} siRNA showed greater silencing capabilities upon EV delivery in contrast with liposomes.<br>• K-Ras^{G12D} siRNA delivery by EVs presented higher pancreatic tumor regression and survival rate in several mice models, compared to liposomes.<br>• Overall EVs presented better cellular uptake and therapeutic utility compared to liposomes, due to	[23]

									internalization via macropinocytosis and membrane protein composition.	
Dynasore	Inhibits the GTPase activity of dynamin, consequently halting plasma membrane scission.	Oli-neu cell-derived EVs	Glial cells	80 µM	2 h	+	~60% inhibition	Endo-lysosomal compartments	• Oligodendroglia-derived EVs were selectively taken up through macropinocytosis, in a dynamin-dependent fashion, by a subpopulation of microglia cells lacking MHC-class-II. Therefore, EV uptake did not provoke an immunological response.<br>• EVs accumulated inside lysosomes both in vitro and in vivo.<br>• EV uptake by microglia cells was probably mediated by the presence of phosphatidylserine within the membrane of the nanocarrier.	[24]
Cytochalasin D	Disrupts actin polymerization.	PC-98T:Cholesterol-enveloped plasmid-laden chitosan nanoparticles<br><br>PC-98T:Cholesterol:DOTAP-enveloped plasmid-laden chitosan nanoparticles	Human conjunctival epithelial cells	10 µg mL^{-1}	2 h	n.r.	38% inhibition<br><br>42% inhibition	Endo-lysosomal compartments	• Both plasmid-laden chitosan nanoparticle formulations were partly taken up via macropinocytosis in conjunctival epithelial cells.<br>• The DOTAP-based formulation had a slightly higher uptake than plasmid-laden chitosan nanoparticles and more than two-fold increase in internalization than those lacking DOTAP.<br>• DOTAP insertion facilitated lysosomal escape, which in turn greatly enhanced their transfection ability both in vitro and in vivo.	[25]
		DOPC:Cholesterol liposomes	HeLa cells	5 µg mL-1	3 h	+	30% inhibition	Lysosomes	• Macropinocytosis was the dominant internalization mechanism for both	[12]

		DOPG:Cholesterol liposomes					80% inhibition		DOPC:Cholesterol and DOPG:Cholesterol liposomes in HeLa and TRP3 cells, but not in A549 cells.	
			A549 cells				~80% increase<br>~150% increase	n.r.		
			TRP3 cells				~20% inhibition<br>~75% inhibition	n.r.		
		C-TAT-Cholesterol:SPC:DSPE-PEG liposomes	B16F1 cells	60 ng mL^{-1}	4 h	n.r.	~36% inhibition	n.r.	• C-TAT-Cholesterol:SPC:DSPE-PEG liposomes were partially taken up through macropinocytosis in B16F1 cells.<br>• In the presence of the exogenous reducing agent glutathione, PEG was cleaved from the surface of liposomes, therefore exposing TAT and ultimately improving cellular uptake.<br>• Loading of liposomes with paclitaxel, presented a tumor inhibition of ~70% in B16F1 tumor-bearing mice.	[26]
		Ca^{2+}-rich DOPG:DOPE lipoplexes	MDA-MB-231 cells	1 μM	4 h	+	-	n.r.	• Ca^{2+}-rich lipoplexes entered through additional HSPG-associated pathways, compared to those poor in Ca^{2+}content, which entered solely through macropinocytosis.<br>• Despite their 70% lower siRNA loading efficiency, Ca^{2+}-rich lipoplexes presented approximately seven-fold higher silencing	[4]
		Ca^{2+}-poor DOPG:DOPE lipoplexes					87% inhibition			

							(based on transfection)		efficiency than their Ca^{2+}-poor counterparts.<br>● Ca^{2+}-rich lipoplexes had almost two-fold higher cellular uptake than those poor in Ca^{2+}.	
		Oli-neu cell-derived EVs	Glial cells	2 μM	2 h	+	~50% inhibition	Endo-lysosomal compartments	● Oligodendroglia-derived EVs were selectively taken up through macropinocytosis, in a dynamin-dependent fashion, by a subpopulation of microglia cells lacking MHC-class-II. Therefore, EV uptake did not provoke an immunological response.<br>● EVs accumulated inside lysosomes both in vitro and in vivo.<br>● EV uptake by microglia cells was probably mediated by the presence of phosphatidylserine within the membrane of the nanocarrier.	[24]
		BMSC-derived EVs	Multiple myeloma 1S cells	2 μM	4 h	n.r.	60% inhibition	n.r.	● The internalization of BMSC-derived EVs required actin remodeling, suggesting an involvement of macropinocytosis.<br>● Uptake was dependent on heparin, actin, dynamin, and PI3K activity.<br>● EV delivery promoted cell proliferation and facilitated chemotherapeutic resistance to bortezomib in multiple myeloma cell lines, namely MM1S, RPMI 8226, and U266.	[27]

		MSC-derived EVs<br><br>HSPC:Cholesterol liposomes	MSCs<br><br>NIH3T3 cells	5 µg mL^{-1}	2 h	n.r.	67% inhibition<br><br>62% inhibition<br><br>(in MSCs, NIH3T3 had similar values)	n.r.	• HSPC:Cholesterol liposomes and MSC-derived EVs were taken up partly in an actin-dependent manner.<br>• EVs exhibited a two-fold higher uptake than liposomes.	[28]
Colchicine	Interference with tubulin.	C-TAT-Cholesterol:SPC:DSPE-PEG liposomes	B16F1 cells	4 µg mL^{-1}	4 h	n.r.	~40% inhibition	n.r.	• C-TAT-Cholesterol:SPC:DSPE-PEG liposomes were partially taken up through macropinocytosis in B16F1 cells.<br>• In the presence of the exogenous reducing agent glutathione, PEG was cleaved from the surface of liposomes, therefore exposing TAT and ultimately improving cellular uptake.<br>• Loading of liposomes with paclitaxel, presented a tumor inhibition of ~70% in B16F1 tumor-bearing mice.	[26]

n.r. = not reported

- = no inhibition

References

[1] S. Pollock, R. Antrobus, L. Newton, B. Kampa, J. Rossa, S. Latham, N. B. Nichita, R. A. Dwek, N. Zitzmann. *FASEB J* **2010**, *24*, 1866-1878.
[2] U. S. Huth, R. Schubert, R. Peschka-Suss. *J Control Release* **2006**, *110*, 490-504.
[3] F. Cardarelli, D. Pozzi, A. Bifone, C. Marchini, G. Caracciolo. *Mol Pharm* **2012**, *9*, 334-340.
[4] M. Kapoor, D. J. Burgess. *Pharm Res* **2013**, *30*, 1161-1175.
[5] A. Ruyra, M. Cano-Sarabia, S. A. Mackenzie, D. Maspoch, N. Roher. *PLoS One* **2013**, *8*, e76338.
[6] X. X. Zhang, P. G. Allen, M. Grinstaff. *Mol Pharm* **2011**, *8*, 758-766.
[7] N. Furuta, K. Tsuda, H. Omori, T. Yoshimori, F. Yoshimura, A. Amano. *Infect Immun* **2009**, *77*, 4187-4196.
[8] H. Costa Verdera, J. J. Gitz-Francois, R. M. Schiffelers, P. Vader. *J Control Release* **2017**, *266*, 100-108.
[9] T. Tian, Y. L. Zhu, Y. Y. Zhou, G. F. Liang, Y. Y. Wang, F. H. Hu, Z. D. Xiao. *J Biol Chem* **2014**, *289*, 22258-22267.
[10] S. Ahmed, S. Fujita, K. Matsumura. *Nanoscale* **2016**, *8*, 15888-15901.
[11] A. Alshehri, A. Grabowska, S. Stolnik. *Sci Rep* **2018**, *8*, 3748.
[12] D. Montizaan, K. Yang, C. Reker-Smit, A. Salvati. *Nanomedicine* **2020**, *30*, 102300.
[13] J. Gilleron, W. Querbes, A. Zeigerer, A. Borodovsky, G. Marsico, U. Schubert, K. Manygoats, S. Seifert, C. Andree, M. Stoter, H. Epstein-Barash, L. Zhang, V. Koteliansky, K. Fitzgerald, E. Fava, M. Bickle, Y. Kalaidzidis, A. Akinc, M. Maier, M. Zerial. *Nat Biotechnol* **2013**, *31*, 638-646.
[14] M. Lu, X. Zhao, H. Xing, Z. Xun, S. Zhu, L. Lang, T. Yang, C. Cai, D. Wang, P. Ding. *Int J Pharm* **2018**, *550*, 100-113.
[15] T. Asai, T. Tsuzuku, S. Takahashi, A. Okamoto, T. Dewa, M. Nango, K. Hyodo, H. Ishihara, H. Kikuchi, N. Oku. *Biochem Biophys Res Commun* **2014**, *444*, 599-604.
[16] I. A. Khalil, K. Kogure, S. Futaki, H. Harashima. *J Biol Chem* **2006**, *281*, 3544-3551.
[17] S. C. Maddila, C. Voshavar, P. Arjunan, R. P. Chowath, H. K. R. Rachamalla, B. Balakrishnan, P. Balasubramanian, R. Banerjee, S. Marepally. *Molecules* **2021**, *26*.
[18] X. Zhou, Y. Miao, Y. Wang, S. He, L. Guo, J. Mao, M. Chen, Y. Yang, X. Zhang, Y. Gan. *J Extracell Vesicles* **2022**, *11*, e12198.
[19] I. Nakase, N. Ueno, M. Katayama, K. Noguchi, T. Takatani-Nakase, N. B. Kobayashi, T. Yoshida, I. Fujii, S. Futaki. *Chem Commun (Camb)* **2016**, *53*, 317-320.
[20] I. Nakase, N. B. Kobayashi, T. Takatani-Nakase, T. Yoshida. *Sci Rep* **2015**, *5*, 10300.
[21] I. Nakase, K. Noguchi, A. Aoki, T. Takatani-Nakase, I. Fujii, S. Futaki. *Sci Rep* **2017**, *7*, 1991.
[22] I. Nakase, K. Noguchi, I. Fujii, S. Futaki. *Sci Rep* **2016**, *6*, 34937.
[23] S. Kamerkar, V. S. LeBleu, H. Sugimoto, S. Yang, C. F. Ruivo, S. A. Melo, J. J. Lee, R. Kalluri. *Nature* **2017**, *546*, 498-503.
[24] D. Fitzner, M. Schnaars, D. van Rossum, G. Krishnamoorthy, P. Dibaj, M. Bakhti, T. Regen, U. K. Hanisch, M. Simons. *J Cell Sci* **2011**, *124*, 447-458.
[25] M. Jiang, L. Gan, C. Zhu, Y. Dong, J. Liu, Y. Gan. *Biomaterials* **2012**, *33*, 7621-7630.
[26] H. Fu, K. Shi, G. Hu, Y. Yang, Q. Kuang, L. Lu, L. Zhang, W. Chen, M. Dong, Y. Chen, Q. He. *J Pharm Sci* **2015**, *104*, 1160-1173.
[27] C. Tu, Z. Du, H. Zhang, Y. Feng, Y. Qi, Y. Zheng, J. Liu, J. Wang. *Theranostics* **2021**, *11*, 2364-2380.
[28] S. Le Saux, H. Aarrass, J. Lai-Kee-Him, P. Bron, J. Armengaud, G. Miotello, J. Bertrand-Michel, E. Dubois, S. George, O. Faklaris, J. M. Devoisselle, P. Legrand, J. Chopineau, M. Morille. *Biomaterials* **2020**, *231*, 119675.
